# The genome wide analysis of *Tryptophan Aminotransferase Related* gene family, and their relationship with related agronomic traits in *Brassica napus*

**DOI:** 10.3389/fpls.2022.1098820

**Published:** 2022-12-21

**Authors:** Xin Cheng, Xinmin Liu, Jianjie He, Mi Tang, Huaixin Li, Maoteng Li

**Affiliations:** ^1^ College of Life Science and Technology, Huazhong University of Science and Technology, Wuhan, China; ^2^ Key Laboratory of Molecular Biophysics, the Ministry of Education of China, Wuhan, China

**Keywords:** genome-wide identification, *TAR* gene family, agronomic traits, *Brassica napus*, phylogenetic analysis

## Abstract

*Tryptophan Aminotransferase of Arabidopsis1*/*Tryptophan Aminotransferase*-Related (*TAA1*/*TAR*) proteins are the enzymes that involved in auxin biosynthesis pathway. The *TAA1*/*TAR* gene family has been systematically characterized in several plants but has not been well reported in *Brassica napus*. In the present study, a total of 102 *BnTAR* genes with different number of introns were identified. It was revealed that these genes are distributed unevenly and occurred as clusters on different chromosomes except for A4, A5, A10 and C4 in *B. napus*. Most of the these *BnTAR* genes are conserved despite of existing of gene loss and gene gain. In addition, the segmental replication and whole-genome replication events were both play an important role in the *BnTAR* gene family formation. Expression profiles analysis indicated that the expression of *BnTAR* gene showed two patterns, part of them were mainly expressed in roots, stems and leaves of vegetative organs, and the others were mainly expressed in flowers and seeds of reproductive organs. Further analysis showed that many of *BnTAR* genes were located in QTL intervals of oil content or seed weight, for example *BnAMI10* was located in *cqOC-C5-4* and *cqSW-A2-2*, it indicated that some of the *BnTAR* genes might have relationship with these two characteristics. This study provides a multidimensional analysis of the *TAA1/TAR* gene family and a new insight into its biological function in *B. napus*.

## Introduction

Auxin is an essential phytohormone and controls almost every aspect during plant development (Paque and Weijers, 2016). At cellular level, auxin could stimulate cell division of cambium and cell elongation of branch, promote cell differentiation of xylem and phloem, and regulate callus morphogenesis (Paque and Weijers, 2016). At the organ and whole plant level, auxin could affect almost all developmental steps in plants from early embryogenesis to fruit ripening, and control organogenesis at the meristems, which determines the plant structure (Paque and Weijers, 2016). In addition, the application of auxin could reduce the lipid peroxidation of plant cells, thereby reducing the consumption of unsaturated fatty acids (Dhindsa et al., 1984). Meanwhile, it was also found that the biomass and oil yield was increased by adding appropriate amount of auxin in microalgal (Chang et al., 2022).

Indoleacetic acid (IAA) is the best-studied naturally active auxin. IAA is biosynthesized through two major ways: tryptophan (Trp)-dependent and Trp-independent pathways (Woodward and Bartel, 2005). The indole-3-pyruvic acid (IPyA) pathway, which is a tryptophan (Trp)-dependent pathway, appears to be the main contributing factor to the formation of free IAA (Zhao, 2012). There are two steps in the IPyA pathway to converse the Trp to IAA: The first step is catalyzed by the *Tryptophan Aminotransferase of Arabidopsis 1* (*TAA1*), which belongs to *TAA1*-Related (*TAA1/TAR*) family of Trp aminotransferases that transfers the Trp to an alpha-keto acid, and to generate IPyA and another amino acid like L-glutamate (Zhao, 2012). The second step is transferring IPyA to IAA by an oxygen and NADPH-dependent reaction, which is catalyzed by the YUCCA (YUC) family of flavin monooxygenases (Zhao, 2012). Trp-independent pathways have not been fully explored yet, it was reported that some enzymes like Trp synthase α (TSA1) and indole synthase (INS) can catalyze the cleavage of indole 3-glycerol phosphate (IGP) to form indole and D-glyceraldehyde 3-phosphate (G_3_P), and indole was transformed into IAA (Zhang R. et al., 2008). In Arabidopsis genome, four genes were found to have close relationship with *TAA1*, which were referred as *Tryptophan Aminotransferase Related 1*-*4* (*TAR1*, *TAR2*, *TAR3*, *and TAR4*) (Stepanova et al., 2008). It was revealed that the *BnTAR* proteins could classify into two families (Alliinase_C and Aminotran_1_2) according to the primary sequence and homology, which both belong to the superfamily of pyridoxal-5’-phosphate (PLP) dependent enzymes (Liepman and Olsen, 2004). The lack of the Epidermal Growth Factor (EGF) domain suggests that these proteins are not typical alliinases, although they share high sequence similarities with the EGF-alliinase group of C-S lyases (Stepanova et al., 2008).

*B. napus* is an important oil crop, it was an allotetraploid species that derived from the hybridization of *B. rapa* and *B. oleracea* in about 7500 years ago, its genome was contained many duplications as well as inversions and translocations (Chalhoub et al., 2014). Genomic differentiation events that resulted from whole-genome triplication (WGT) was occurred in Brassica species (Cheng et al., 2014). Structural and functional divergence of duplicate genes were also found in other gene families, such as the basic/helix-loop-helix (bHLH) gene family, the ionotropic glutamate receptor (iGluR) gene family, and the Receptor-like kinases (RLKs) gene family (Chiu et al., 2002; Shiu and Bleecker, 2003; Toledo-Ortiz et al., 2003). The characteristics of the duplicate genes suggested that the evolution could have caused an adaptive structural diversification, and this process was pervasive and could have contributed to the biological novelty in plants (Li et al., 2009). Thus, many duplicate genes have similar gene sequences but different functional performances (Liang et al., 2017).

Although there is one study on transaminases involved the *TAA1/TAR* protein of *B. napus* (Le Deunff et al., 2019), which suggests that *TAA1/TAR* not only play an important role in IAA biosynthesis but also in the plant nitrogen cycle. The previous studies have revealed that knock down of *TAA1/TAR* could lead to reduced plant height, decreased apical dominance, valveless gynoecia, vasculature defects, reduced organ number and complete sterility in *Arabidopsis thaliana* (Stepanova et al., 2008). Otherwise, over-expression of *TaTAR* could promote the root elongation (Shao et al., 2017). In addition, *TAA1/TAR* is also involved in ethylene response, it was revealed that the *TAA1/TAR* mutants were showed root-specific ethylene insensitivity in Arabidopsis (Stepanova et al., 2005). However, the gene evolution and structure of the *BnTAR* gene family in *B. napus* have not been reported.

Combined multiple methods to analyses the gene function was frequently used (Kim et al., 2016; Depuydt and Vandepoele, 2021). In our previous studies, many of the QTLs for the seed weight and oil content were identified (Raboanatahiry et al., 2018; Zhao et al., 2019; Yan et al., 2022), for instance, *cqSW-A2-1*, *cqSW-A7-2*, cq*SW-C1-2* and *cqSW-C9-2* for seed weight and *cqOC-A1-1*, *cqOC-A9-11*, *cqOC-C2-2* and *cqOC-C9-4* for oil content. These QTLs provided the convenience for the analysis of the *BnTAR* genes in QTL interval.

In this study, the systematical identification, structure and evolutionary analysis of the *BnTAR* gene family in *B. napus* were analyzed, and the expression patterns of several selected *BnTAR* members in different tissues of *B. napus* and their relationship with seed oil content and yield were also explored, which would provide informative clues to the future functional study.

## Materials and methods

### Identification of TAA1/TAR family genes in B. napus and other species

We used BLAST program to search Ensembl Plants (Ensembl Plants, RRID : SCR_008680) (http://plants.ensembl.org/index.html) (Yates et al., 2022) and 35 *AtTAR* paralogues, which are homologous to *Arabidopsis thaliana AtTAR1* in phylogenetical tree, were identified. *TAA1*/*TAR* genes were identified in *B. napus* based on sequence similarity to the 36 *TAR* protein sequences of Arabidopsis by using the BLAT search program (BLAT, RRID : SCR_011919) in CNS-Genoscope database (CNS Genoscope, RRID : SCR_023020) (https://www.genoscope.cns.fr/brassicanapus/) (Chalhoub et al., 2014), and redundant sequences were removed manually. The *TAA1*/*TAR* genes in *B. rapa* and *B. oleracea* were obtained from the Brad database (Brassicaceae Database, RRID : SCR_023019) (http://brassicadb.cn) (Chen et al., 2022). All the candidate *BnTAR* genes were analyzed by using the Pfam database (Pfam, RRID : SCR_004726) (http://pfam.xfam.org/search) (El-Gebali et al., 2019), SMART database (SMART, RRID : SCR_005026) (http://smart.embl-heidelberg.de/) (Letunic et al., 2004) and NCBI Conserved Domain Search database (Conserved Domains Search, RRID : SCR_018729) (http://www.ncbi.nlm.nih.gov/Structure/cdd/wrpsb.cgi) (Lu et al., 2020) to confirm whether they are belonging to *TAA1*/*TAR* family or not. The categories of *TAA1*/*TAR* gene family in *B. napus* were using the Pfam analysis. For convenience, a univocal name is assigned to each *BnTAR* gene. For example, in the gene of ‘*BnAMI1*’, the first two letters denote the source organism *Brassica napus*, following by the family name and the gene number index (Ostergaard and King, 2008).

The *TAR* genes were also identified in other higher plants in order to trace the evolutionary origin of this family in Phytozome (Phytozome, RRID : SCR_006507) (https://phytozome-next.jgi.doe.gov/) (Goodstein et al., 2012), including of *Amborella Trichopoda*, *Arabidopsis Thaliana*, *Brachypodium distachyon*, *Brassica oleracea*, *Brassica rapa*, *Chromochloris Zofingiensis*, *Glycine Max*, *Gossypium Hirsutum*, *Malus Domestica*, *Oryza Sativa* and *Zea Mays.* In addition, NCBI BLAST (NCBI BLAST, RRID : SCR_004870) (https://blast.ncbi.nlm.nih.gov/Blast.cgi) (Altschul et al., 1990) was also used to search the *TAA1*/*TAR* family genes.

The chromosome locations, CDS lengths and the number of amino acids of the *BnTAR* genes were obtained from the CNS-Genoscope database (Chalhoub et al., 2014). The molecular weight (Mw) and isoelectric point (pI) were calculated by the Compute pI/Mw tool of ExPASy (ExPASy Bioinformatics Resource Portal, RRID : SCR_012880) (https://web.expasy.org/compute_pi/) (Bjellqvist et al., 1993; Bjellqvist et al., 1994). The grand average of hydropathy values (GRAVY) were calculated using the protein hydrophilicity analysis tool (protein hydrophilicity analysis tool, RRID : SCR_023015) (http://www.detaibio.com/sms2/protein_gravy.html) (Kyte and Doolittle, 1982). Subcellular location prediction was conducted using the Plant-mPLoc2.0 (Plant mPLoc, RRID : SCR_023014) (http://www.csbio.sjtu.edu.cn/bioinf/plant-multi/) (Chou and Shen, 2010) and Multi Loc2 (Multi Loc2, RRID : SCR_023013) (https://abi-services.informatik.uni-tuebingen.de/multiloc2/webloc.cgi) (Blum et al., 2009).

### Multiple sequence alignment, phylogenetic and structural analysis

Multiple sequence alignment of all selected *BnTAR*, *BrTAR* and *BoTAR* protein sequences was conducted by using ClustalW (ClustalW, RRID : SCR_017277) in MEGA-X (MEGA, RRID : SCR_023017) (Higgins and Sharp, 1988; Tamura et al., 2013). The unrooted phylogenetic tree of full-length *BnTAR* protein sequences was constructed by using the MEGA-X with Neighbor-Joining (NJ) method (Saitou and Nei, 1987), and the bootstrap analysis was performed using 1,000 replicates.

Based on the alignments of their coding sequences with the corresponding genomic sequences, the exon-intron structures of the *BnTAR* genes were determined and a diagram was drawn by using Gene structure display server (Gene Structure Display Server, RRID : SCR_023011) (GSDS; http://gsds.gao-lab.org/) (Hu et al., 2015). The conserved motif structures of *BnTAR* genes were searched by using Multiple Expectation Maximization for Motif Elicitation (MEME Suite - Motif-based sequence analysis tools, RRID : SCR_001783) (http://alternate.meme-suite.org/) (Bailey et al., 2009). Secondary structural analysis was carried out by using the two following tools: PBIL GOR4 (PBIL GOR4, RRID : SCR_023012) (https://npsa-prabi.ibcp.fr/cgi-bin/npsa_automat.pl?page=npsa_gor4.html) (Jones, 1999) and PSIPRED (PSIPRED, RRID : SCR_010246) (http://bioinf.cs.ucl.ac.uk/psipred/) (Buchan et al., 2013). The Tertiary structures of the *BnTAR*s were predicted using Phyre2 (Phyre, RRID : SCR_010270) (http://www.sbg.bio.ic.ac.uk/phyre2/html/page.cgi?id=index) (Kelley and Sternberg, 2009) and analyzed by VAST (Vector Alignment Search Tool, RRID : SCR_010655) (https://www.ncbi.nlm.nih.gov/Structure/VAST/vastsearch.html) (Gibrat et al., 1996).

### Chromosomal location and gene duplication analysis

The chromosomal locations of the *BnTAR* genes were obtained from the CNS-Genoscope. Neighboring to homologous *BnTAR* genes on *B. napus* chromosomes or within a sequence distance of 50 kb were defined as tandemly duplicated *BnTAR* genes (Yu et al., 2014). According to the definition of tandemly duplicated genes, the genes with a distance of less than 50kb between the nearest genes as gene clusters. The synteny relationships between the *BnTAR*s and *AtTAR*s, *BrTAR*s, and *BoTAR*s were assessed by using the syntenic genes searching tool in BRAD database (http://brassicadb.org/brad/) (Wang et al., 2011) and the synteny tool in the *B. napus* Genome Browser (Chalhoub et al., 2014).

### Calculation of the Ka/Ks

The paralogue pairs were determined according to the homology between *A. thaliana* and *B. napus*. The synonymous substitution rate and the non-synonymous substitution rate of all gene pairs were calculated by TBtools (TBtools, RRID : SCR_023018) (Toolbox for Biologists) (Chen et al., 2020).

### Expression analysis of *TAA1*/*TAR* family genes in the intervals of QTLs for seed oil content and seed weight

The expression data of *TAR* family genes in various tissues and periods in *B. napus* could be accessed from BnTIR (BnTIR, RRID : SCR_023021) (http://yanglab.hzau.edu.cn/BnTIR) (Liu et al., 2021). The transcriptome data of seeds with different oil content or weight were obtained from the previous study of our group and can be found in the SRA database of NCBI (accessions for these SRA data: PRJNA661261 and PRJNA730112).

The QTLs from KN population for seed oil content and seed weight in *B. napus* were obtained from the previous published papers in our group (Raboanatahiry et al., 2018; Zhao et al., 2019; Yan et al., 2022), were used for determine the whether the *TAA1*/*TAR* family genes were located in the QTL interval or not. The KN population was derived from the microspore culture of F1 hybrids that derived from the hybridization between KenC-8 and N53-2, the oil content was about 40% and 50% in all cultivated environments, respectively (Wang et al., 2013), and the thousand seed weight was about 3.53 g and 3.81g in Dali of Shaanxi Province, respectively (Zhao et al., 2016). The genetic distances of the scanned QTLs are converted into physical distances according to the corresponding table (**Table S8**), in which the marker sequence is blasted on the genome to obtain the physical distance corresponding to the genetic distance. The start and end loci of the *BnTAR* genes were compared to the QTL interval to determine whether they were located within the QTL interval or not.

### RNA extraction and RT-qPCR analysis

An RNAprep Pure Plant Plus Kit (Tiangen) was used to separate the total RNA from each frozen sample, and then ReverTra Ace^®^ qPCR RT Master Mix with gDNA Remover (TOYOBO) was used to synthesize the first strand cDNA from the RNA according to the manufacturer’s instructions. Each reaction was repeated three times with a reaction volume of 20 μl containing 0.6 μl of gene-specific primers (1.0 μM), 1.0 μl of cDNA, 10 μl of SYBR green (TaKaRa), and 8.4 μl RNase-free double-distilled water. The PCR conditions were set as follows: Stage 1: 95°C for 5 min; stage 2: 40 cycles of 40 cycles, 95°C for 30 seconds, 55°C for 30 seconds, 72°C for 1 minute. In the stage 2, the fluorescence intensity was measured. A housekeeping gene (*TIP41*) was used as a reference for normalization and analyzed by using a Real-Time PCR System StepOne Plus (ABI), and each reaction was performed in triplicate. The primers for mRNA RT–qPCR are listed in **Table S6**.

## Results

### Genome-wide identification of the *BnTAR* superfamily genes in the *B. napus* genome

To identify the possible candidate *BnTAR* genes, we searched the CNS Genoscope database with the *AtTAR* genes for their homologies in *B. napus*. As a result, a total of 102 genes as *BnTAR* gene family members were identified, and it could be classified into Alliinase_C and Aminotran_1_2 two families based on conserved structural domains in Pfam database. The Alliinase_C family includes 12 members (named as from *BnALL1* to *BnALL12*), and the other family includes 90 members (designated as *BnAMI1*- *BnAMI90*) (**Table 1**).

**Table 1:** Genome identification and classification of *TAR*s genes in *B. napus*.

Name	Gene ID	Family	Name	Gene ID	Family
*BnALL1*	BnaA01g14030D	Alliinase_C	*BnAMI40*	BnaA09g45030D	Aminotran_1_2
*BnALL2*	BnaA02g14990D	Alliinase_C	*BnAMI41*	BnaA10g00470D	Aminotran_1_2
*BnALL3*	BnaA08g06520D	Alliinase_C	*BnAMI42*	BnaAnng08090D	Aminotran_1_2
*BnALL4*	BnaC08g07070D	Alliinase_C	*BnAMI43*	BnaAnng22050D	Aminotran_1_2
*BnALL5*	BnaAnng22040D	Alliinase_C	*BnAMI44*	BnaAnng30490D	Aminotran_1_2
*BnALL6*	BnaC01g16530D	Alliinase_C	*BnAMI45*	BnaAnng31450D	Aminotran_1_2
*BnALL7*	BnaC02g19980D	Alliinase_C	*BnAMI46*	BnaC01g01270D	Aminotran_1_2
*BnALL8*	BnaC06g07950D	Alliinase_C	*BnAMI47*	BnaC01g04560D	Aminotran_1_2
*BnALL9*	BnaC06g43720D	Alliinase_C	*BnAMI48*	BnaC01g10020D	Aminotran_1_2
*BnALL10*	BnaC05g18610D	Alliinase_C	*BnAMI49*	BnaC01g10040D	Aminotran_1_2
*BnALL11*	BnaA09g31200D	Alliinase_C	*BnAMI50*	BnaC01g15060D	Aminotran_1_2
*BnALL12*	BnaA09g31180D	Alliinase_C	*BnAMI51*	BnaC01g18250D	Aminotran_1_2
*BnAMI1*	BnaA01g00270D	Aminotran_1_2	*BnAMI52*	BnaC02g13860D	Aminotran_1_2
*BnAMI2*	BnaA01g03290D	Aminotran_1_2	*BnAMI53*	BnaC02g15560D	Aminotran_1_2
*BnAMI3*	BnaA01g08470D	Aminotran_1_2	*BnAMI54*	BnaC02g16260D	Aminotran_1_2
*BnAMI4*	BnaA01g13270D	Aminotran_1_2	*BnAMI55*	BnaC02g20870D	Aminotran_1_2
*BnAMI5*	BnaA01g13280D	Aminotran_1_2	*BnAMI56*	BnaC02g23160D	Aminotran_1_2
*BnAMI6*	BnaA01g15380D	Aminotran_1_2	*BnAMI57*	BnaC03g26850D	Aminotran_1_2
*BnAMI7*	BnaA02g09960D	Aminotran_1_2	*BnAMI58*	BnaC03g28000D	Aminotran_1_2
*BnAMI8*	BnaA02g11830D	Aminotran_1_2	*BnAMI59*	BnaC03g45280D	Aminotran_1_2
*BnAMI9*	BnaA02g15650D	Aminotran_1_2	*BnAMI60*	BnaC03g66430D	Aminotran_1_2
*BnAMI10*	BnaA02g19790D	Aminotran_1_2	*BnAMI61*	BnaC03g76570D	Aminotran_1_2
*BnAMI11*	BnaA03g22790D	Aminotran_1_2	*BnAMI62*	BnaC04g35390D	Aminotran_1_2
*BnAMI12*	BnaA03g23600D	Aminotran_1_2	*BnAMI63*	BnaC05g00530D	Aminotran_1_2
*BnAMI13*	BnaA03g38440D	Aminotran_1_2	*BnAMI64*	BnaC05g13450D	Aminotran_1_2
*BnAMI14*	BnaA03g38470D	Aminotran_1_2	*BnAMI65*	BnaC05g18600D	Aminotran_1_2
*BnAMI15*	BnaA03g40560D	Aminotran_1_2	*BnAMI66*	BnaCnng71530D	Aminotran_1_2
*BnAMI16*	BnaA03g46060D	Aminotran_1_2	*BnAMI67*	BnaC06g19110D	Aminotran_1_2
*BnAMI17*	BnaA03g47880D	Aminotran_1_2	*BnAMI68*	BnaC06g20760D	Aminotran_1_2
*BnAMI18*	BnaA03g49250D	Aminotran_1_2	*BnAMI69*	BnaC06g32920D	Aminotran_1_2
*BnAMI19*	BnaA04g13310D	Aminotran_1_2	*BnAMI70*	BnaC06g38270D	Aminotran_1_2
*BnAMI20*	BnaA05g12790D	Aminotran_1_2	*BnAMI71*	BnaC06g40630D	Aminotran_1_2
*BnAMI21*	BnaA06g11610D	Aminotran_1_2	*BnAMI72*	BnaC06g43710D	Aminotran_1_2
*BnAMI22*	BnaA06g15630D	Aminotran_1_2	*BnAMI73*	BnaC07g13140D	Aminotran_1_2
*BnAMI23*	BnaA07g00460D	Aminotran_1_2	*BnAMI74*	BnaC07g31520D	Aminotran_1_2
*BnAMI24*	BnaA07g10020D	Aminotran_1_2	*BnAMI75*	BnaC07g40130D	Aminotran_1_2
*BnAMI25*	BnaA07g29720D	Aminotran_1_2	*BnAMI76*	BnaC07g41260D	Aminotran_1_2
*BnAMI26*	BnaA07g35690D	Aminotran_1_2	*BnAMI77*	BnaC07g41280D	Aminotran_1_2
*BnAMI27*	BnaA07g38380D	Aminotran_1_2	*BnAMI78*	BnaC08g06270D	Aminotran_1_2
*BnAMI28*	BnaA08g10670D	Aminotran_1_2	*BnAMI79*	BnaC08g17310D	Aminotran_1_2
*BnAMI29*	BnaA08g11470D	Aminotran_1_2	*BnAMI80*	BnaC08g21300D	Aminotran_1_2
*BnAMI30*	BnaA08g20540D	Aminotran_1_2	*BnAMI81*	BnaC08g33730D	Aminotran_1_2
*BnAMI31*	BnaA08g23190D	Aminotran_1_2	*BnAMI82*	BnaC08g36300D	Aminotran_1_2
*BnAMI32*	BnaA09g07210D	Aminotran_1_2	*BnAMI83*	BnaC08g37860D	Aminotran_1_2
*BnAMI33*	BnaA09g10030D	Aminotran_1_2	*BnAMI84*	BnaC08g49010D	Aminotran_1_2
*BnAMI34*	BnaA09g13010D	Aminotran_1_2	*BnAMI85*	BnaC09g07070D	Aminotran_1_2
*BnAMI35*	BnaA09g21860D	Aminotran_1_2	*BnAMI86*	BnaC09g10110D	Aminotran_1_2
*BnAMI36*	BnaA09g31220D	Aminotran_1_2	*BnAMI87*	BnaC09g12920D	Aminotran_1_2
*BnAMI37*	BnaA09g39260D	Aminotran_1_2	*BnAMI88*	BnaC09g24050D	Aminotran_1_2
*BnAMI38*	BnaA09g41170D	Aminotran_1_2	*BnAMI89*	BnaCnng43090D	Aminotran_1_2
*BnAMI39*	BnaA09g43670D	Aminotran_1_2	*BnAMI90*	BnaCnng64190D	Aminotran_1_2

The properties of *BnTAR* were analyzed by Expasy database. All the *BnTAR* proteins had molecular weights less than 70 kDa, but the isoelectric points varied in a wide range, which are from 4.78 to 9.68 (**Table S1**). The hydrophilicity of *BnTAR*s was assessed based on the grand average of hydropathy (GRAVY) value (**Table S1**). On average, the GRAVY values of *BnALL* proteins are higher than those of *BnAMI* proteins, it indicated the higher hydrophilicity of *ALL* proteins. Meanwhile, almost all the *BnTAR*s with the GRAVY values less than zero, which demonstrated that most of *BnTAR* proteins are hydrophilic. The subcellular localization of 102 *BnTAR* proteins was predicted by using Plant-mPLoc and Multi Loc2 (**Tables S1 and S2**), it was revealed that most of them were located in the cytoplasm or chloroplast, only a few proteins were predicted in the secretory pathway or the nucleus.

### Sequence alignment and phylogenetic analysis of *BnTAR* genes

**Figure 1:**
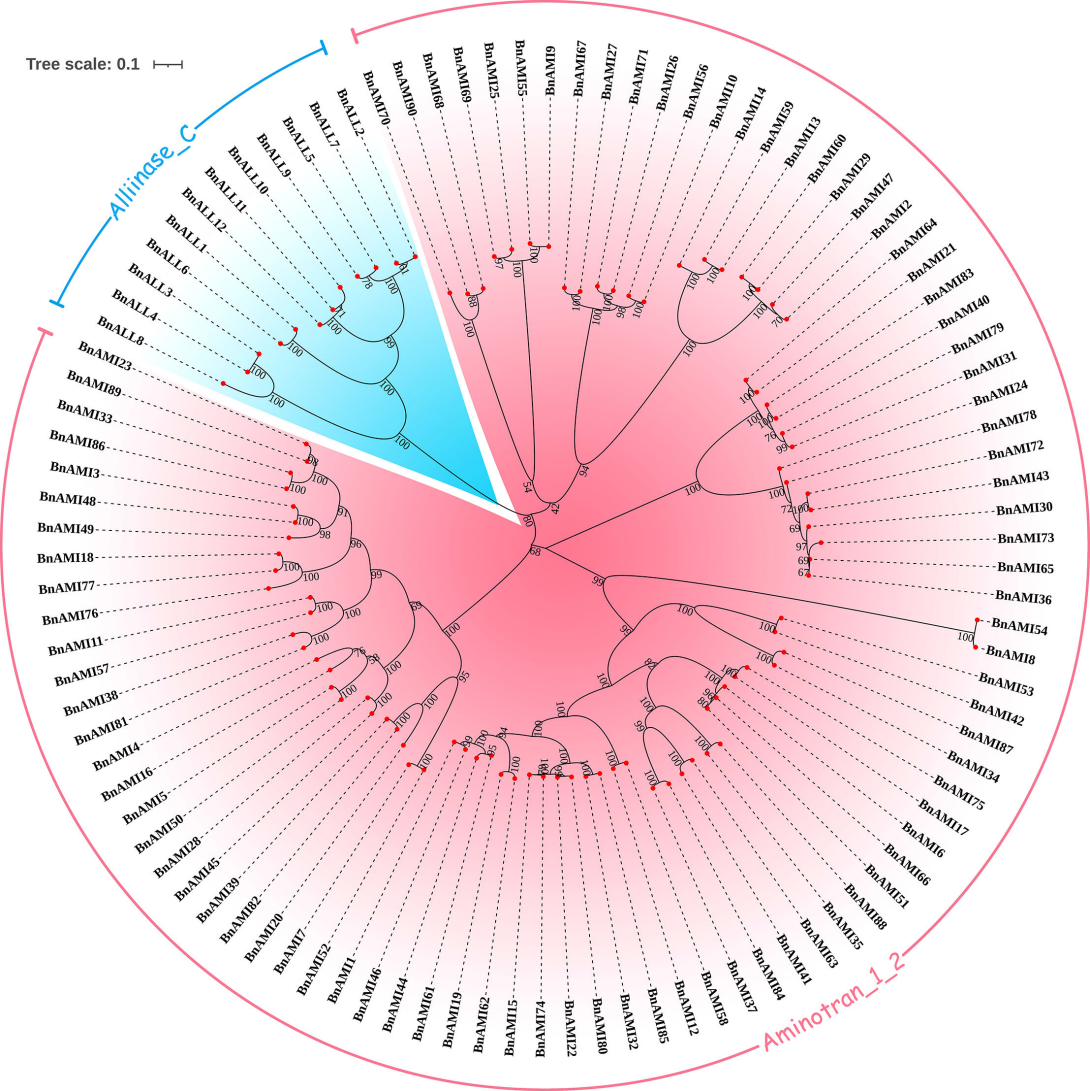
Phylogenetic analysis of the *BnTAR* genes. Different *BnTAR* gene families were marked in different colors.

We performed sequence alignment of *BnTAR* genes and constructed an unrooted phylogenetic tree of 102 *BnTAR* genes. This was used to determine the evolutionary relationships among *BnTAR* genes, and we found that most of the genes formed gene pairs with high Bootstrap support (**Figure 1**), it suggested that the chromosome duplication was contributed significantly to *BnTAR* gene formation. And most of the gene pairs have short branches (**Figure 1**), which might be separated in a more recent time. Aminotran_1_2 was generally divided into four large branches, among which *BnAMI8* and *BnAMI54* are in a separate clade (**Figure 1**). They are distant from the other members of the Aminotran_1_2 family, they might have diverged from other *BnAMI* genes earlier and their function might be more independently. Also, twelve genes of the Alliinase_C family are clustered together and share high similarities (**Figure 1**).

According to the sequence comparison results, protein sequences of members of *BnALL* family are very conserved, with about 30.5%-36.7% of amino acids having over 90% similarity (**Figure S1**). In contrast, sequence conservation of the members of *BnAMI* family is of low level. Among the four large branches of the Aminotran_1_2 family, the branch that contained *BnAMI31* and *BnAMI65* was had the highest internal conserved type (**Figure S1**). About 45.2%-61.5% of the amino acids in this branch were with more than 90% similarity across the sequences, which suggested that these genes were had a high level of functional redundancy. The branch that contained the *BnAMI2* and *BnAMI69* was the least conserved, with about 6.83%-10.0% of amino acids having high similarity except *BnAMI70* which is rather short (**Figure S1**). The large number of variation sites suggested that these genes have important roles in adaptation to ever-changing environment. The branch of *BnAMI39* and *BnAMI86* was with a close number of conserved amino acids to the branch of *BnAMI8* and *BnAMI80* (**Figure S1**), but the overall conservation was lower due to the presence of two genes with longer sequences, *BnAMI8* and *BnAMI54*.

### Chromosomal location and the expansion pattern of the *BnTAR* Genes

**Figure 2:**
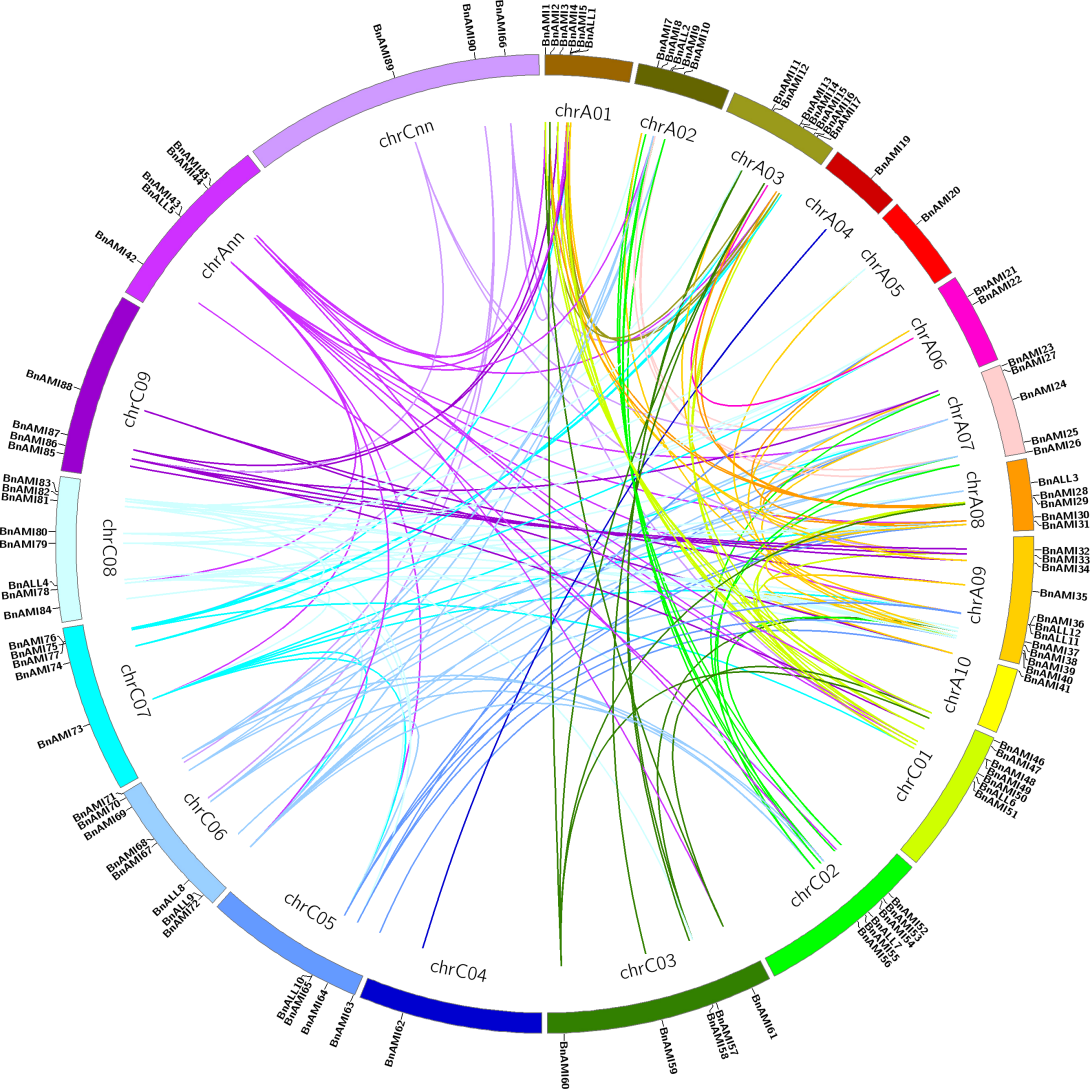
Distribution of *BnTAR* gene family among *B. napus* genome. The chromosomal information of the *BnTAR* genes was mapped to the *B. napus* chromosomes and the synteny relationship was highlighted in different colors.

The chromosomal locations of *BnTAR* genes were analyzed, and 102 *BnTAR* genes that located on the 19 chromosomes of *B. napus* were found (**Figure 2**). However, only one *BnTAR* gene was located on chromosomes A4, A5, A10 and C4, respectively. In contrast, chromosome A9 was with 11 *BnTAR* genes. The *BnTAR* gene arrangement is also relatively concentrated on different chromosomes and some of the *BnTAR* genes were existed as gene clusters, such as *BnAMI4* and *BnAMI5* on A1, *BnAMI48* and *BnAMI49* on C1 (**Figure 2**), which might be caused by the duplication of *BnTAR* genes during the evolutionary history.

To understand the expansion pattern of the *BnTAR* genes in *B. napus*, we investigated gene duplication events. Synteny analysis showed that the *BnTAR* genes are phylogenetically close to *TAR* genes in other three cruciferous species (*B. rapa*, *B. oleracea*, *Arabidopsis thaliana*). There are 36 *TAR* genes in *A. thaliana*, *AT1G23310* was contained eight copies, it was formed by fragment duplication. In addition, *AT1G17290*, *AT1G70580*, *AT1G72330* and *AT1G80360* were all have six copies, it was involved in replication formation. In the synteny analysis of *B. rapa*, *B. oleracea* and *B. napus*, 54 *BrTAR* genes were found in the genome of *B. rapa*, and it showed that at most 7 *BnTAR* genes were corresponding to the same *BrTAR* gene (such as BraA03g055430), and at least 1 *BnTAR* gene corresponding to the same *BrTAR* gene (such as BraA02g021330). 58 *BoTAR* genes were found in the genome of *B. oleracea*, it was also revealed that at most 7 *BnTAR* genes were corresponding to the same *BoTAR* gene (such as BolC07g053890), and at least 1 *BnTAR* gene corresponding to the same *BrTAR* gene (BolC08g046990). Further analysis revealed that those 54 *BrTAR* genes were corresponding to 80 *BnTAR* genes, and 41 and 39 of these *BnTAR* genes are located in A genome and C genome of *B. napus*, respectively. As for 58 *BoTAR* genes, which corresponding to 89 *BnTAR* genes, and 42 and 47 of them were located in A genome and C genome of *B. napus*, respectively. However, 13 *BnTAR* genes were not corresponding to any *BrTAR* genes or *BoTAR* genes. These results indicated that the *BnTAR* gene was highly conservative in the evolutionary process, but it also produces unique *BnTAR* genes, such as *BnAMI5*. These results above demonstrated that segment duplication was played an important role in the evolution of these gene loci.

**Figure 3:**
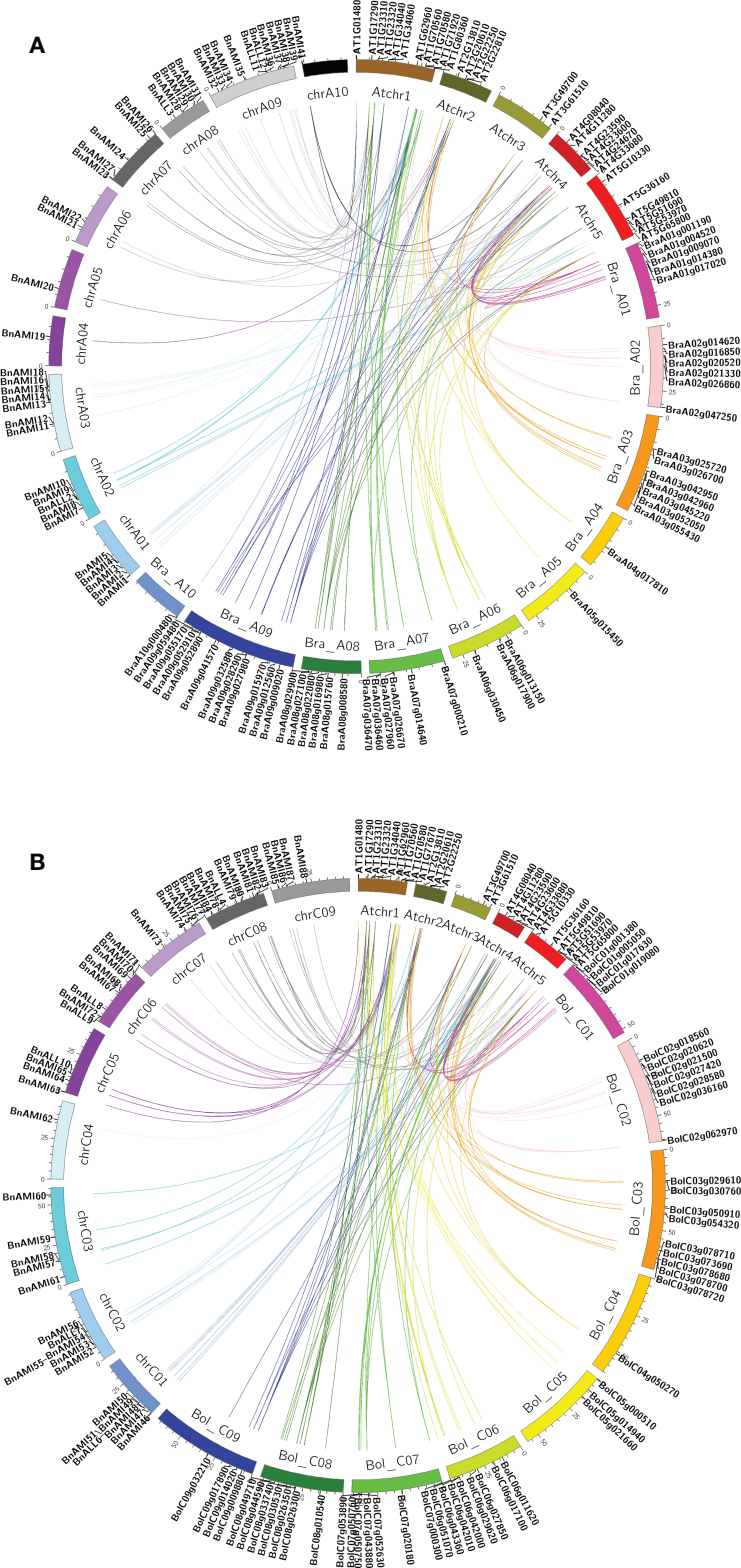
Synteny analysis map of *TAR* genes in *B. napus*, *B. rapa*, *B. oleracea*, and *A. thaliana* chromosomes. **(A)** The synteny of genes located on *B. napus* A subgenome, *B. rapa* genome and *A. thaliana* genome. **(B)** The synteny of genes located on *B. napus* C subgenome, *B. oleracea* genome and *A. thaliana* genome.

By comparing the gene distribution of *TAR* genes in the genomes of *B. rapa*, *B. oleracea*, *A. thaliana* and *B. napus*, we found that the codominance of *AMI* and *ALL* families are maintained, but some genes are duplicated or lost. In addition, the synteny maps of *TAR* genes in *A. thaliana*, *B. rapa*, and A subgenome of *B. napus*, and *TAR* genes in *A. thaliana*, *B. oleracea*, and C subgenome of *B. napus* were analyzed separately (**Figure 3**). The results showed that *TAR* genes were almost evenly distributed in the Arabidopsis genome. These *AtTAR* genes are synteny to *BrTAR* and *BoTAR* genes, it indicated that *AtTAR* genes were replicated and formed into *BrTAR* or *BoTAR*, which was the result of genomic rearrangement after whole-genome triplication (WGT) in Brassicaceae species. In addition, almost all the *BrTAR* or *BoTAR* genes were maintained a synteny relationship with *BnTAR*s.

**Figure 4:**
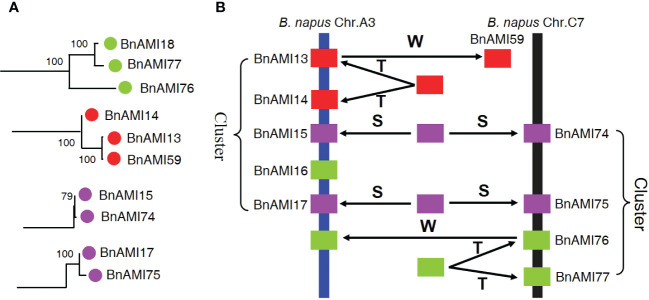
Phylogenetic relationships and hypothetical evolutionary progress of the cluster of *BnTAR* genes in *B. napus* chromosomes A3 and C7. **(A)** Phylogenetic relationships of selected *BnTAR* genes in the cluster. **(B)** Hypothetical mechanism of *BnTAR* gene cluster formation. The letters T, S, and W indicate putative tandem duplication, segmental duplication, and whole-genome duplication, respectively.

There are three mechanisms of gene family expansion: tandem duplication, segmental duplication and whole-genome duplication (WGD) (Xu et al., 2012). The original diploid genomes of Arabidopsis are ancient polyploids, which was undergo massive chromosomal rearrangements in the evolution (Schmidt et al., 2001). *BnAMI13*/*BnAMI14* on A3 chromosome cluster and *BnAMI76*/*BnAMI77* on C7 chromosome cluster are tandem duplicated genes (**Table S1**). Meanwhile, *BnAMI59* is clustered together with *BnAMI13*/*BnAMI14* and *BnAMI18* together with *BnAMI76*/*BnAMI77* (**Figure 1**), respectively, it suggested that these genes might come from the same ancestor. Interestingly, the two tandemly duplicated genes, *BnAMI13* was clustered together with *BnAMI59* but not with *BnAMI14*, and *BnAMI77* was clustered together with *BnAMI18* but not with *BnAMI76* (**Figures 1** and **4A**). This might indicate that these genes were experienced tandem duplication and then whole-genome duplication during the evolutionary process, and then caused the independent genes being more closely related to one of the tandem duplicated genes. In addition, *BnAMI74* and *BnAMI75* might be associated with segmental duplication, as they show synteny relationships with *BnAMI15* and *BnAMI14*, respectively. Phylogenetic analysis also indicated that *BnAMI74*/*BnAMI15* and *BnAMI75*/*BnAMI77* are homologous gene pairs, and they are respectively located in A and C subgenomes (**Figure 4A**), which might come from two different ancestors. Importantly, *BnAMI13*, *BnAMI14*, *BnAMI15*, *BnAMI17* and *BnAMI18* are all on the A3 chromosome cluster, and *BnAMI74*, *BnAMI75*, *BnAMI76* and *BnAMI77* are all on the C7 chromosome cluster, which indicates that the three duplication events play an important role in the formation of *BnTAR* gene clusters (**Figure 4B**).

Meanwhile, non-synonymous (Ka) and synonymous (Ks) values are used to analyze the selection pressure on *BnTAR* genes during the evolutionary process (**Table S3**). The results showed that most of *BnTAR* genes had the Ka/Ks ratio values between 0.1 and 1, it indicated that *BnTAR* has low selection pressure during evolution. We also found that there existed more than one Arabidopsis gene homologous to the same gene of *B. napus*. For instance, two Arabidopsis genes *AT1G70580* and *AT1G23310*, both homologous to *BnAMI78* of *B. napus*, and there are even three Arabidopsis genes, *AT1G01480*, *AT3G61510* and *AT4G11280* are all homologous to *BnAMI41* of *B. napus* (**Table S3**), this might be caused by synonymous substitution. Interestingly, four NaN values can be found in the **Table S3**, including of *AT3G61510*, *AT4G11280* and *BnAMI41* (**Table S3**). The NaN value appears because the pS is higher than 0.75, which means that there are more synonymous substitution sites resulting in higher sequence variability. These *TAR* genes themselves have high sequence similarity in *A. thaliana* and *B. napus*, most of the *TAR* family genes evolved neutrally, leading to a high rate of synonymous substitutions. Ultimately, this leads to a high level of synonymous substitution in *BnAMI41*, which resulted in its correspondence to multiple Arabidopsis duplicate genes. Therefore, there will be many Arabidopsis genes corresponding to the same *B. napus* gene together. These evolutionary relationships found through Ka/Ks ratio together with the homology homozygosity and chromosomal gene localization analyses above suggested that the WGD and segmental duplication play an important role in the evolution of the *TAR* gene family, which is consistent with the evolutionary process in Brassica species.

### Gene structural analysis of *BnTAR*s

Most of *BnTAR* genes are less than 5 kb except for *BnALL10*, *BnAMI8* and *BnAMI54* (**Table S1, Figure S2**). It was revealed that the different branches were with different intron-exon structural features. Most of the *BnALL* genes were contained no more than five introns, while most of the *BnAMI* genes were with more than five introns (**Figure S2**). Particularly, the *BnAMI* genes in the fourth main branch were more distinct from other branches with more than ten introns (**Figure S2**). *BnAMI8* and *BnAMI54*, whose sequence length is larger than 6 kb, were found to have similar intron-exon structural features, it reflected their close phylogenetic relationship (**Figure S2**). And similarly, most of phylogenetical gene pairs have the similar intron-exon structures between each other (**Figure S2**).

**Figure 5:**
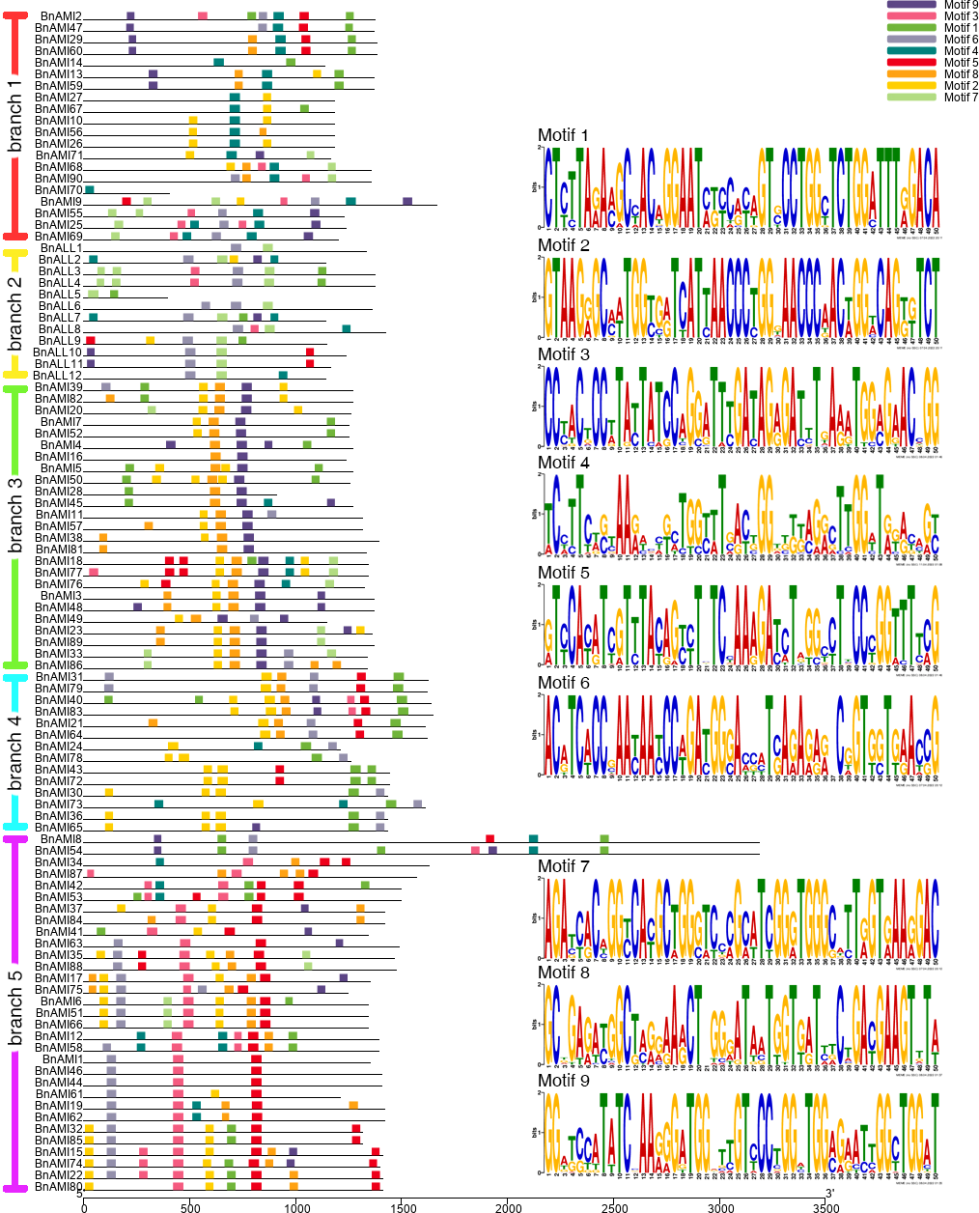
Motif patterns of different *BnTAR* branches and predicted consensus motifs in each *BnTAR* gene branch. Different motifs are shown in different colors. The lengths of the proteins and motifs can be estimated using the scale at the bottom. The motifs in Pfam of each branch are shown on the right.

Due to the low level of similarity of all *BnTAR* genes among the different branches, these genes were submitted to MEME for motif structural analysis, separately. The results showed that 9 conserved motifs were identified, of which the Motif 2 was observed in almost every branch (**Figure 5**). These conserved motifs were belonged to two Pfam domains, as shown in the Pfam codes (Pfam PF00155 and Pfam PF04864) and Web logo. In each phylogenetic branch, most of the gene pairs or genes in close branches have the similar motif composition. Sequence length of *BnALL5* is below 600bp, but it still has two motifs (motif 1 and motif 7) (**Figure 5**), it suggested that the *BnALL5* might had undergone fragment deletion or insertion during evolution, but the conserved motifs are still preserved, which might be essential to its function. The sequence lengths of *BnAMI8* and *BnAMI54* are particularly long, which were with 6 and 8 motifs, respectively (**Figure 5**). The other genes do not differ much in length, and most of them were contained more than five motifs, and in each branch, the types of motifs are roughly similar. These results demonstrated that the different *TAR* genes have different structural compositions, but are similar within one phylogenetic branch and the motifs encoding *TAR* structural domains are conserved.

### Secondary and tertiary structural analysis of *BnTAR*

**Figure 6:**
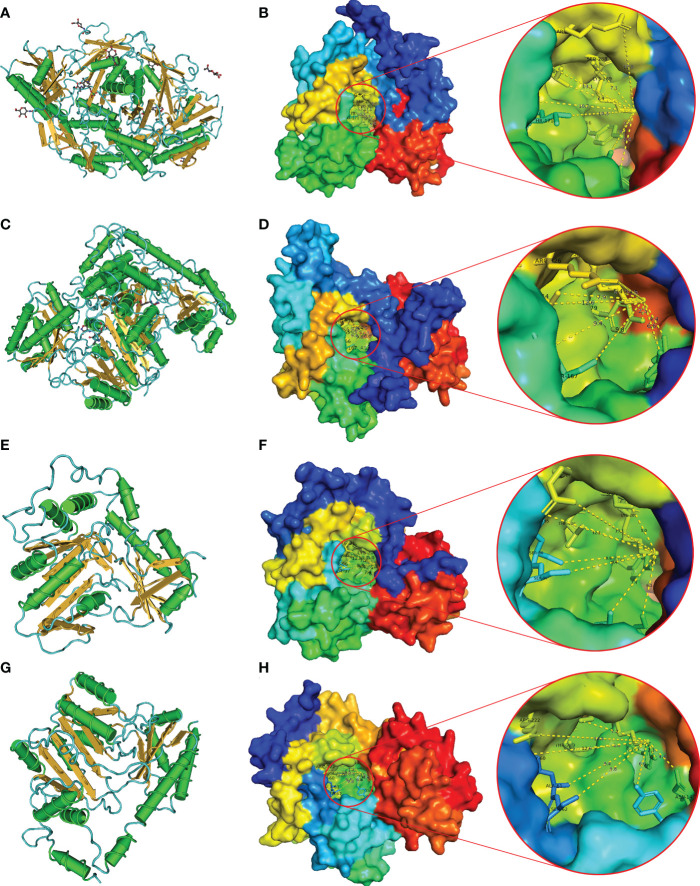
Predicted 3D structure of *BnTAR* genes. 2HOX and 3TCM were selected as examples. The models were predicted by Phyre2. Conserved domain analysis was highlighted using VAST. Red circles indicate the active center of the enzyme. **(A)** Predictive 3D structure of 2HOX in cylinder and plate mood. **(B)** The predicted 3D structure of 2HOX showed in the surface mood. **(C)** Predictive 3D structure of 3TCM in cylinder and plate mood. **(D)** The predicted 3D structure of 3TCM showed in the surface mood. **(E)** Predictive 3D structure of *BnALL10* in cylinder and plate mood **(F)** The predicted 3D structure of *BnALL10* showed in the surface mood. **(G)** Predictive 3D structure of *BnAMI78* in cylinder and plate mood. **(H)** The predicted 3D structure of *BnAMI78* showed in the surface mood.

The protein secondary structure of *BnTAR* was predicted by using the GOR4 and PSIPRED. It was revealed that the *BnTAR* proteins were mainly contained α-helixes, β-folds and random coils. Further analysis showed that the α-helixes, β-folds and random coils was account for about 35%, 20% and 45%, respectively (**Figure S3**). In addition, 3D structures of *BnTAR* proteins were also modeled and predicted by using the Phyre2. Firstly, we use VAST to analyze the protein sequences of all *BnTAR*, and then compared them with the structures that obtained from the PDB database. The predicted structural domains were displayed in the Cn3D macromolecular structure viewer (α-helix in green, β-fold in yellow and random coil in blue) (**Figures 6A, C, E, G** **)**. And the structures of Alliinase from Allium Sativum (garlic) (2HOX) (**Figures 6A, B** **)** and the Crystal Structure of Alanine Aminotransferase from Hordeum vulgare (3TCM) (**Figures 6C, D** **)** were selected for homology modeling, as their structures have been fully studied. In the three-dimensional structure, the *BnTAR* proteins all appeared to be overlapping bowl-shaped, with a hollow in the center of the protein (**Figures 6B, D, F, H** **)**, which is the active center of the enzyme and contains the pyridoxal binding site. The pyridoxal binding sites of *BnTAR*, 2HOX and 3TCM are all contain 10 amino acids, among which the first three amino acids are continuous amino acids, the seventh and eighth amino acids are separated by a random amino acid, the eighth and ninth amino acids are continuous and there are seven amino acids between the ninth and tenth amino acid (**Table S4** **)**. In addition, the ninth amino acid Lys which is very conserved is also the catalytic residue (**Table S4** **)**. These ten amino acids are formed into the conserved hole structure of proteins. At the same time, the difference of amino acid sequence in the protein also leads to a slight difference in the distance from each amino acid to the catalytic residue Lys in the 3D structure (**Table S5** **)**. The distance from the pyridoxal binding site amino acid to the catalytic residue Lys in *BnALL* and 2HOX is farther. For example, the distance from fifth amino acid Asn to Lys of *BnALL10* and 2HOX are 6.1 nm and 6 nm, respectively, otherwise, the *BnAMI78* and 3TCM are 4.4 nm (**Table S5** **)**. These results suggest that *BnTAR* proteins have a conserved structure, but also show some differences in the three-dimensional structural state and the number of secondary structures, which may help them to perform different functions in different environments.

### Expression profiles analysis of *BnTAR* genes in different tissues

In order to fully understand the expression pattern of the *BnTAR*s, the expression of *BnTAR* genes were performed by using the data in the BnTIR database (**Figure S4**). We found that about a third of the *BnTAR* genes were barely expressed in *B. napus*, but there were some genes (such as *BnAMI78*, *BnAMI36*, *BnAMI65*, *BnAMI23*, *BnAMI27*, *BnALL2*) that were highly expressed in specific tissues (**Figure S4**). It was revealed that a part of *BnTAR* genes expressed higher in more actively growing tissues like root, stem and leaf, such as *BnAMI78*, *BnAMI36*, *BnAMI65* and *BnAMI23* (**Figure S4**). Another part of *BnTAR* genes were showed higher expression levels in early developed seeds, such as *BnAMI27*, *BnAMI67*, *BnALL2* and *BnALL7* (**Figure S4**).

**Figure 7:**
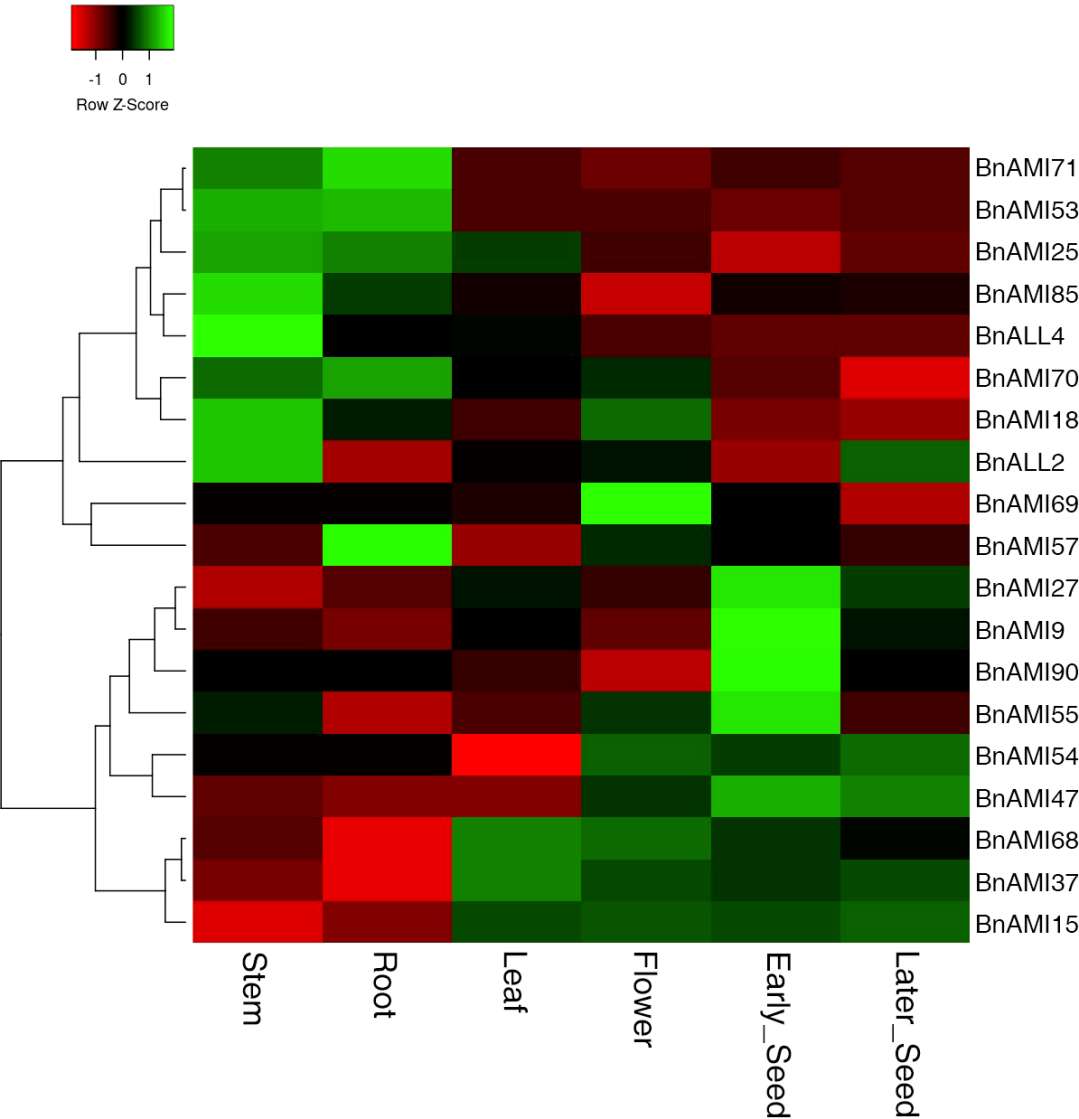
Hierarchical clustering of the expression profiles of *BnTAR* genes in different tissues. The color scale represents relative expression levels with high (green) or low (red). Early-developing seeds were got 14 days after flowering, and later-developing seeds were 27 days after flowering.

To validate the data obtained from the database, 19 *BnTAR* genes were selected for RT-qPCR analysis in order to investigate the expression pattern of *BnTAR* genes in different tissues of *B. napus* (**Tables S6 and S7**). The results indicated that the expression of different *BnTAR* genes in each tissue was different, and the expression pattern of each gene also varies in different tissues (**Figure 7**). It was showed that the expression of *BnTAR* genes could be roughly divided into two main patterns: one is mainly expressed in nutritional organs, such as roots and stems (represented by *BnAMI71* and *BnAMI53*), while the other is mainly expressed in reproductive organs, such as flowers and seeds (characterized by *BnAMI37* and *BnAMI14*). This is consistent with the expression pattern available in the BnTIR database.

### Relevance analysis of *BnTAR* genes and seed oil content and seed weight

**Figure 8:**
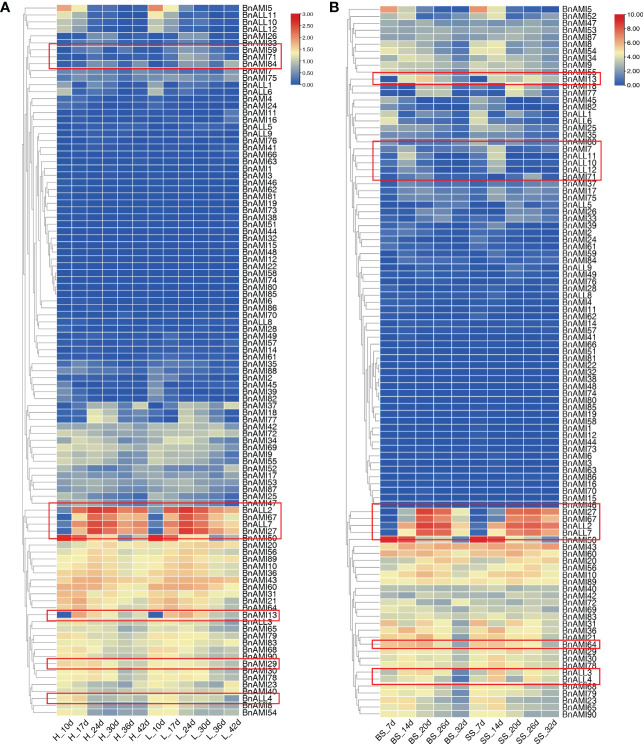
Expression of *BnTAR* gene in transcriptome data. The color scale represents relative expression levels with high (red) or low (blue). Red boxes indicate the genes that are differentially expressed. **(A)** The transcriptome data of high oil samples and low oil samples. ‘H’ means high oil samples while ‘L’ means low oil samples. **(B)** The transcriptome data of different seed weight simples. ‘BS’ means big seed simples and ‘SS’ means small seed simples. The letter ‘d’ all means how many days after flowering.

Based on the transcriptome data of high and low oil content, and the transcriptome data of different seed weights in *B. napus*, we analyzed the contribution of the *BnTAR* genes to various agricultural traits. It was revealed that the expression of most *BnTAR* genes between samples has no obviously differences, and even many genes were barely expressed (**Figure 8**). However, four genes, *BnALL2*, *BnALL7*, *BnAMI27* and *BnAMI67*, were with higher expression, especially in the later seed development stages (36-42 days after flower). It also showed that the expression level in the *B. napus* with high oil content was higher than that of *B. napus* with the low oil content (**Figure 8A**). Further analysis of the expression of these four genes was also significantly different in *B. napus* with different seed weight. It was revealed that the expression was up-regulated at mid-seed development stage (20-26 days after flower) and down-regulated at late development stage (32 days after flower) in relatively large seed *B. napus* (**Figure 8B**). Also, some genes with a certain amount of expression were also found to be differentially expressed in different samples, for example, *BnAMI13* and *BnALL4* have differential expression in high and low oil samples, *BnAMI64* and *BnALL4* have differential expression in different seed weight samples (**Figure 8**). These results suggest that most of the *BnTAR* genes might not contribute directly to seed oil or weight, but there are a number of genes (such as *BnALL2*, *BnALL7*, *BnAMI27* and *BnAMI67*) are involved in influencing seed oil content and seed weight.

In order further analysis the relationship between these *BnTAR* genes with seed oil content and seed weight, the positions of 102 *BnTAR* genes were analyzed in order to make clear whether they were located in the quantitative trait locus (QTL) for seed oil content (Yan et al., 2022) and seed weight (Raboanatahiry et al., 2018; Zhao et al., 2019) that obtained in our group or not. It was revealed that 21 and 12 *BnTAR* genes were found to be located within the QTL interval of seed oil content and seed weight, respectively (**Table 2**). Among them, the *BnAMI10* gene was located in both the *cqOC-A2-3* interval for oil content and the *cqSW-A2-2* interval for seed weight, which contribute to low oil content and small seed weight. It indicated that this gene might have the function for both seed weight and oil content. Also, many genes were found to be located in QTL intervals that obtained in multiple environments, such as interval of *cqOC-A9-16* where the *BnAMI40* gene is located, the phenotypic value to oil content was found to be significant with a maximum of 12.47% and a minimum of 9.84% and contribute to high oil content in multiple environments. Meanwhile, for seed weight, the interval of *cqSW-C6-4* and *cqSW-C9-6*, where *BnAMI69* and *BnAMI86*/*BnAMI87* are located, both with the phenotypic value more than 8% to seed weight. The *cqSW-C6-4* interval has an important contribution to seed weight enlargement, while *cqSW-C9-6* contributes to seed weight reduction. At the same time, during the comparison with the transcriptome data, we found that some genes that showed significant differential expression in the transcriptome (e.g., *BnALL2*, *BnALL3*, *BnAMI10*, *BnAMI67*, etc.) were also located in the corresponding QTL intervals, which corroborates our results for the transcriptome analysis (**Figure 8**). These results indicated that many of *BnTAR* genes were more or less involved in oil formation and seed development in *B. napus*.

**Table 2:** Analysis of the QTL interval of the *BnTAR* genes.

QTL for oil content (OC)				QTL for thousand seed weight (TSW)				
Name	Consensus QTL*	LOD	R²	Name	Consensus QTL**	LOD		R²
*BnALL1*	*cqOC-A1-1*	3.00328-4.42897	3.3939-9.5762	*BnALL6*	*cqSW-C1-2*	3.90817		5.4284
*BnALL2*	*cqOC-A2-2*	7.62538	14.0549	*BnAMI10*	*cqSW-A2-2*	2.5172		4.574
*BnALL3*	*cqOC-A8-2*	2.95337-5.55303	3.473-7.3556	*BnAMI23*	*cqSW-A7-1*	2.55409		3.0467
*BnALL8*	*cqOC-C6-1*	2.46946	2.2607	*BnAMI34*	*cqSW-A9-4*	3.69334-5.23404		4.3782-5.5929
*BnALL10*	*cqOC-C5-4*	3.07706	4.1087	*BnAMI50*	*cqSW-C1-5*	4.0796		5.1477
*BnAMI6*	*cqOC-A1-1*	3.00328-4.42897	3.3939-9.5762	*BnAMI51*	*cqSW-C1-3*	3.57399-3.90817		5.2212-5.705
*BnAMI7*	*cqOC-A2-1*	2.95988	3.2563	*BnAMI69*	*cqSW-C6-4*	7.51905		8.9761
*BnAMI9*	*cqOC-A2-3*	3.03583-7.8337	3.5296-16.3876	*BnAMI70*	*cqSW-C6-7*	5.36207		7.3724
*BnAMI10*	*cqOC-A2-3*	3.03583-7.8337	3.5296-16.3876	*BnAMI85*	*cqSW-C9-2*	4.98232		6.2849
*BnAMI13*	*cqOC-A3-8*	2.98809-3.30057	2.8245-3.7503	*BnAMI86*	*cqSW-C9-6*	5.15809		8.7772
*BnAMI14*	*cqOC-A3-8*	2.98809-3.30057	2.8245-3.7503	*BnAMI87*	*cqSW-C9-6*	5.15809		8.7772
*BnAMI28*	*cqOC-A8-3*	5.29263-7.03948	6.1033-7.176	*BnAMI88*	*cqSW-C9-8*	3.53059		5.3449
*BnAMI29*	*cqOC-A8-3*	5.29263-7.03948	6.1033-7.176					
*BnAMI32*	*cqOC-A9-5*	2.82534-7.52773	3.6503-9.0369					
*BnAMI33*	*cqOC-A9-7*	6.45358	6.5969					
*BnAMI35*	*cqOC-A9-9*	3.76929	3.3955					
*BnAMI40*	*cqOC-A9-16*	8.03551-12.88112	9.8381-13.4651					
*BnAMI64*	*cqOC-C5-3*	3.66296	3.3156					
*BnAMI65*	*cqOC-C5-4*	3.07706	4.1087					
*BnAMI67*	*cqOC-C6-2*	2.61268-5.22536	2.7008-5.5518					
*BnAMI68*	*cqOC-C6-3*	2.76241	5.7748					

LOD indicates the significance of the QTL interval and R^2^ indicates the contribution of the interval to the trait. Consensus QTL* were obtained from Wang et al. research (2013) and Consensus QTL** were obtained from Zhao et al. research (2016).

## Discussion

### Structural characteristics of the *BnTAR* family

The *TAA1/TAR* gene family is a class of pyridoxal-5’-phosphate-dependent amino acid transaminases, which can catalyze the conversion of tryptophan into Indole-3-Pyruvate (Zhao, 2012). *TAA1/TAR* gene family has been found in many crops, such as rice and maize (Phillips et al., 2011; Kakei et al., 2017). However, the genome wide identification, expression pattern and its relationship with oil content and seed weight of the *TAA1/TAR* genes have not been reported in *B. napus*. In this study, 102 *TAA1/TAR* family genes were identified in *B. napus* by using the Darmor as reference genome (Chalhoub et al., 2014). At the same time, we also searched the corresponding *BnTAR* genes in ZS11 genome of *B. napus* (Liu et al., 2021), and 130 genes were found. However, some difference was found between the two genomes cannot be matched. For example, four *BnTAR* genes that corresponding to AT5G53970 were found in Darmor genome, while only two *BnTAR* genes were found in ZS11 genome. On the contrary, two *BnTAR* genes that corresponding to AT5G53970 were found in Darmor genome. Those differences might be due to the different genetic backgrounds of Darmor and ZS11.

Introns increase the length of transcripts and are prone to detrimental effects on gene expression during their splicing (Jeffares et al., 2008). Meanwhile, genes that respond to stress are contained fewer introns (Lan et al., 2013). In this study, *BnTAR* genes were found to have a large and variable number of introns, with only nine genes having fewer than three and a maximum of 14 (**Figure S2**). Perhaps it is because *TAR*s genes, as an important enzyme in the auxin synthesis pathway that is the basis of plant life, maintain high expression and have more introns number throughout the whole plant life cycle. Meanwhile, *BnTAR* genes are very long (all but *BnALL5* are >1000 bp), and genes length are two or three times longer than that of CDS length.

Motifs are evolutionarily conserved amino acid/nucleotide sequence regions that usually play an important role in structure and function (Semwal et al., 2022). Many amino acid transaminase genes have been found in Arabidopsis genome, and most of these amino acid transaminases contain similar motifs, such as Pfam aminotransferase I/II signature (Pfam 00155), Pfam alliinase signature (Pfam 04864), Pfam aminotransferase III signature (Pfam 00202), etc. (Liepman and Olsen, 2004). The type and number of motifs varied in each branch, but the conserved motifs of Pfam 00155 or Pfam 04864 could be observed in each member of the *BnTAR* gene family. The differences in the motifs between *BnALL*s and *BnAMI*s were likely due to their configurations in the functional form. The three-dimensional structural analysis showed that the *BnTAR* proteins were all appeared to be overlapping bowl-shaped with a hollow in the center of the protein (**Figures 6B, D, F, H** **)**, which contains the pyridoxal binding site, moreover, the pyridoxal binding site of the *BnTAR* proteins contain ten amino acids, and the ninth binding site is very conserved as arginine, which is also a catalytic residue. In addition, *TAR*s proteins complete the amino acid transaminase function in dimer form, which could also be found in the aminotransaminase family in other species (Liepman et al., 2019).

### Duplication patterns and synteny analysis of the *BnTAR* family

Gene duplication not only expands genome content but also diversifies gene function to ensure optimal adapt-ability and evolution of plants (Xu et al., 2012). Tandem duplication, segmental duplication and whole-genome duplication (WGD) were the three mechanisms that could contribute to the gene family expansion (Xu et al., 2012). WGDs play a major role in Brassicas, particularly the mesopolyploidization events, which are simultaneously accompanied by extensive chromosomal and genetic diploidization processes (Hohmann et al., 2015). Many studies shown that Brassicaceae have experienced WGD events during their evolution (Rana et al., 2004; Parkin et al., 2005), nevertheless, little is known about the evolution and origin of the *TAA1*/*TAR* family in plants. *B. napus* was an allopolyploidy formed by the hybridization of *B. rapa* and *B. oleracea* in 7500 years ago (Chalhoub et al., 2014). In this study, it was revealed that the *BnTAR* family formation was associated with segmental duplications and WGD. The Arabidopsis genome contains 36 *TAR*s genes; therefore, more than 108 *TAR*s genes would be expected to produce through one WGT event in the *B. oleracea* or *B. rapa* genome, and finally leading to much more *TAR*s genes in *B. napus*. However, only 102 *TAR*s genes were observed in the *B. napus* genome in the present study. These results indicated that more than 50% of the duplicated *TAR*s genes were lost after WGT, which might be due to the extensive chromosome reshuffling during the diploidization after WGT (Cheng et al., 2014). It was found that some duplicated *TAR*s genes disappeared in the *B. napus* genome. For instance, certain genes that are homologous to Arabidopsis (*AT1G34060*, *AT1G01480*, *AT1G34040*, *AT2G22810*, *AT2G24850*, *AT2G24850*, *AT4G08040*, *AT5G49810*, *AT5G51690*, *AT1G23320*, *AT1G62960*, *AT1G77670*, *AT2G13810*, *AT3G61510*, *AT4G11280*, *AT4G24670*, *AT4G28410*, *AT5G36160*) had less than four copies in *B. napus* (**Table S3**), by simply calculating the Arabidopsis genes at three times after the WGD event, these Arabidopsis genes have thirty fewer homologous genes in *B. napus*, which indicates the thirty genes that were not detected might have been lost. Similar losses after WGT also can been observed in other gene families of Brassica (Yu et al., 2014).

Segmental duplication also plays an important role in the expansion of *BnTAR* family, and 71 *BnTAR* genes were determined to have one or two gene pairs in the corresponding regions. Therefore, the formation of 70% (71/102) of the *BnTAR* genes is related to segmental duplication. Synteny analysis demonstrated that most *BnTAR* gene family members are located in well-conserved synteny regions, and some genes were deleted or gained. Similarly, synteny genomic regions with some deleted genes have been identified in other gene families (Yu et al., 2014). Despite the loss of some genes after WGT, the present findings suggested that segmental replication and WGD might play an important role in the expansion of the *TAR*s family in *B. napus*, while tandem replication only played a minor role. The characteristics of the *BnTAR* family duplication patterns and synteny analysis were consistent with the Brassicaceae evolutionary history.

The origin and evolution of auxin biosynthetic genes in plants have been controversial for a long time (Wang et al., 2014; Yue et al., 2014). However, the recent studies have found that auxin biosynthesis was mainly originated in charophycean green algae, which was most closely related to terrestrial plants (Romani, 2017). The present results of the phylogenetic, evolutionary, and structural analysis show that the *BnTAR*s have a close phylogenetic relationship and similar structure (**Figure 4**, **Figure S2, Table S3**). As discussed above, *BnTAR* family genes undergo WGD and segmental replication, which was also observed in *Oryza sativa* and *Triticum aestivum* (Shao et al., 2017; Guo et al., 2020). *BnAMI8* and *BnAMI54* that belonged to a relatively unique phylogenetic branch with a different structure from other *BnTAR*, which may be caused by some additional gene replication and fusion events. Such events may produce more diverse monomers that combine to form more diverse amino transaminases.

### The relationship between *BnTAR* genes and seed oil content and seed weight in *B. napus*

The expression patterns of *BnTAR* family genes are different in different tissues, it was found that about a third of the *BnTAR* genes were barely expressed in *B. napus*, further analysis showed that the expression of *BnTAR* genes were with two patterns, one was mainly expressed in vegetative organs (such as roots, stems and leaves), the other was mainly expressed in reproductive organs (such as flowers and seeds) (**Figure 7**, **Figure S4**). The similar *TAA1*/*TAR* genes expression patterns were also found in wheat and *A. thaliana* (Poulet and Kriechbaumer, 2017; Shao et al., 2017).

In recent studies, more attention has been paid to the effects of these genes on the formation of lateral roots (He et al., 2011; Zhou et al., 2011; Ma et al., 2014), while the effect of *TAR* gene on the yield of economic crops was ignored, and the role of the *TAR* gene in yield increase is only studied in wheat (Shao et al., 2017). By compared the transcriptomic results of high and low oil content samples, and the relatively high and low seed weight samples, a preliminary study on the effect of *BnTAR* genes on seed traits in *B. napus* was conducted. We found that the expression of most *BnTAR* genes was not significantly different between high and low oil samples and the relatively high and low seed weight samples, but there were still several genes, such as *BnALL2*, *BnALL7*, *BnAMI27* and *BnAMI67*, which were higher in the late stage of seed development in high oil samples, higher in the middle stage of seed development and lower in the late stage of seed development in high seed weight samples (**Figure 8**), it suggested their effects on seed oil content and weight regulation.

QTL mapping is an effective tool for the analysis of the genetic mechanism of complex quantitative traits (Mauricio, 2001), and a number of QTLs for oil content have been detected in diverse crops, for instance, *Zea mays* (Mangolin et al., 2004; Zhang J. et al., 2008; Yang et al., 2010), *Glycine max* (Orf et al., 1999; Kabelka et al., 2004; Zhang et al., 2004), *Arachis hypogaea* (Sarvamangala et al., 2011; Pandey et al., 2014) and *B. napus* (Gu ]et al., 2016; Chao et al., 2022). On the basis of the results of the previous quantitative trait locus (QTL) results for oil content and seed weight in our group, we found that the 21 and 12 *BnTAR* genes were located in the QTL interval of oil content and seed weight, respectively (**Table 2**). And three major QTLs (*cqOC-A9-9*, *cqOC-C5-3* and *cqOC-C5-4*), where *BnAMI35*, *BnAMI64* and *BnAMI65* located were showed a large effect (phenotypic variation>10%) in at least two trials (Chao et al., 2017). Also, some *BnTAR* genes, for example, *BnALL2*, *BnALL3*, *BnAMI10*, and *BnAMI67*, had not only been mapped in QTL intervals, but also affected seed oil content in previous transcriptome analysis. These results indicated that some of the *BnTAR* genes play an important role in regulating the seed weight and seed oil formation in *B. napus*.

## Conclusion

In this study, 102 *BnTAR* genes were identified and comprehensively analyzed. A conserved catalytic structural domain of the *TAR* superfamily was observed, and WGD and segmental replication were the main events for the *BnTAR* gene family formation. Expression patterns combined with QTL analysis provided the new insights into the biological functions of *BnTAR* proteins for oil content and seed weight.

## Data availability statement

The original contributions presented in the study are included in the article/**Supplementary Material**. Further inquiries can be directed to the corresponding author.

## Author contributions

XC and XL did the identification of *TAR* family genes in *B. napus*, duplication pattern analysis, and drafted the manuscript. XL did multiple sequence alignments of the *BnTAR* genes and conducted the phylogenetic analysis. XC, XL, JH, and MT did the RT-qPCR experiment. XC, XL, and HL analyzed QTL and transcriptomic data. XC and XL collected *TAR* genes information of other species, and did the structural analysis and prepared **Figures 1**–**8** and the **Supplementary Information**. ML designed and supervised the experiment. ML and JH revised the manuscript. All authors reviewed the manuscript.
