# Supplementary material for: The genome wide analysis of Tryptophan Aminotransferase Related gene family, and their relationship with related agronomic traits in Brassica napus
Source: Front Plant Sci. 2022 Dec 21;13:1098820. doi: 10.3389/fpls.2022.1098820 (PMC9811149; doi:10.3389/fpls.2022.1098820)
Supplement: Supplementary file 1 [file DataSheet_1.docx]

**Supplementary Information**

**Figure S1. Alignment of *BnTAR* protein sequences in each family or branch**


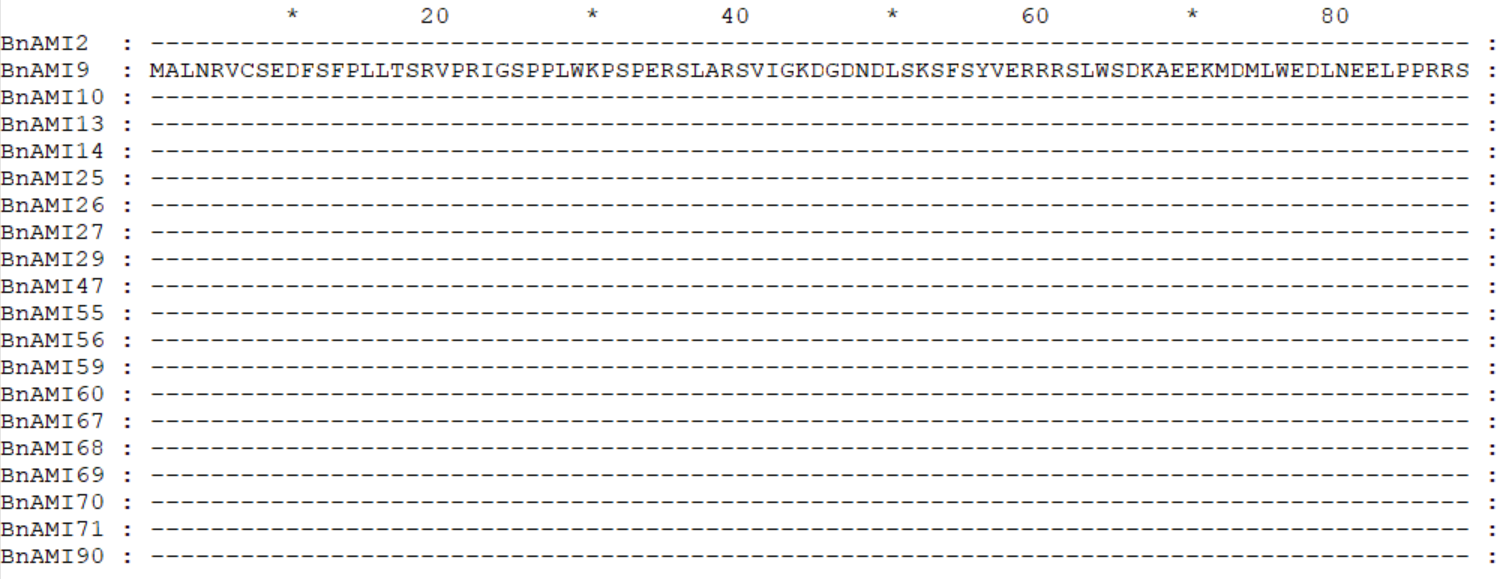


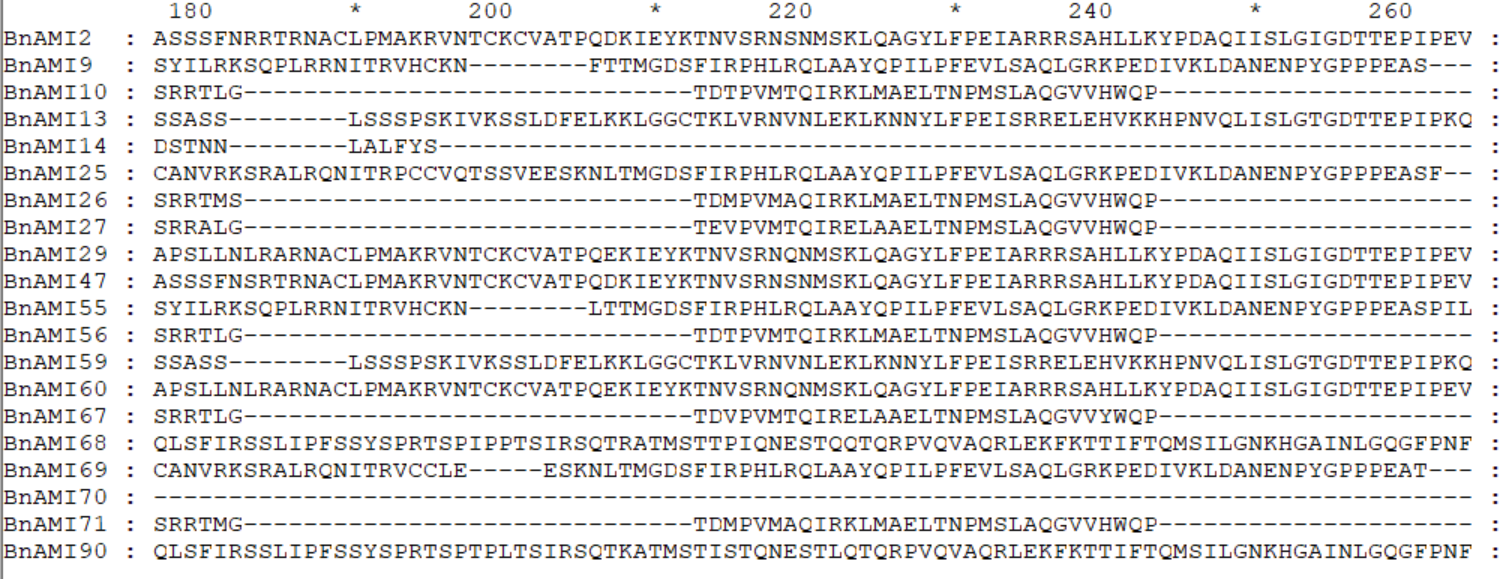


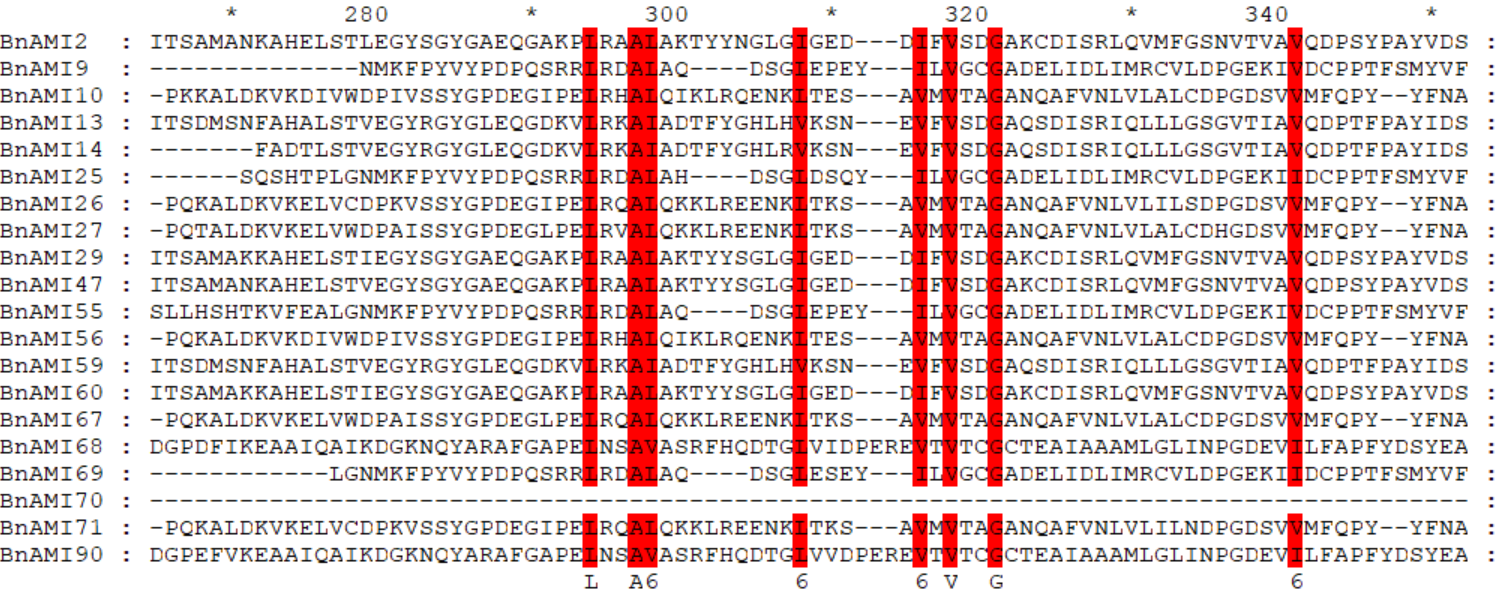


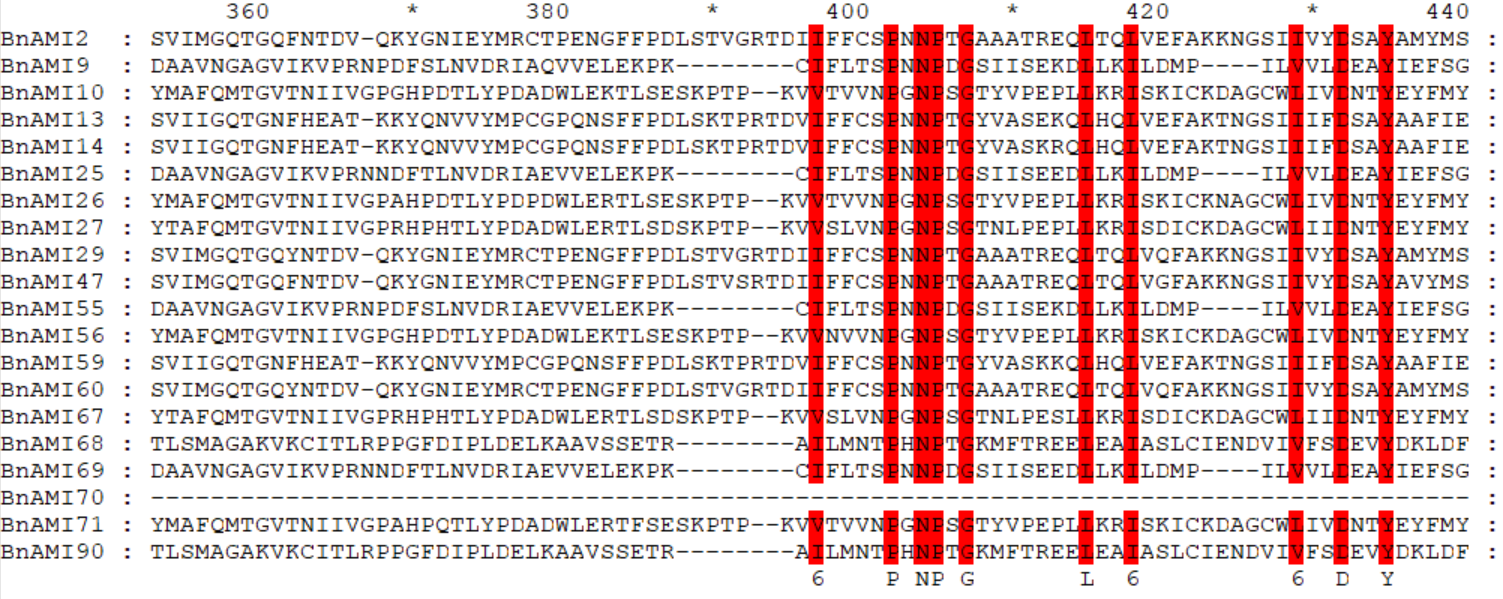


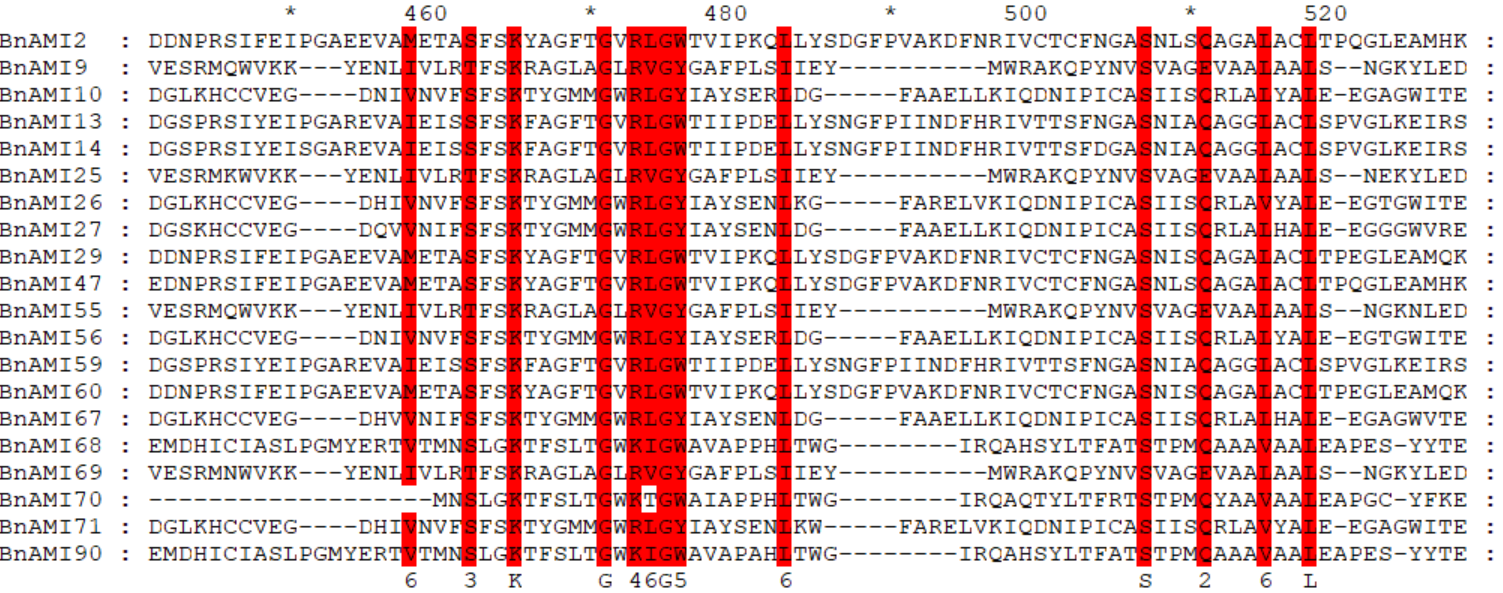


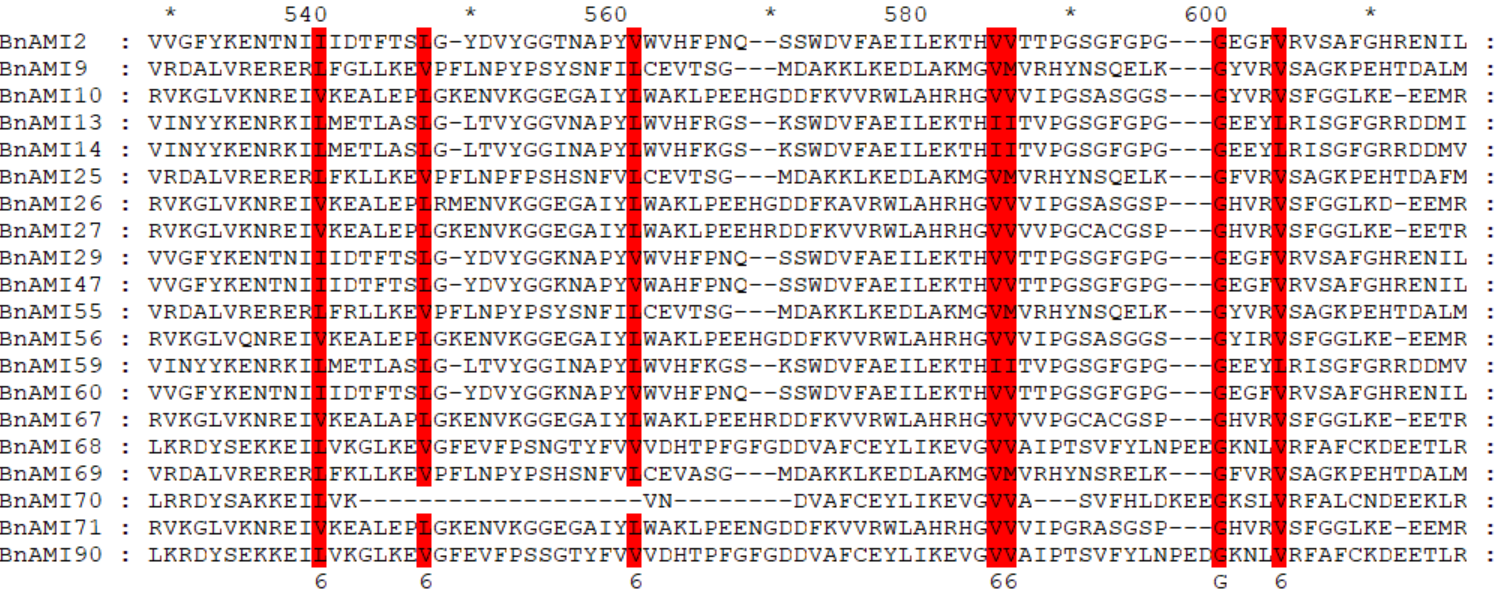


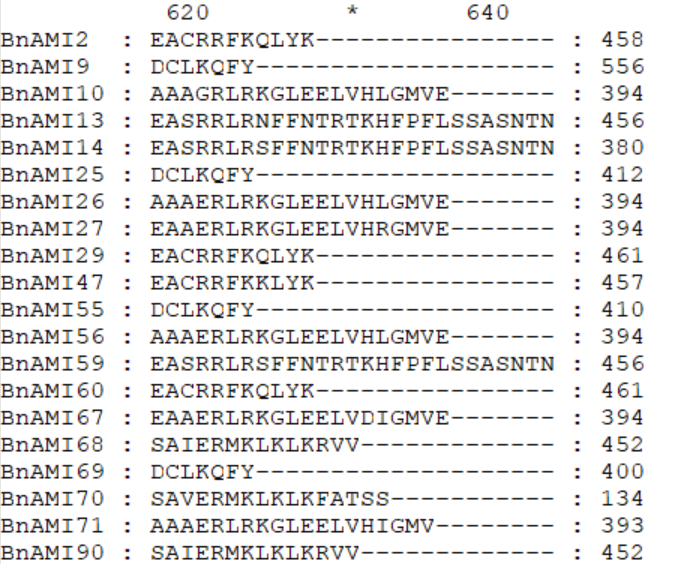


*BnTAR* proteins of the first branch of *BnAMI* family containing *AMI2* and *AMI69*.（conservative property>90）


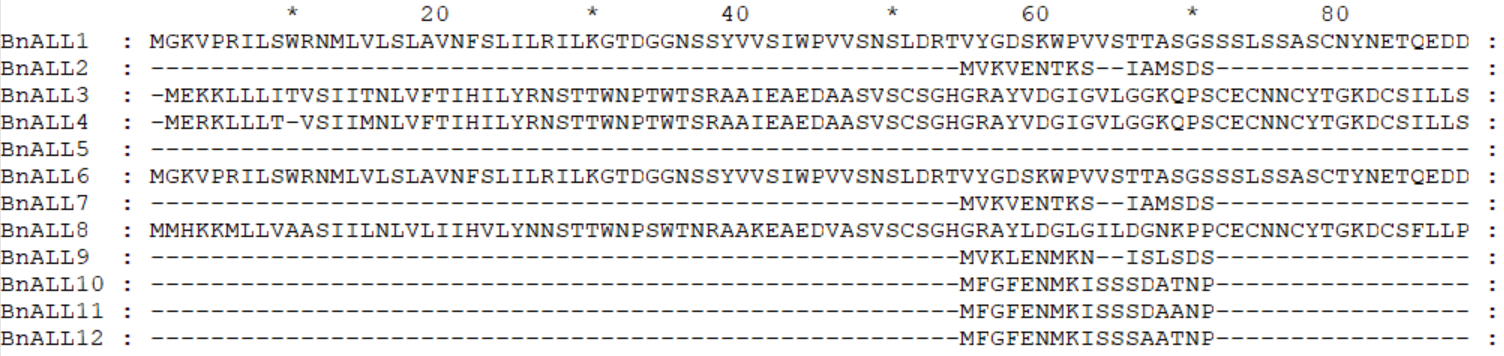


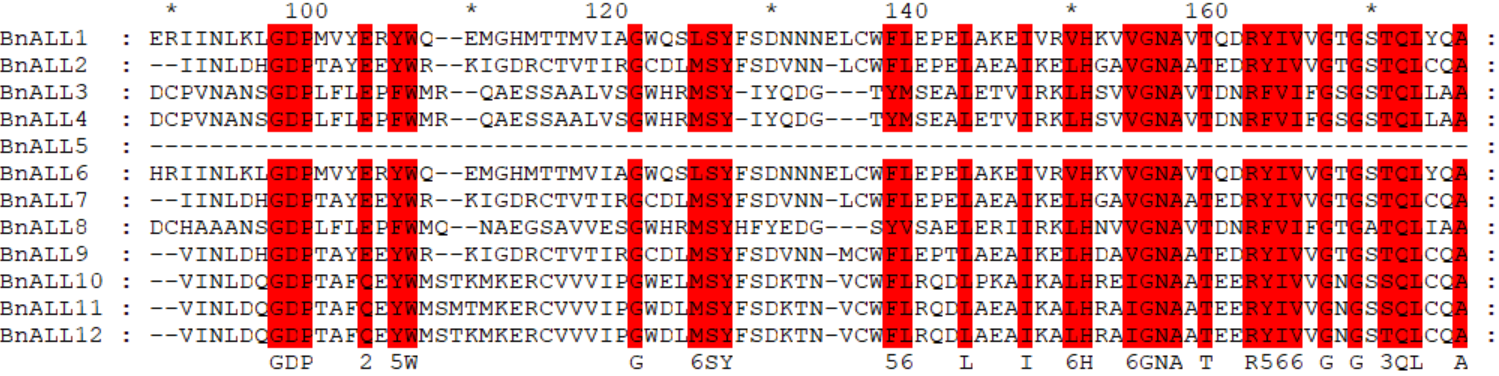


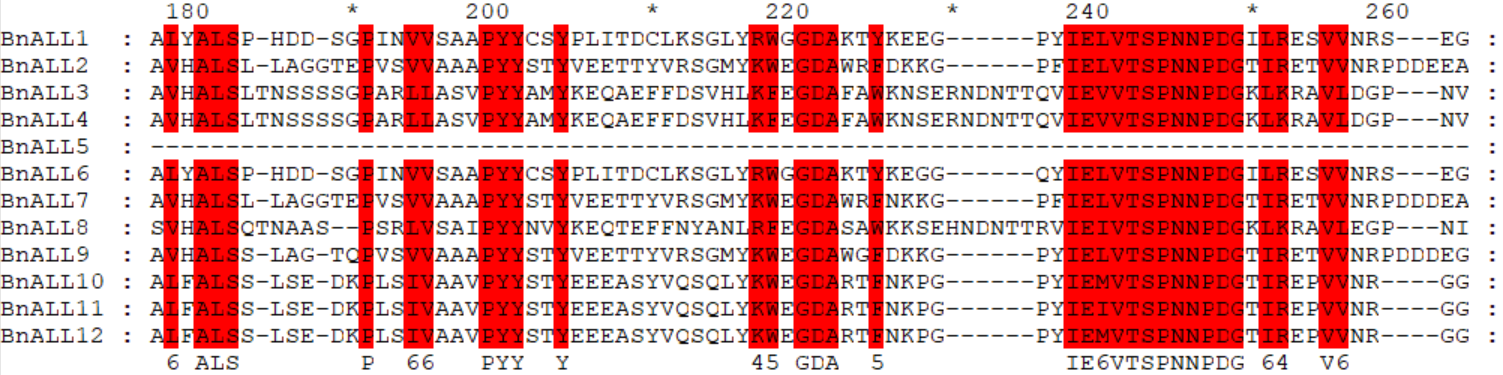


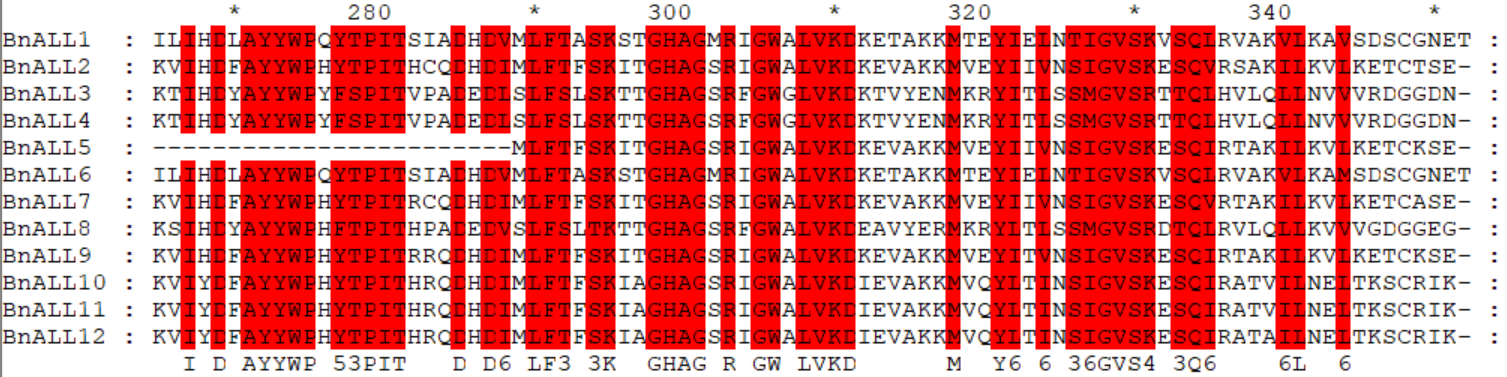


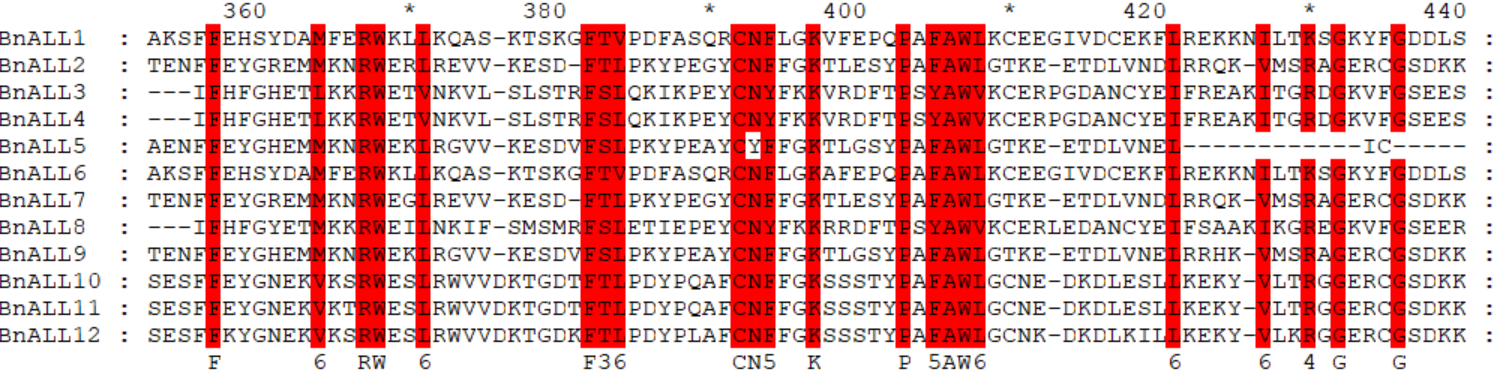


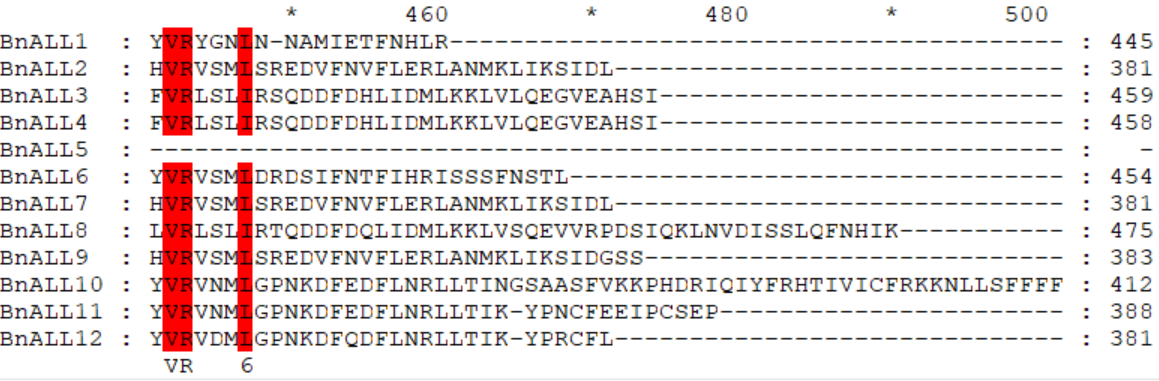


*BnTAR* proteins of *BnALL* family（conservative property>90）


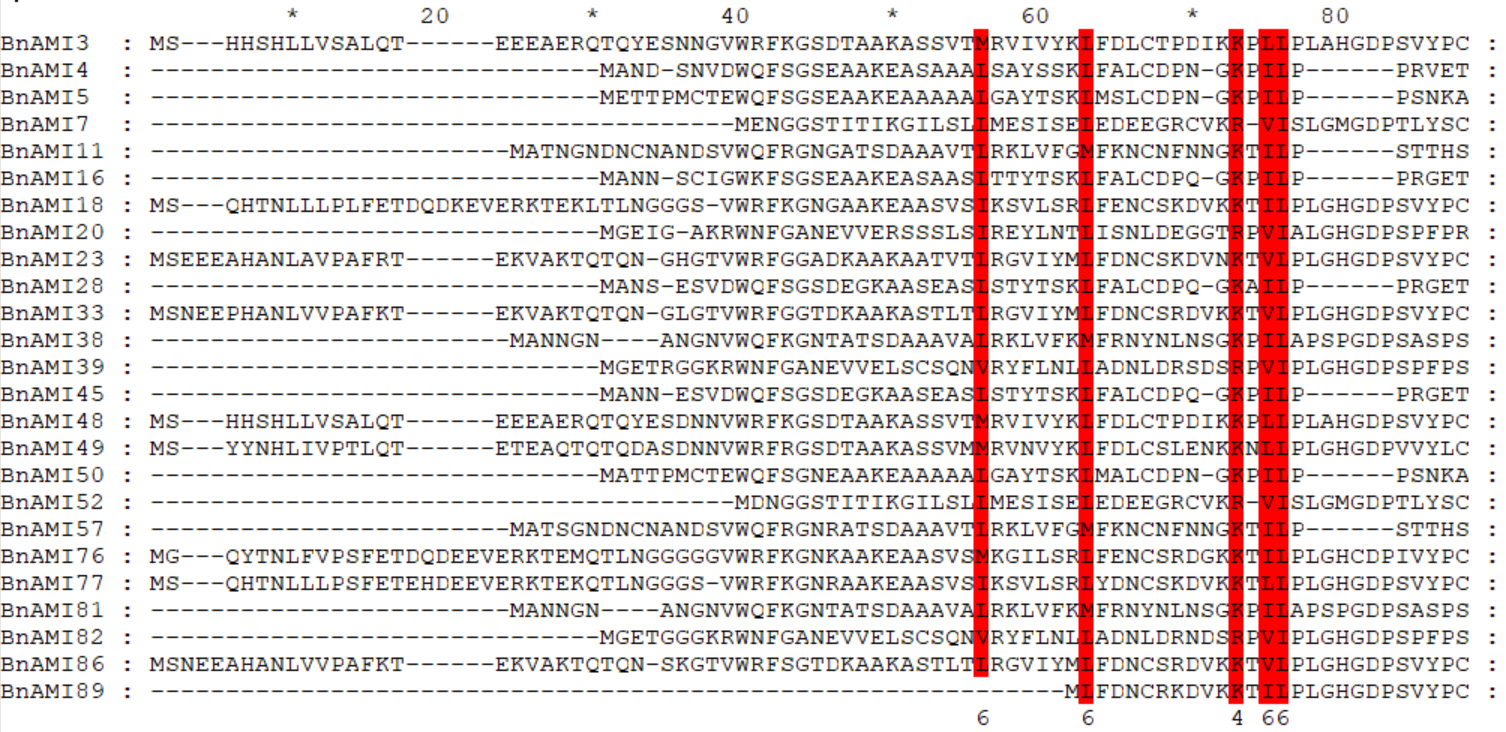


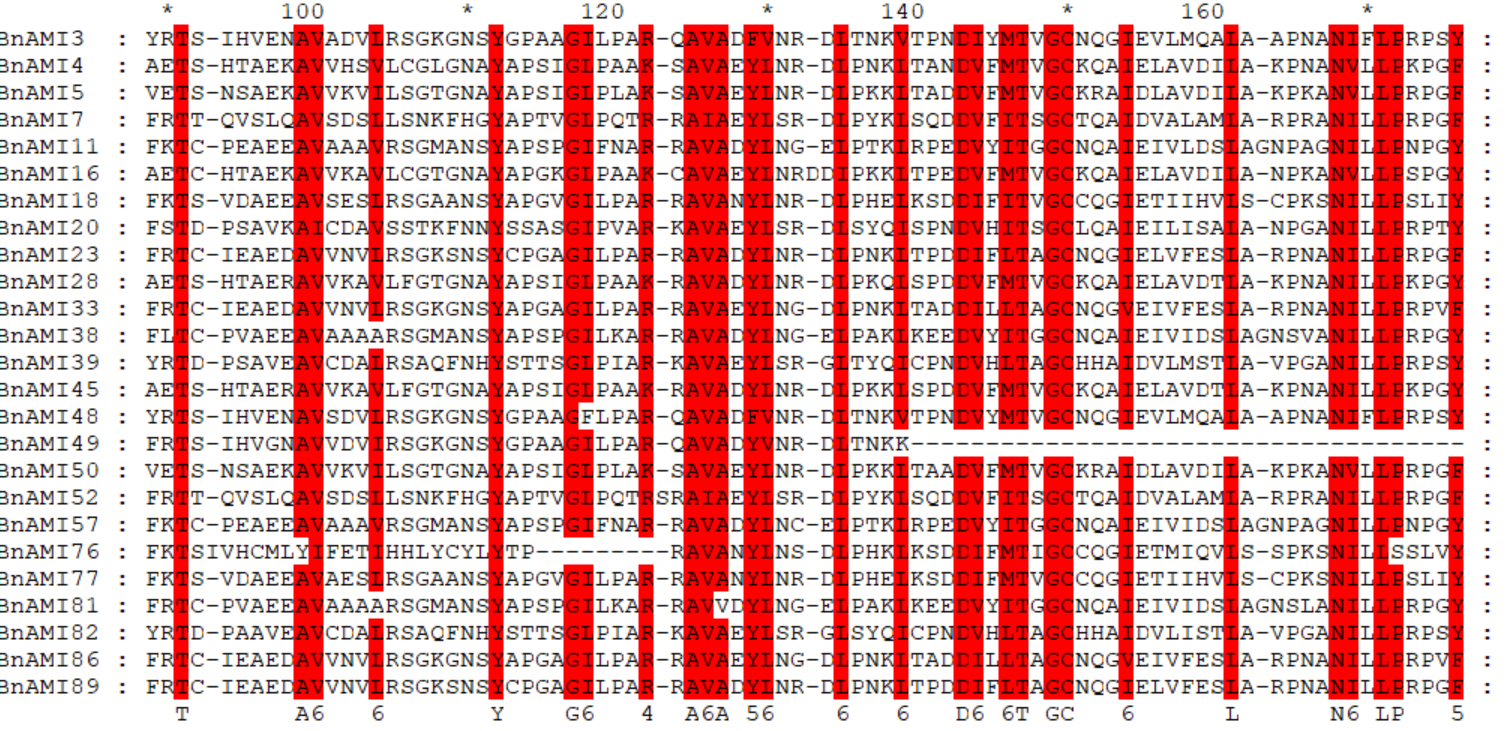


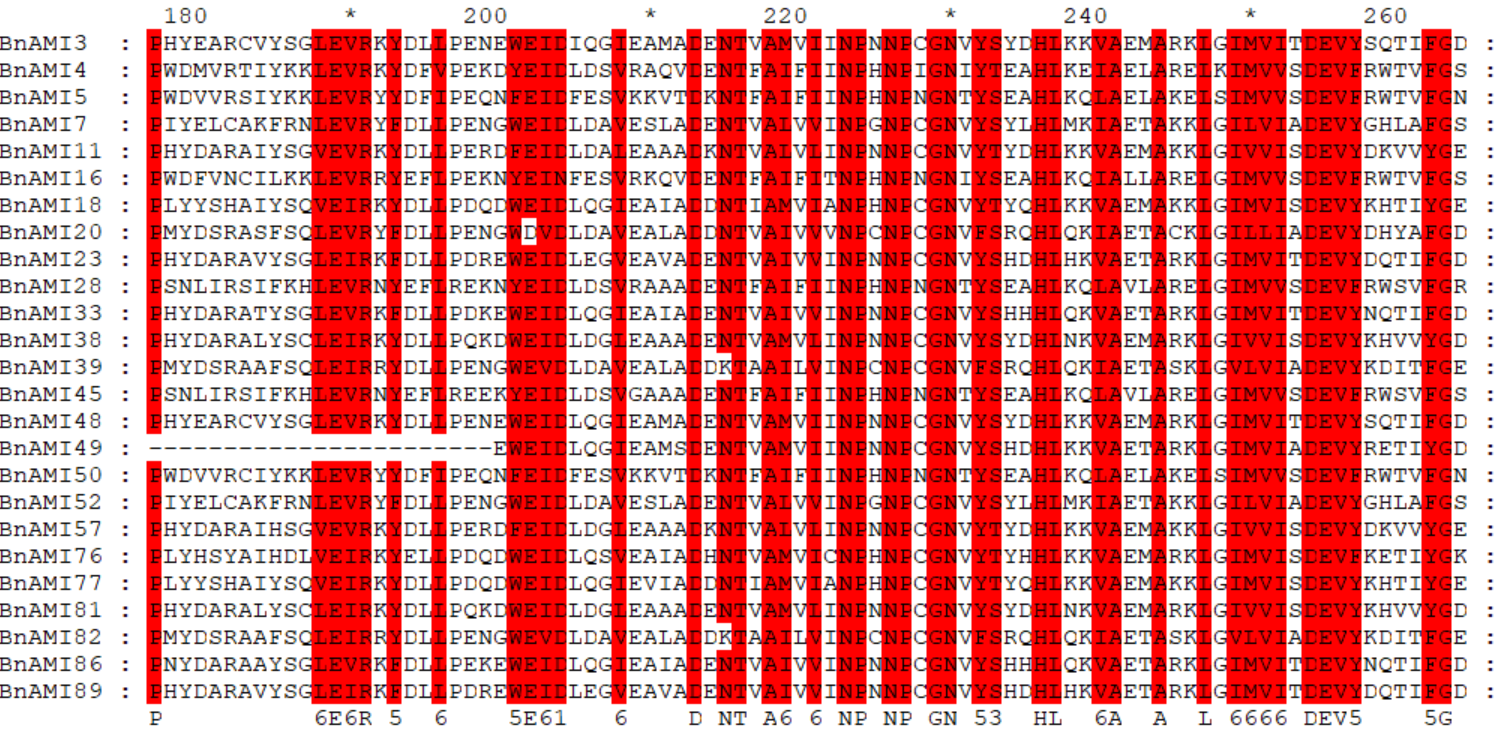


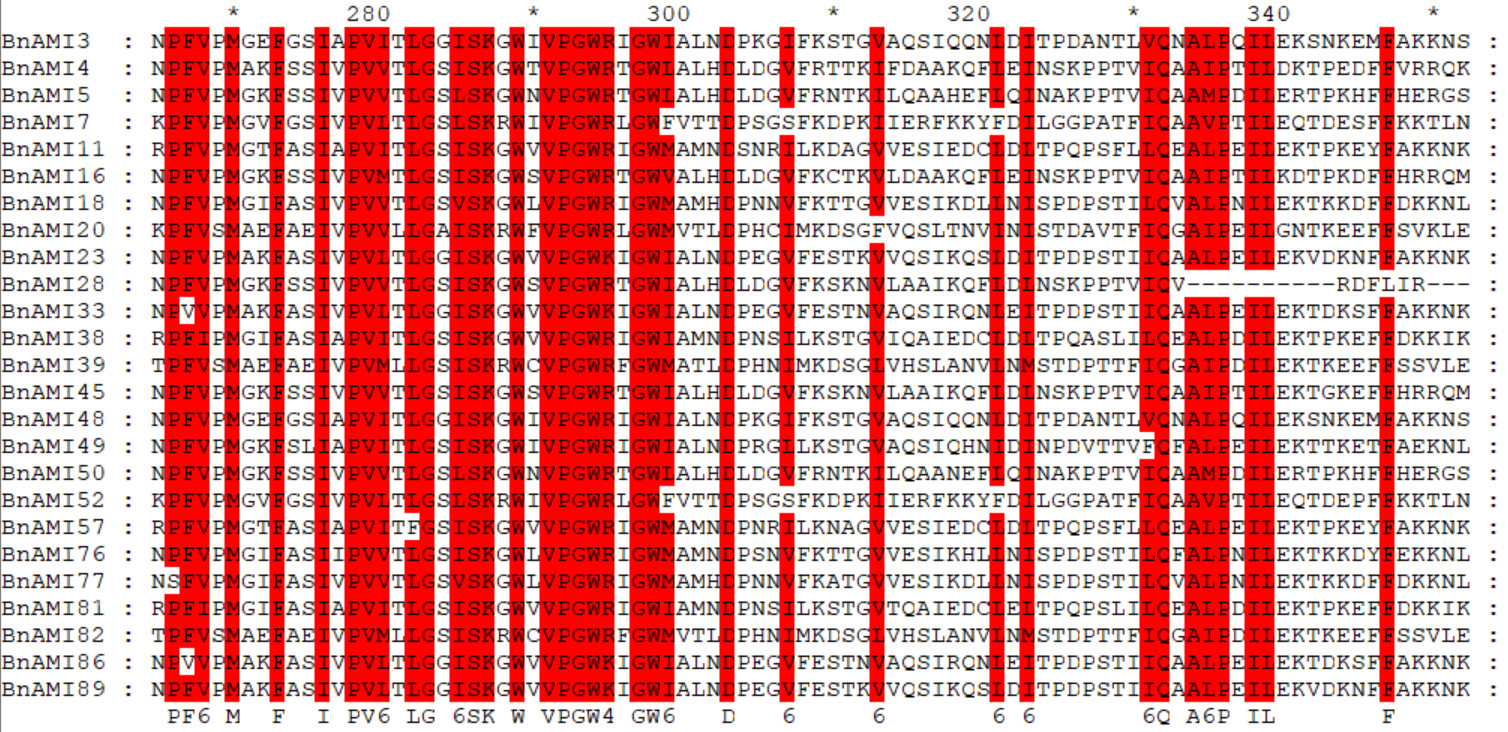


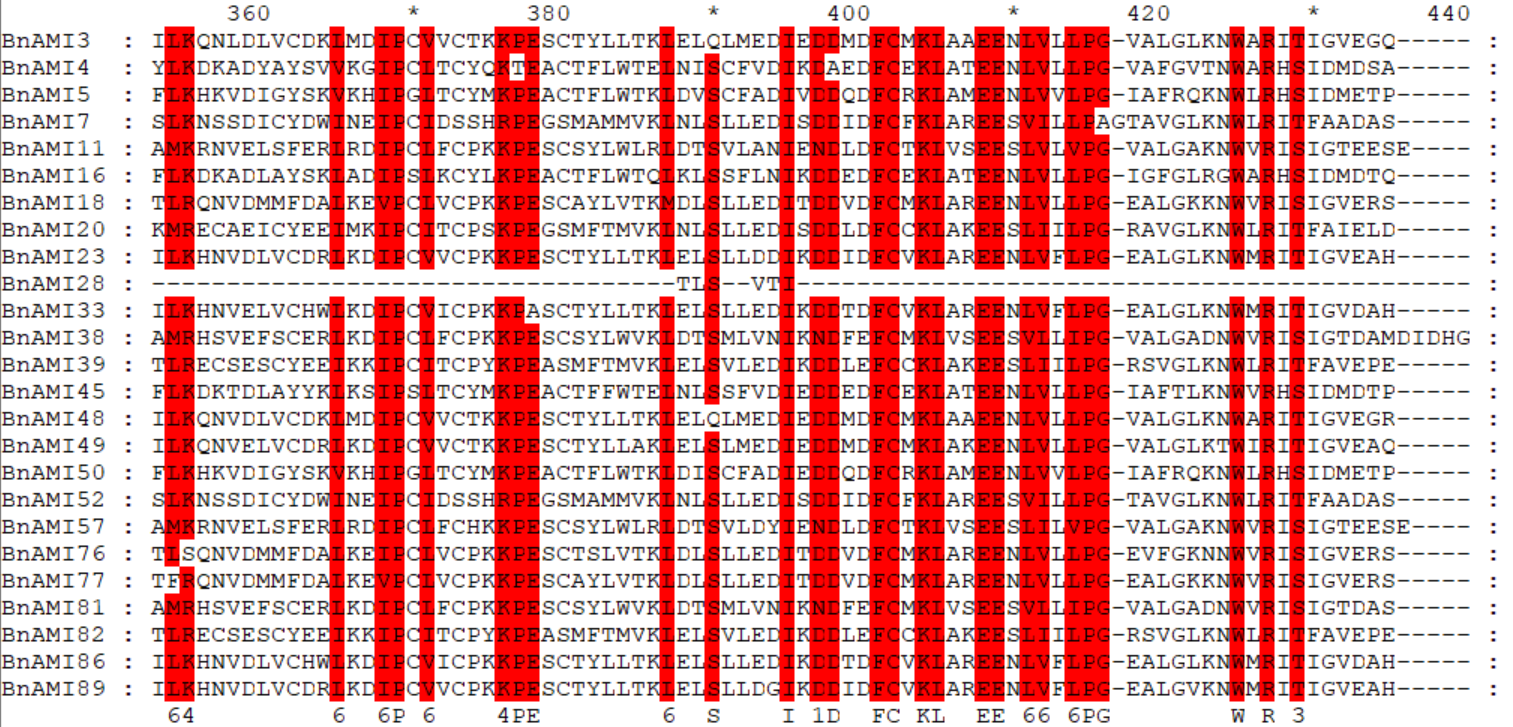


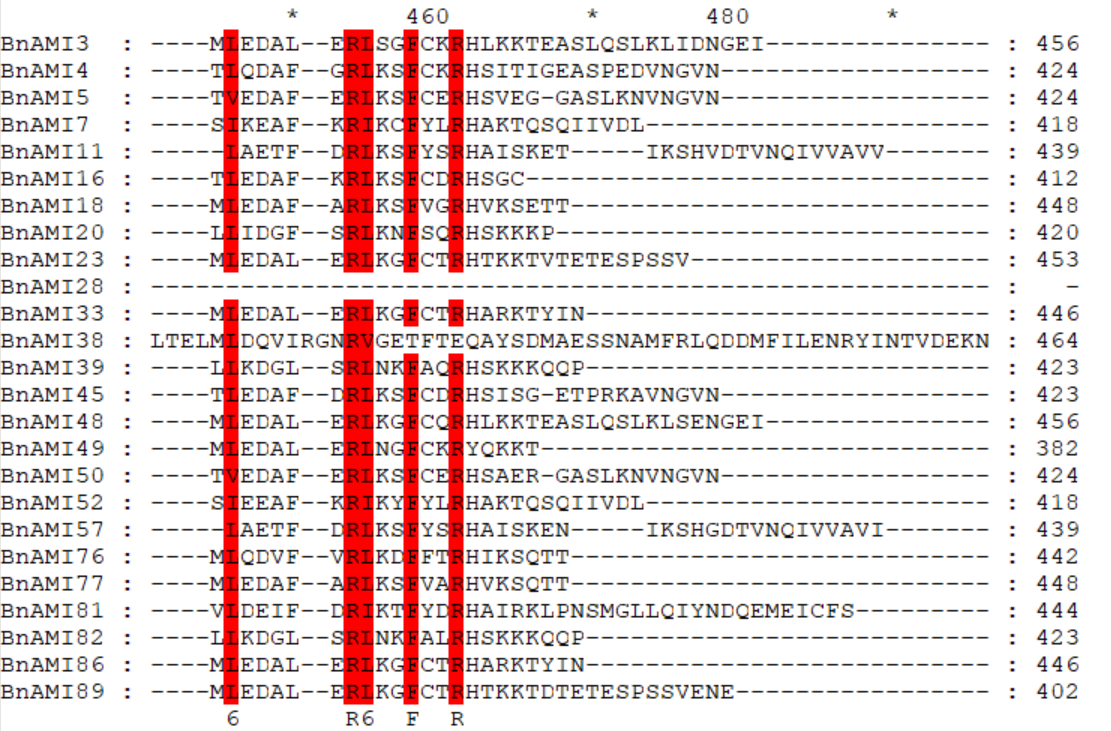


*BnTAR* proteins of the second branch of *BnAMI* family containing *AMI39* and *AMI86*.（conservative property>90）


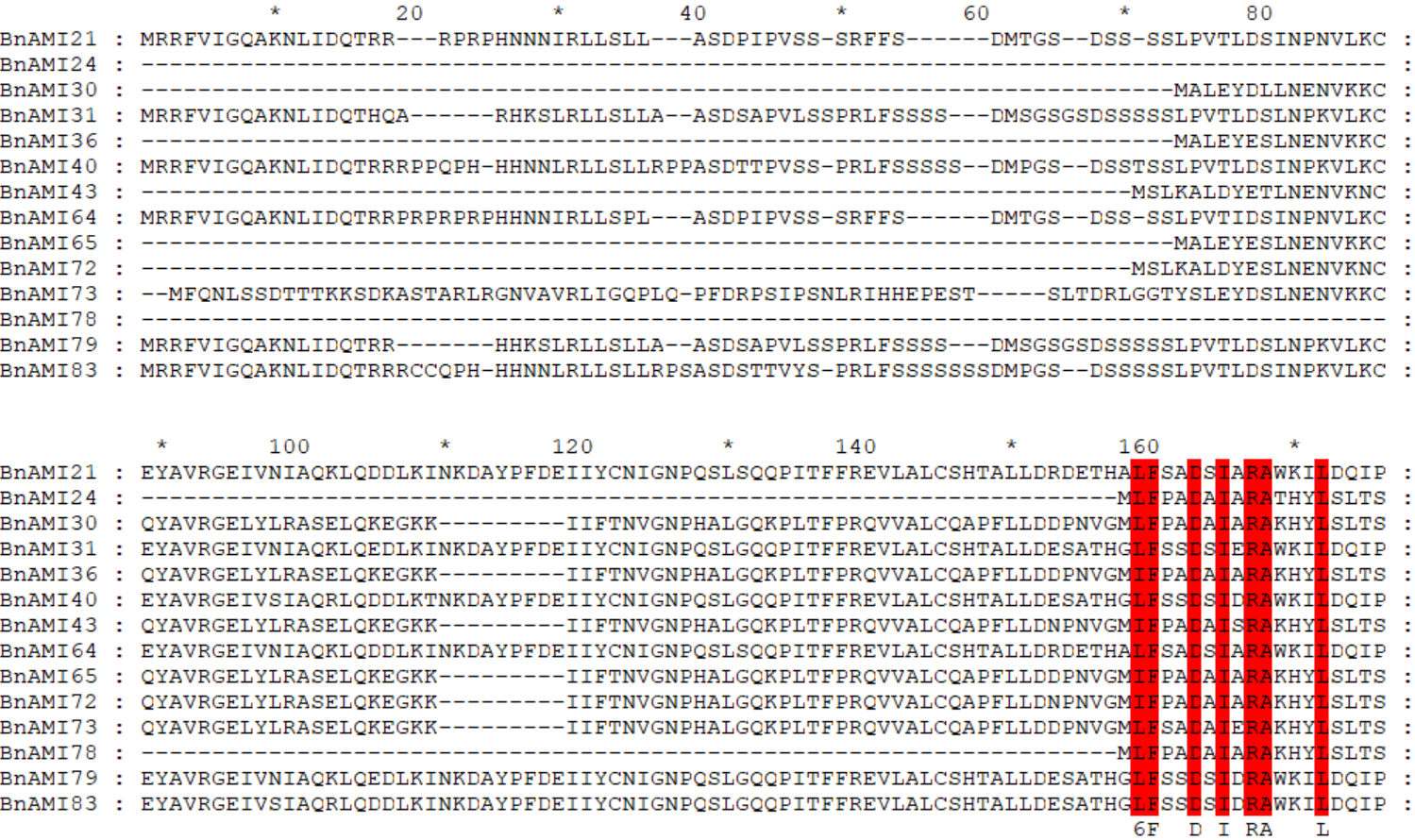


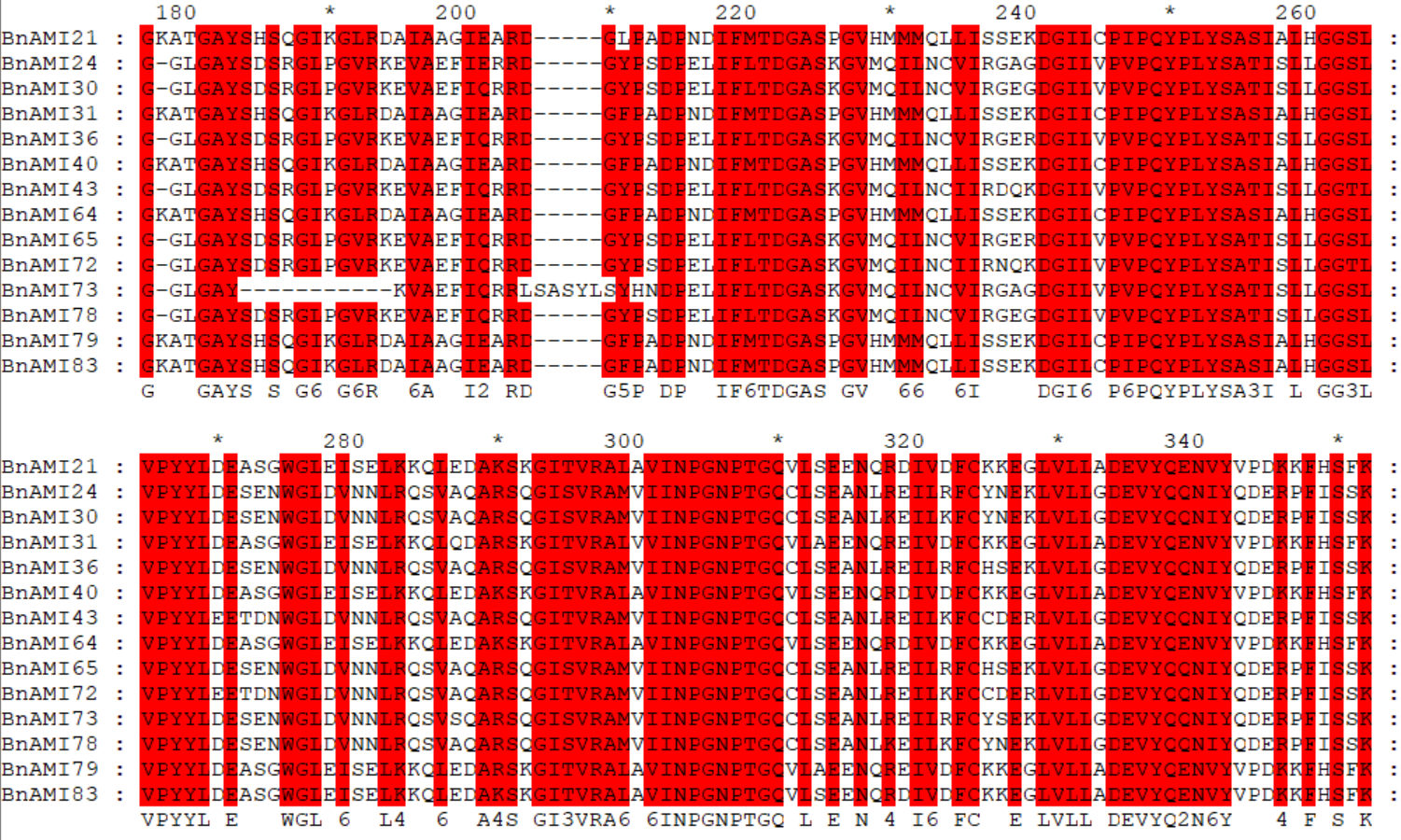


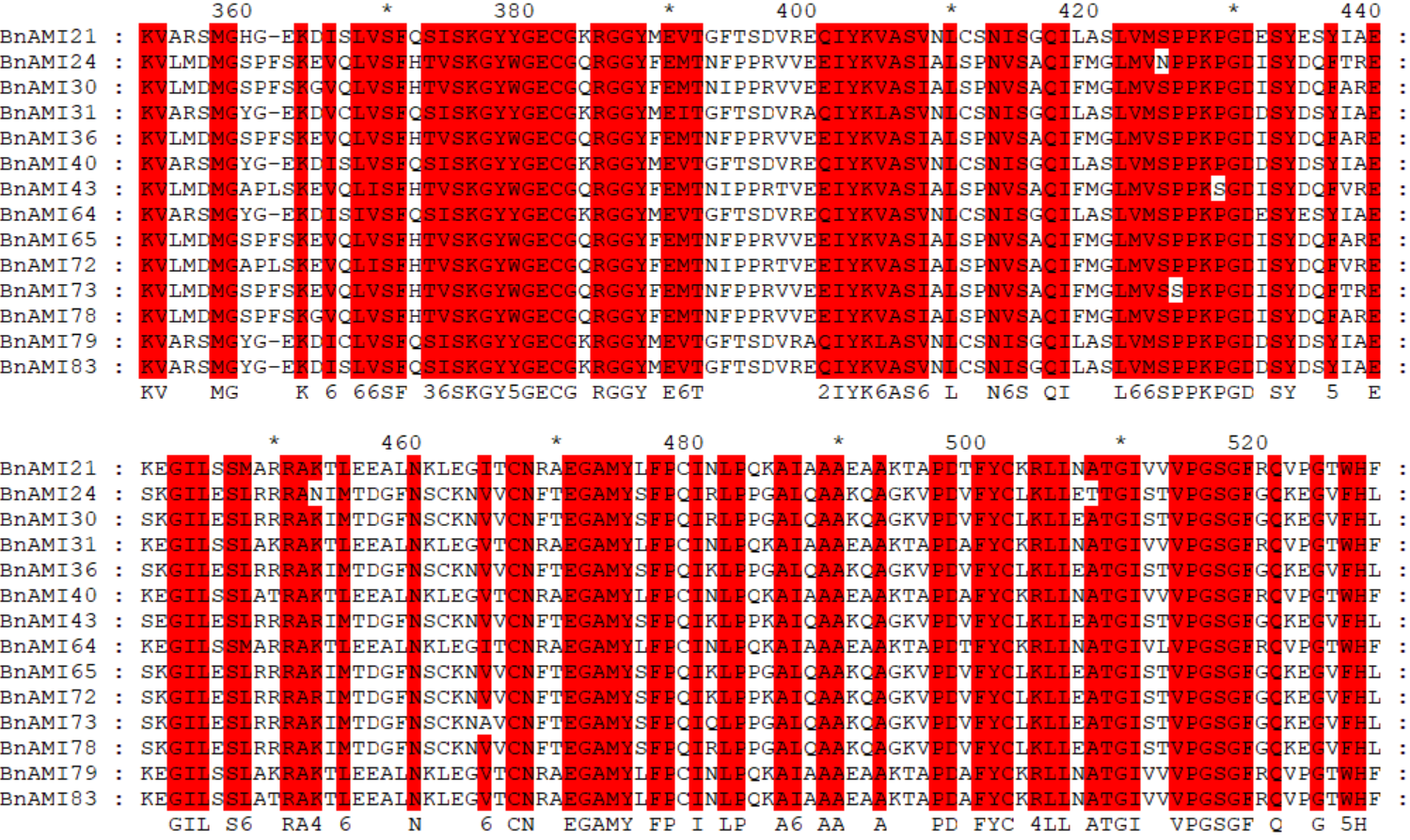

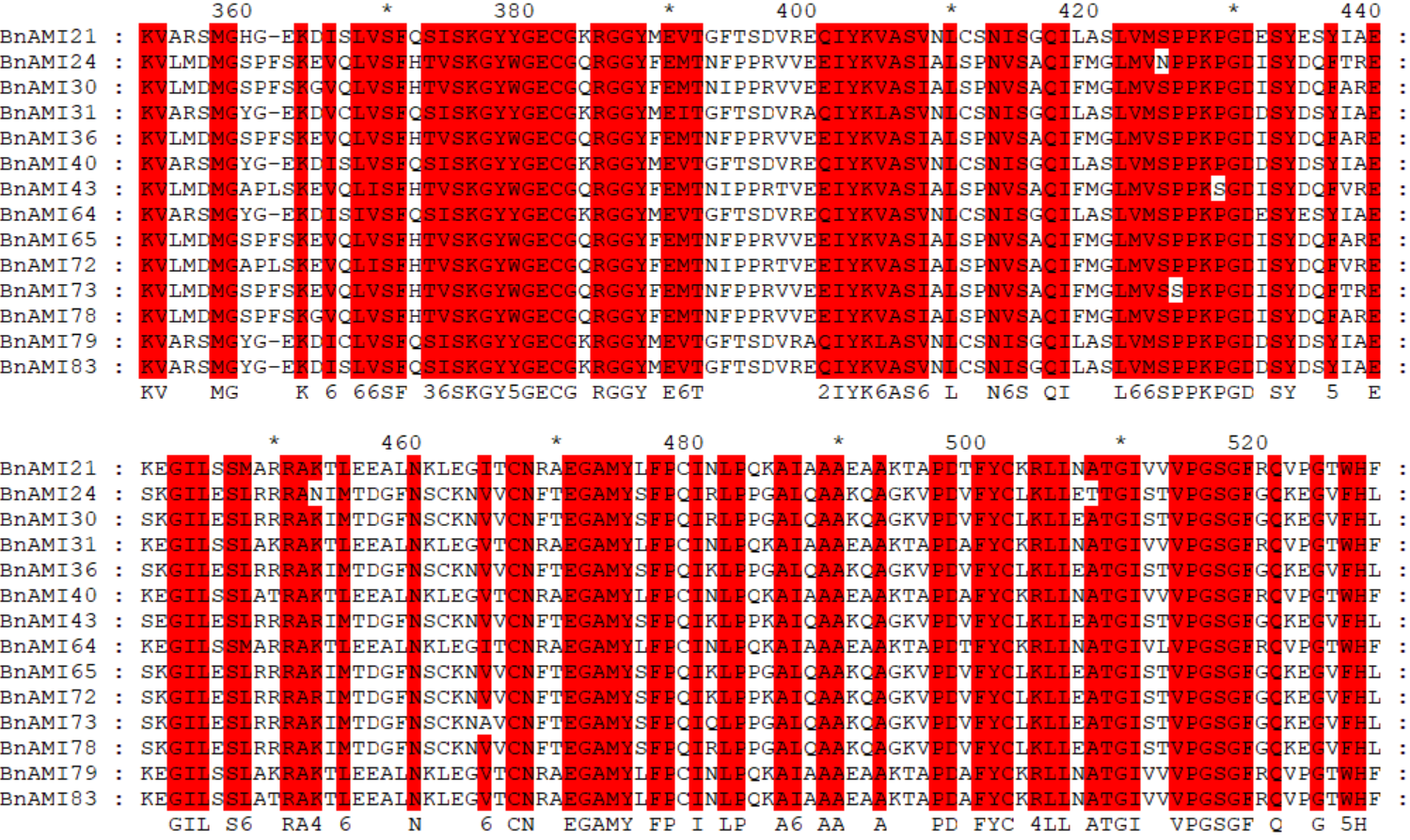


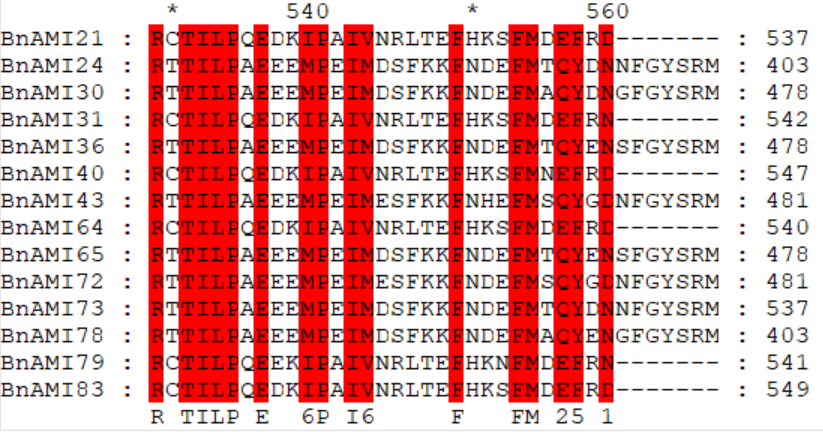


*BnTAR* proteins of the third branch of *BnAMI* family containing *AMI31* and *AMI65*.（conservative property>90）


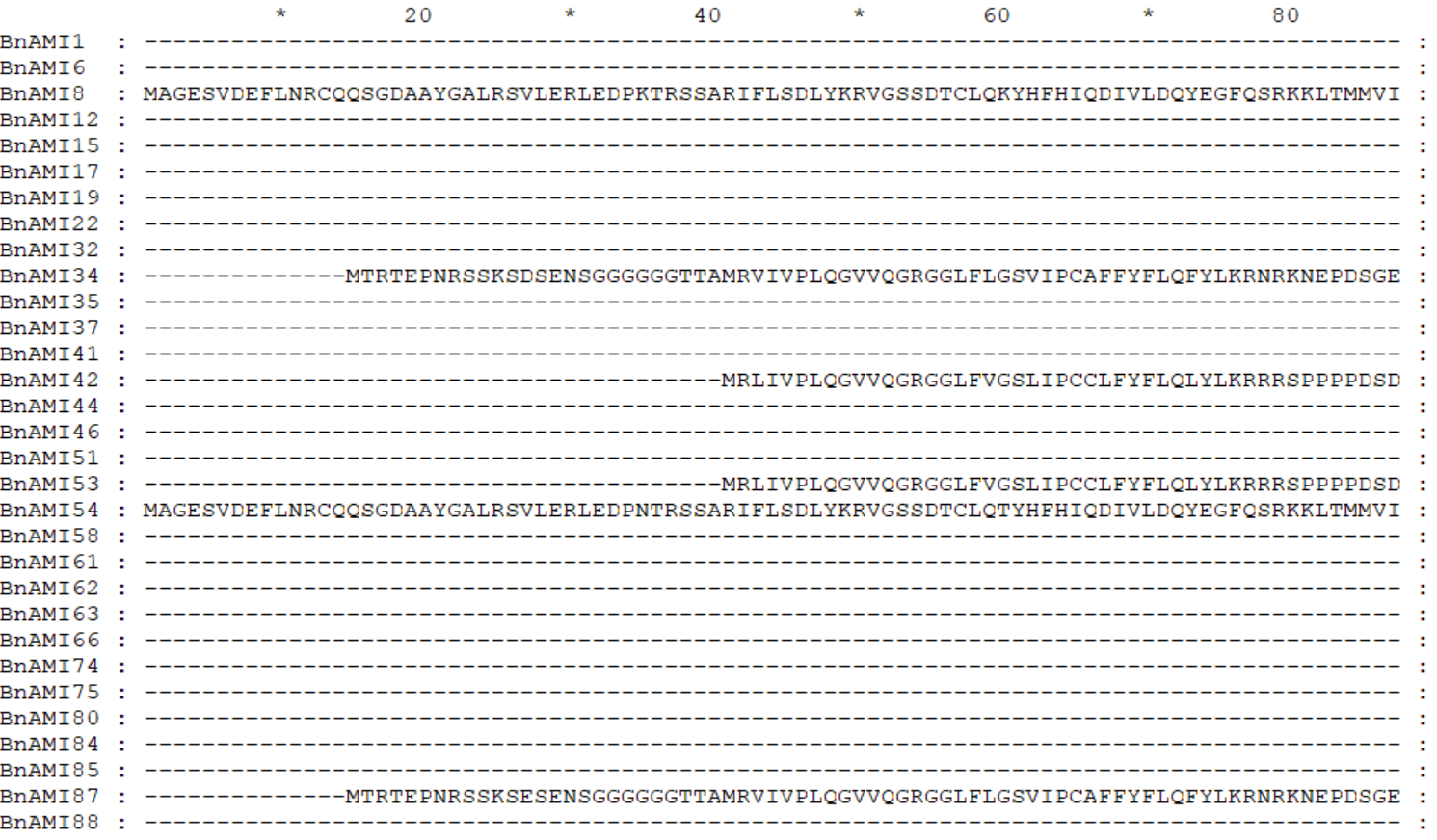


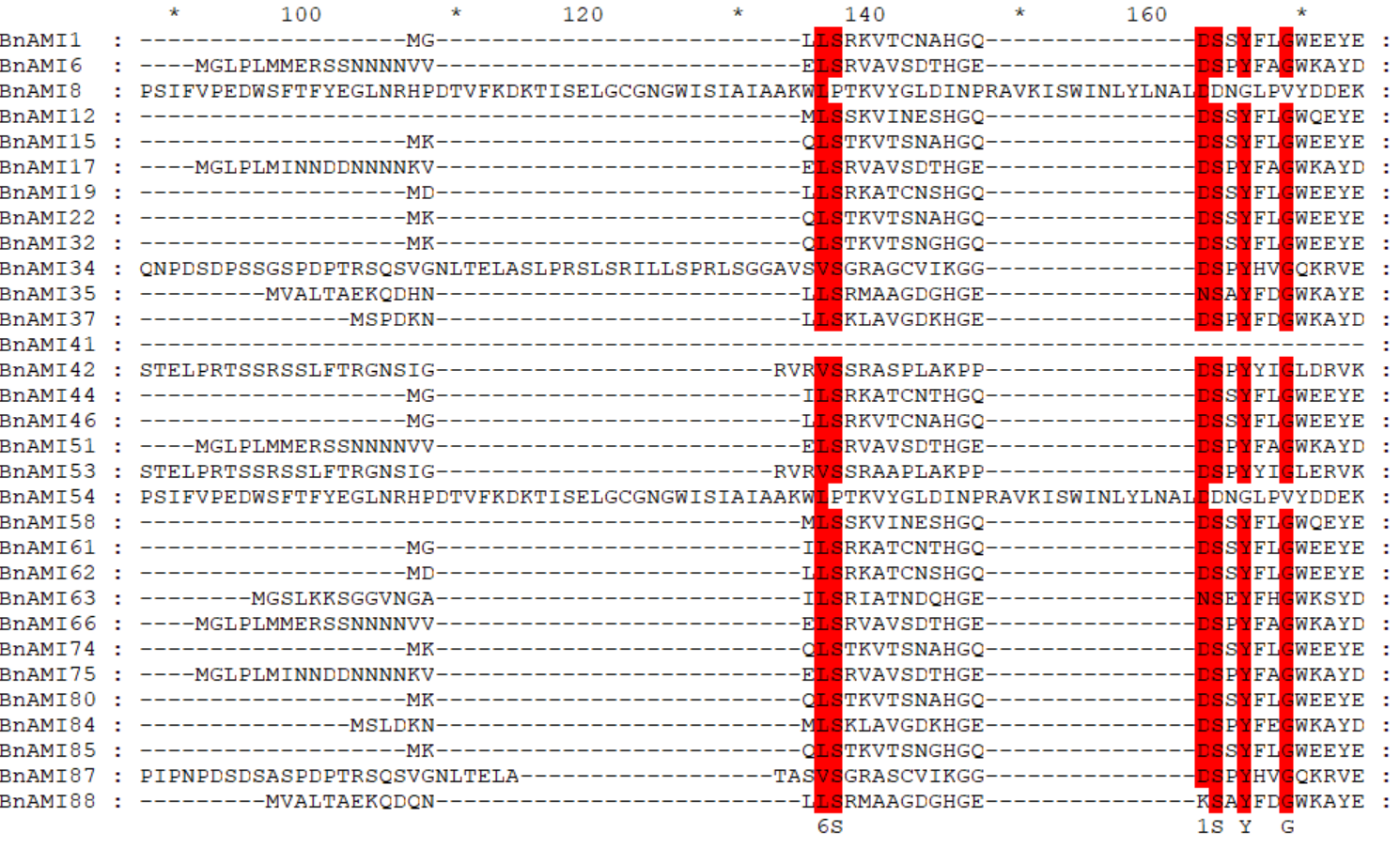


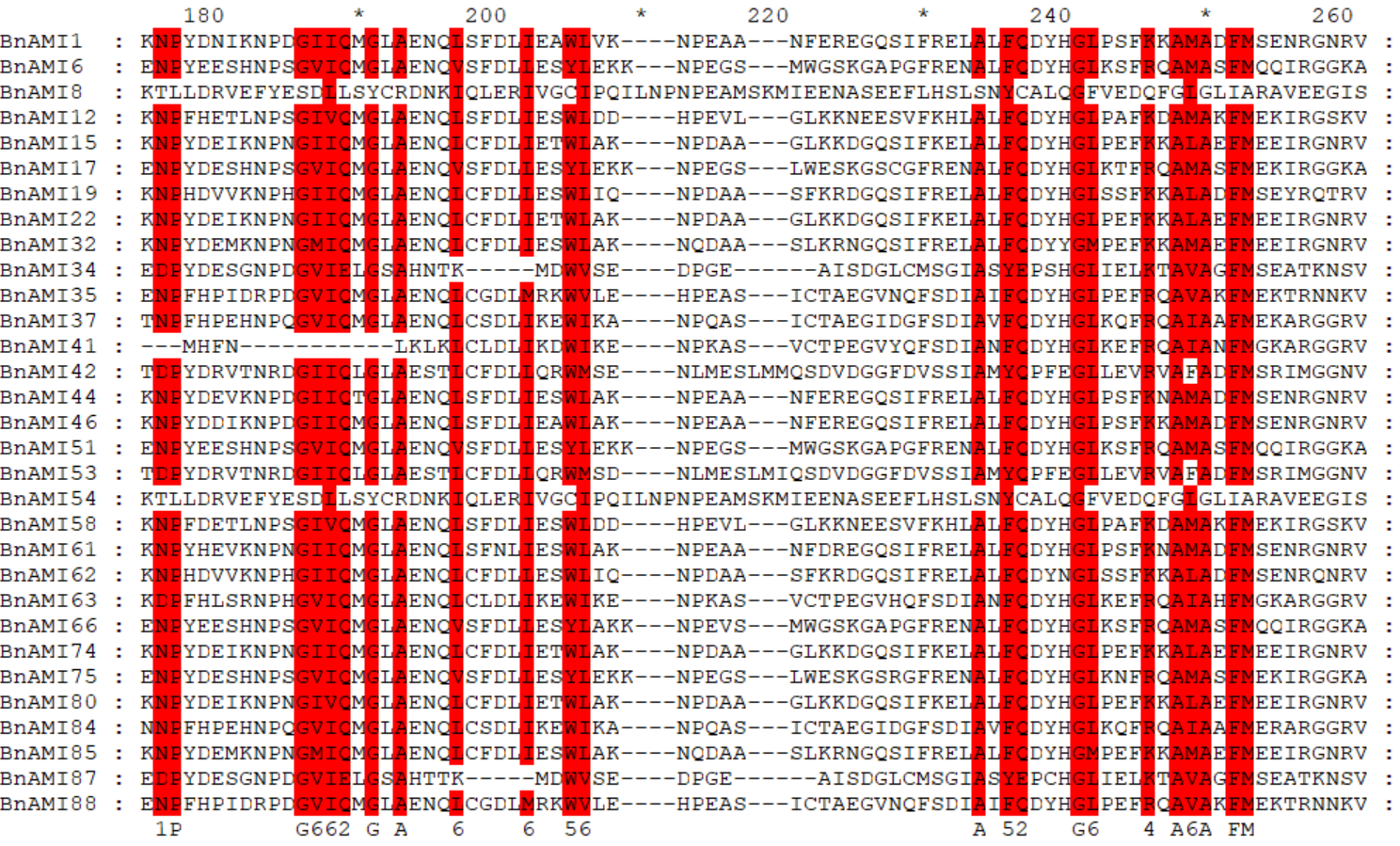


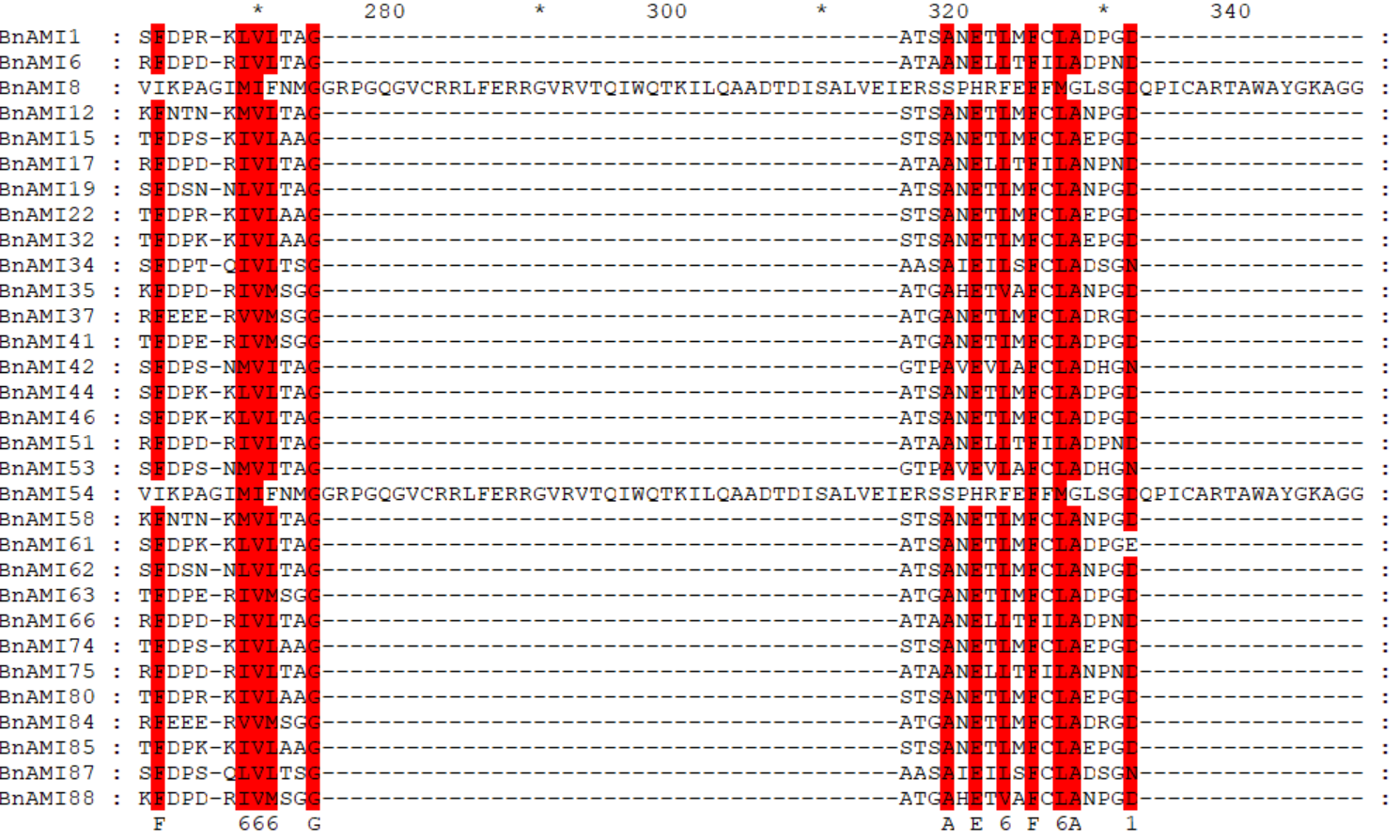


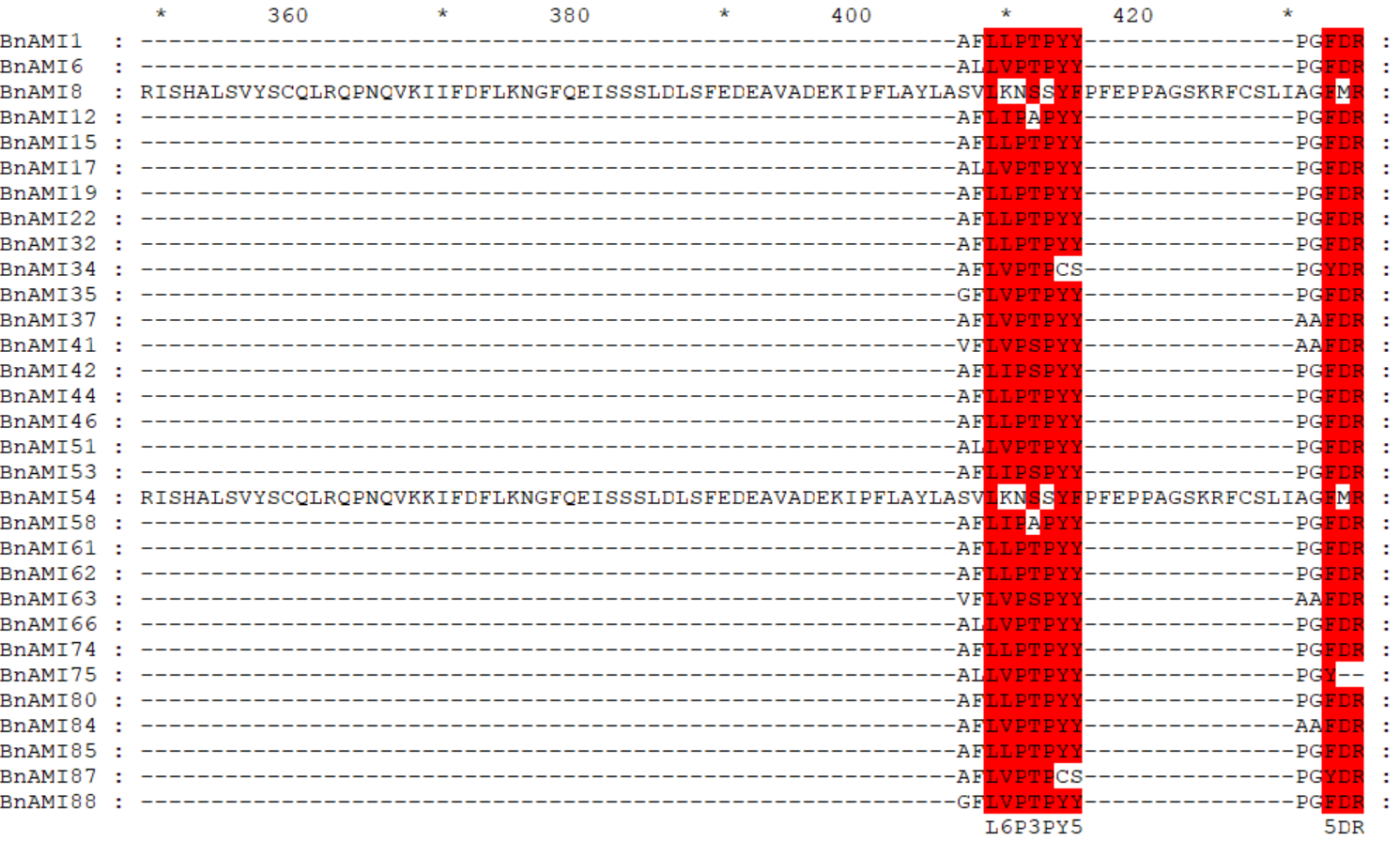


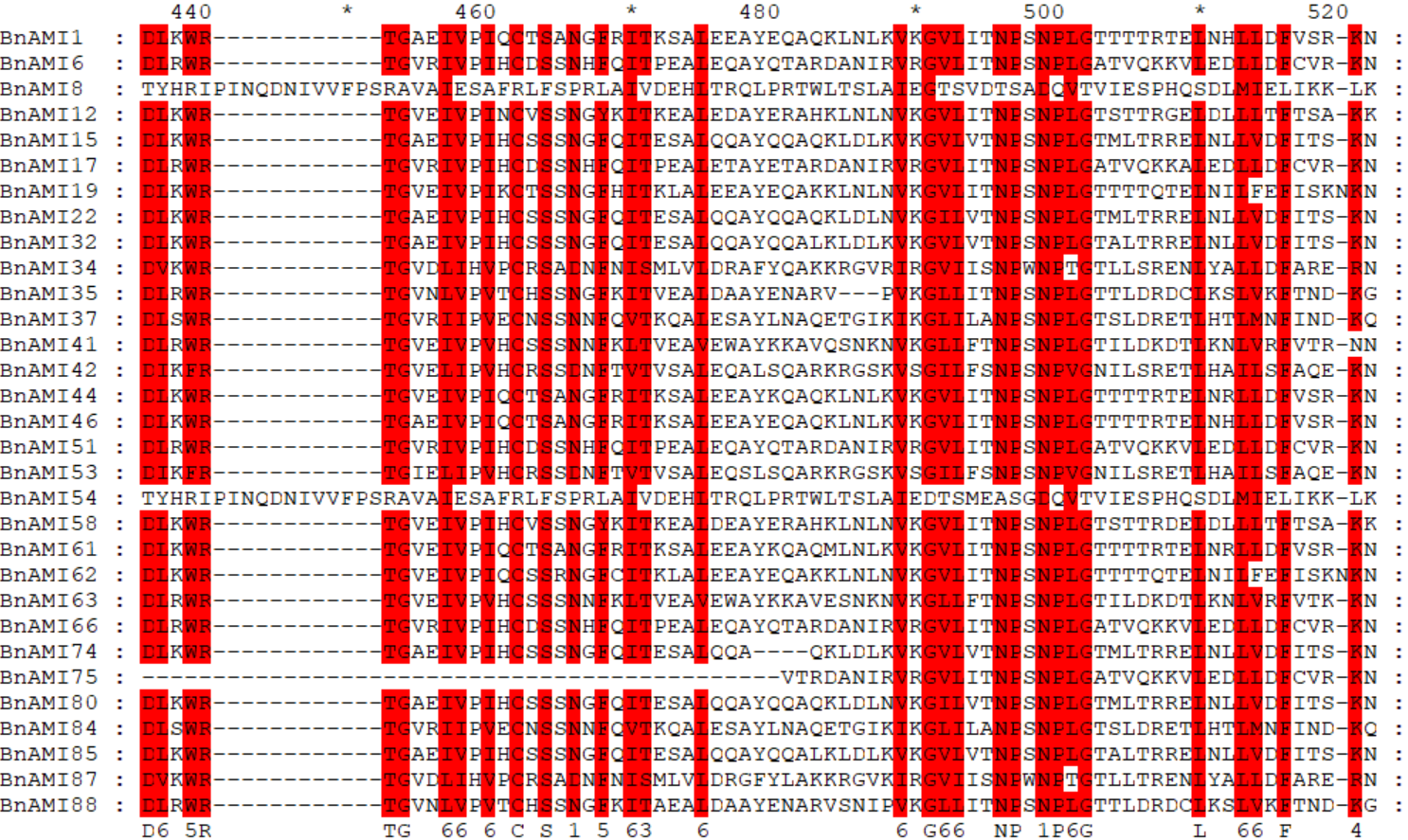


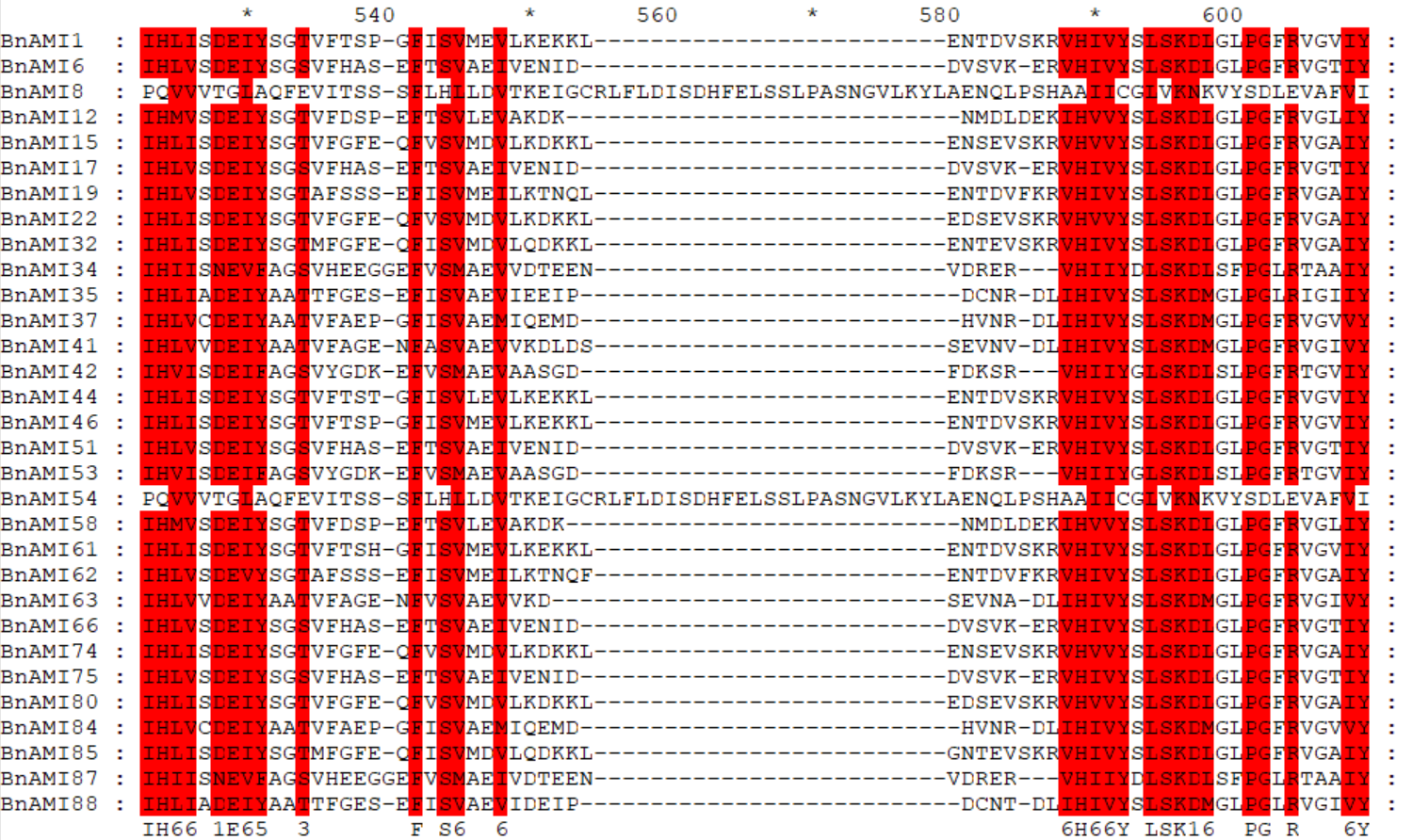


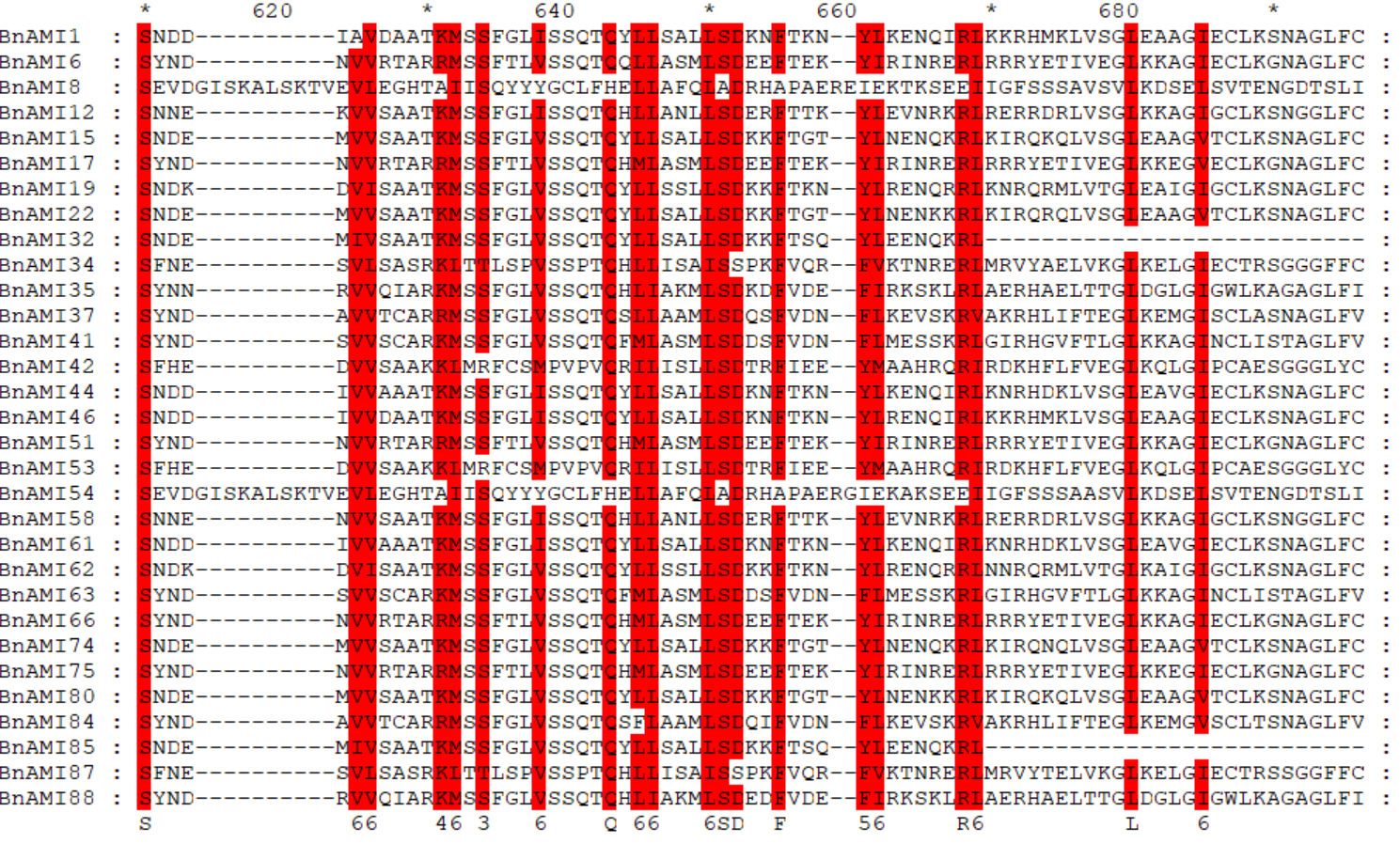


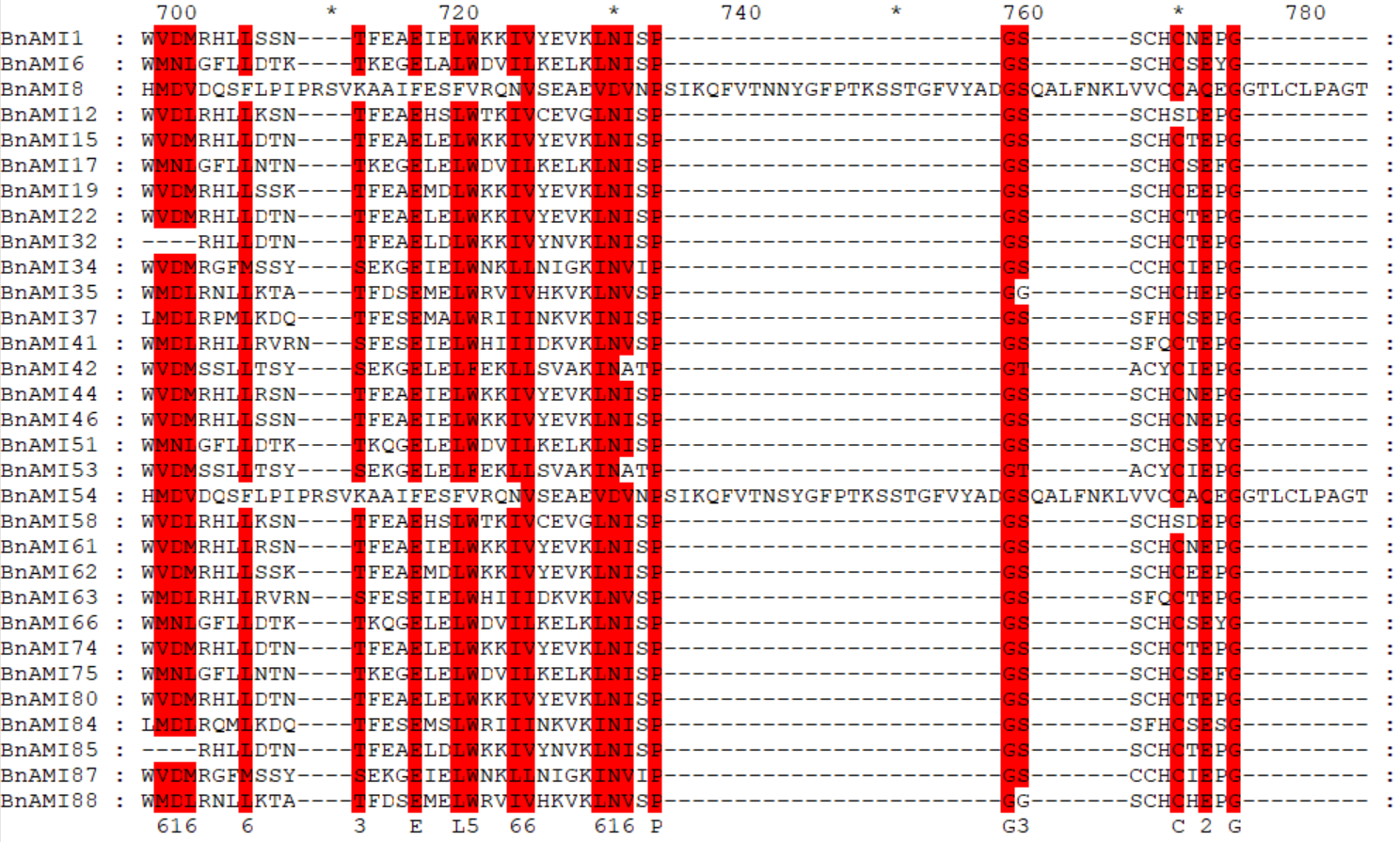


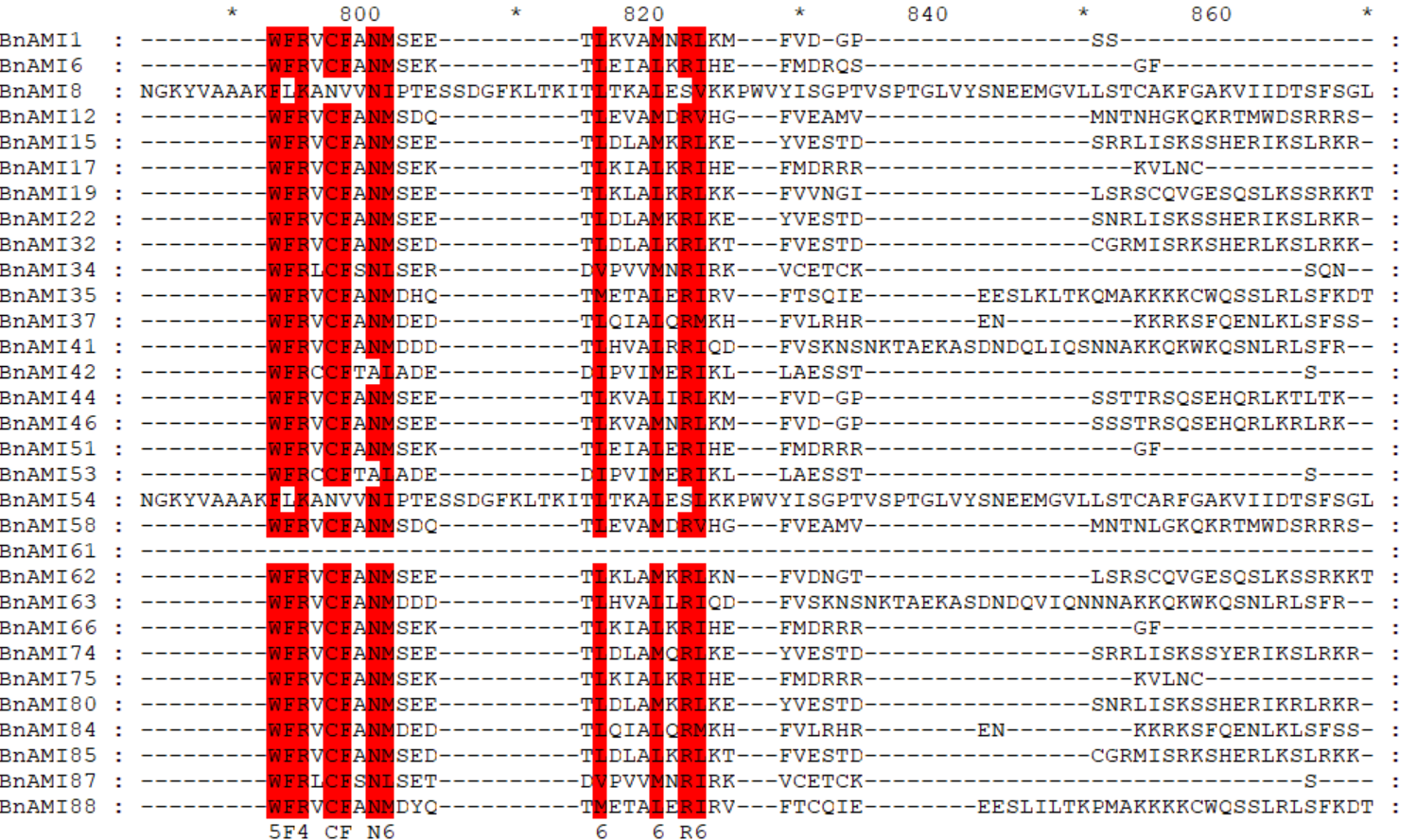


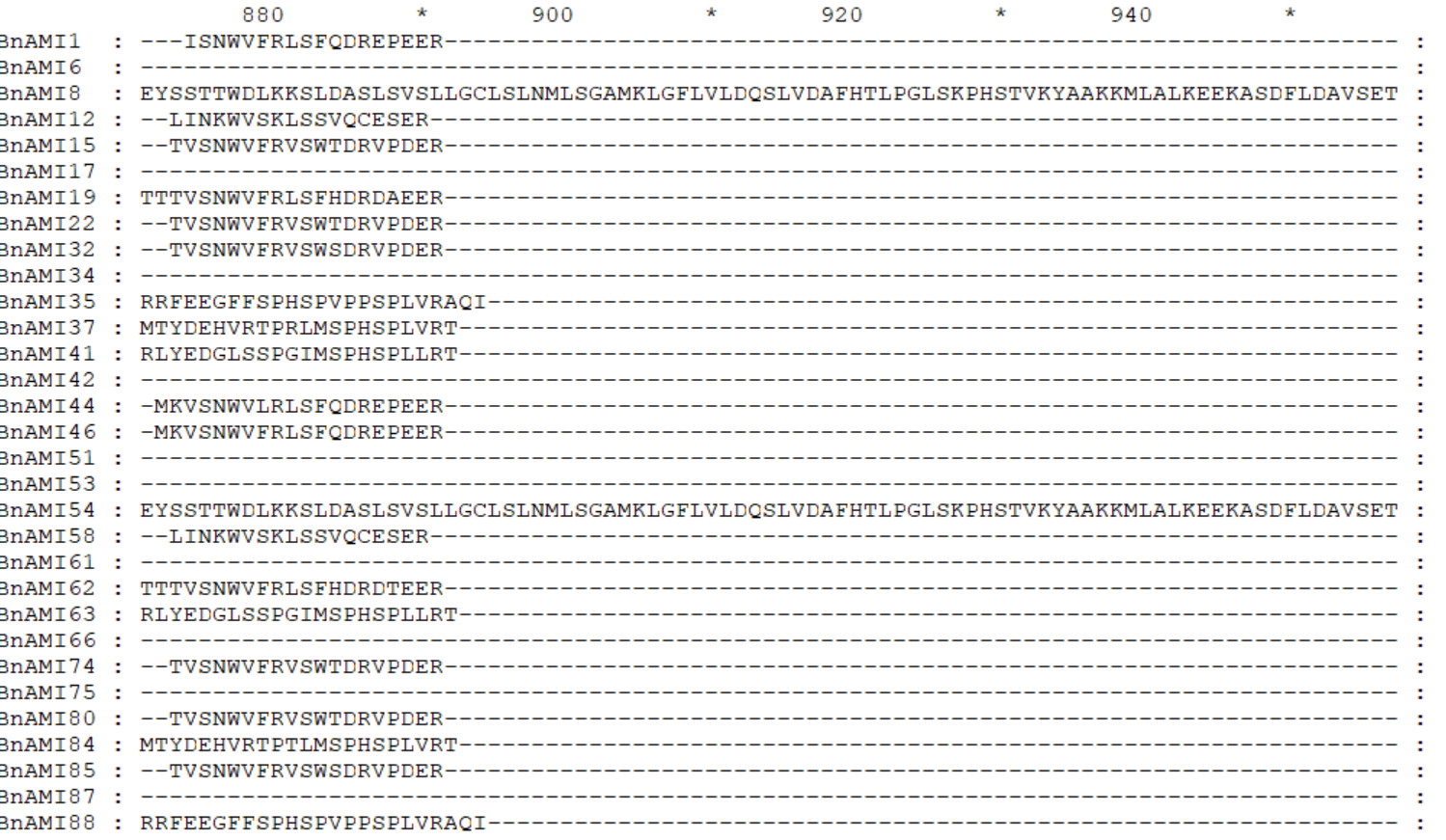


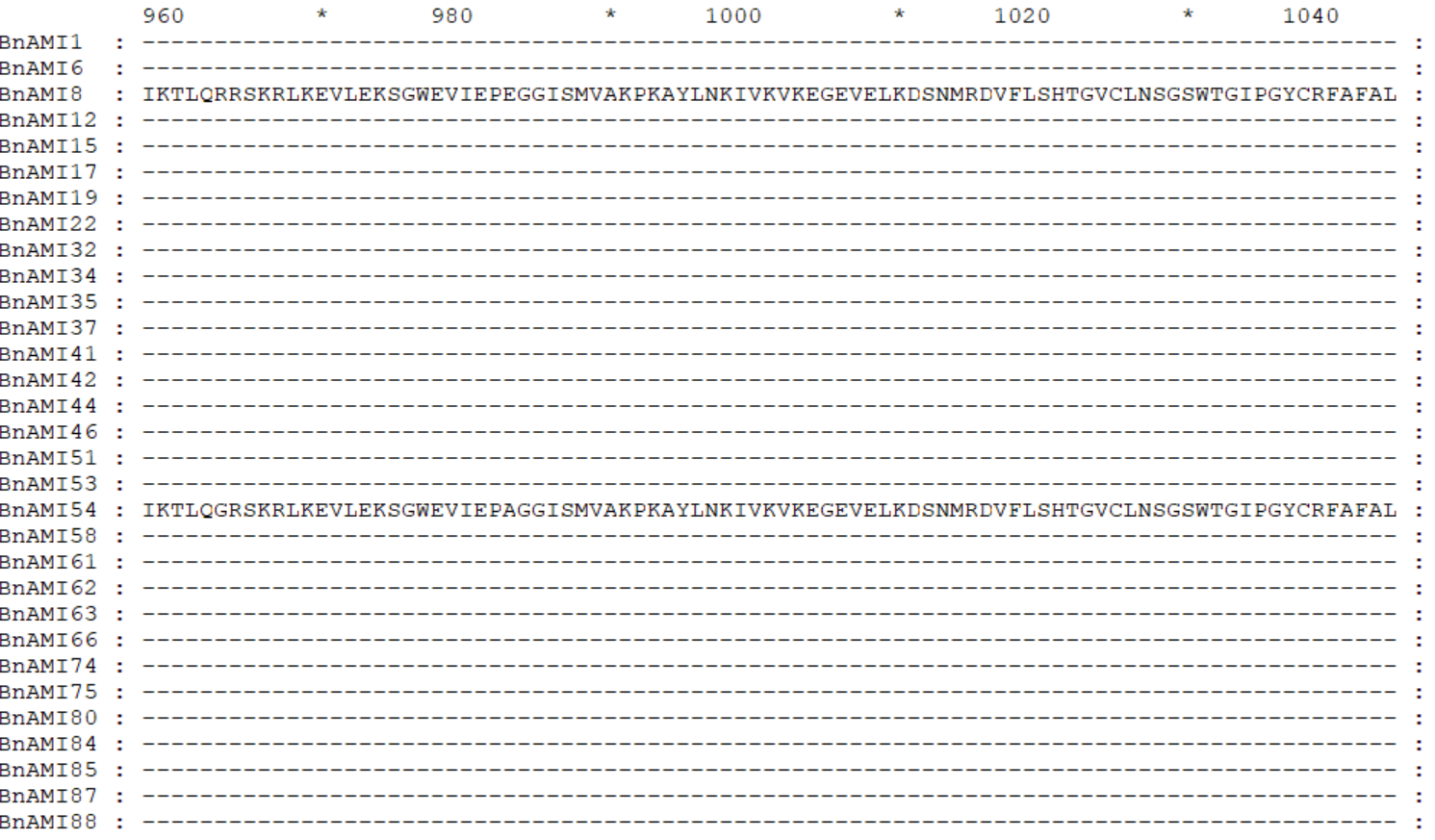


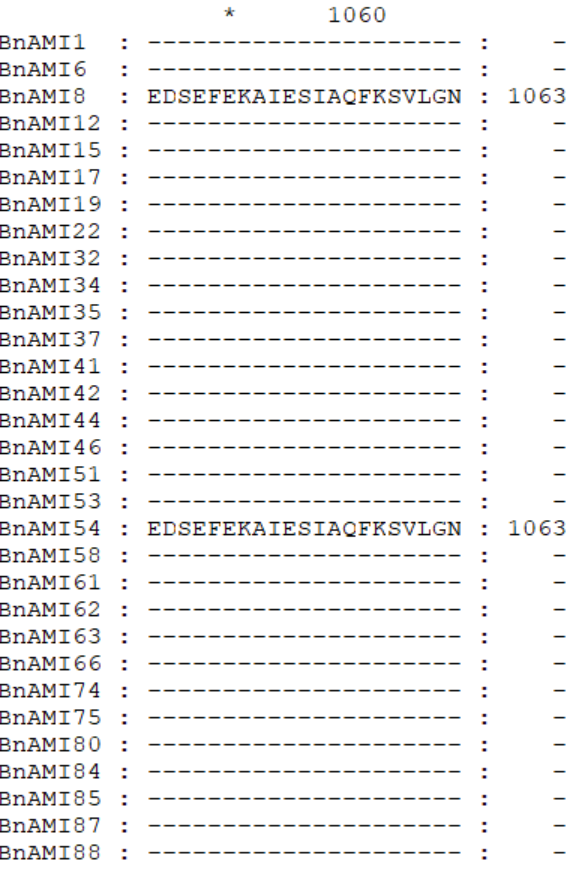


*BnTAR* proteins of the forth branch of *BnAMI* family containing *AMI8* and *AMI80*.（conservative property>90）

**Figure S2. Exon–intron organization of the *BnTAR* genes.** Round rectangles represent exons, and different colors indicate different branches of *BnTAR* gene. Black lines represent introns, and untranslated regions (UTRs) are marked by blue boxes. The exon and intron sizes can be estimated by the scale at the bottom.


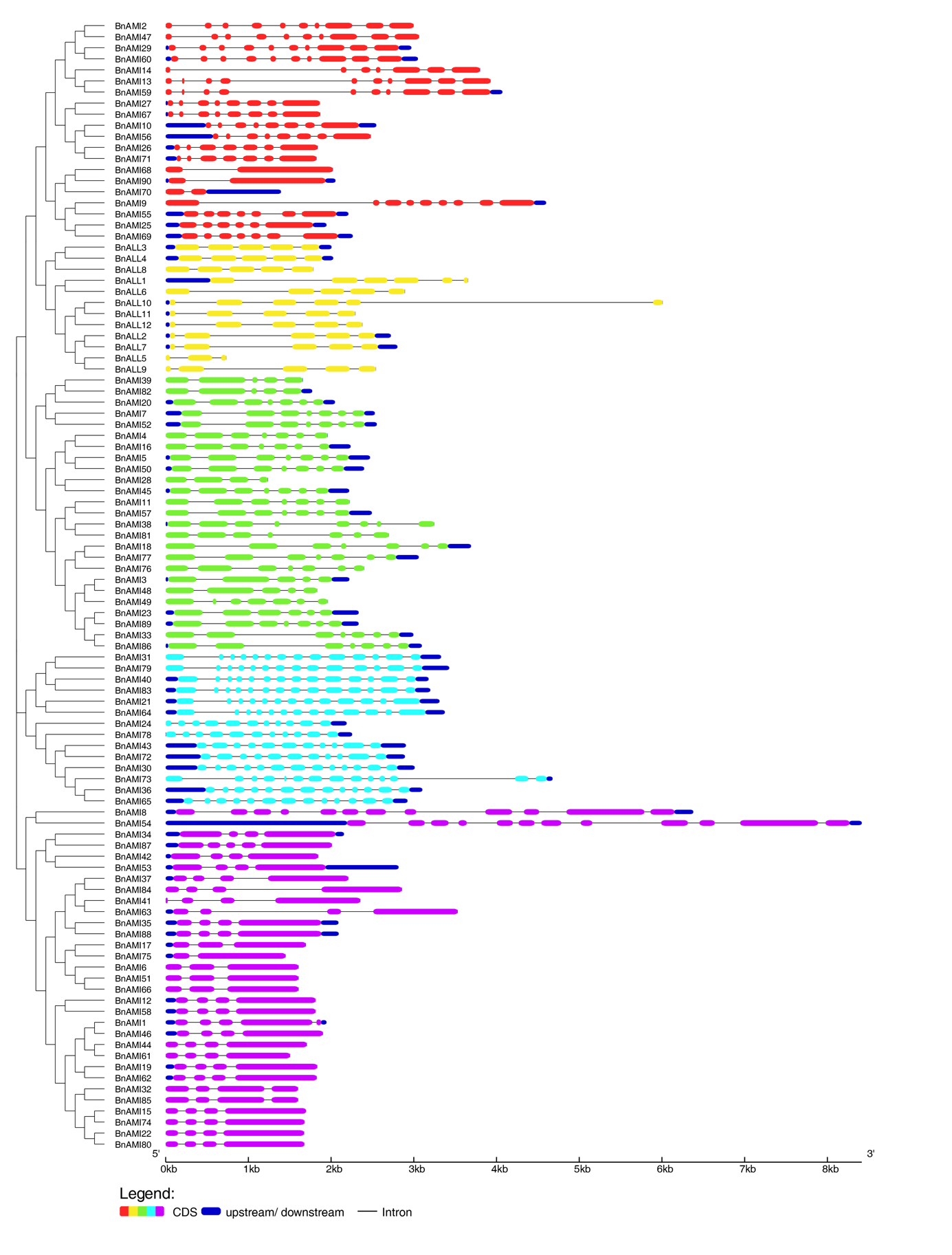


**Figure S3. Predicted secondary structure of *BnAMI1***. *BnAMI1* was an example to show that results from GOR4 prediction and PSIPRED is relatively the same. A: Results from GOR4 prediction analysis. B: Results from PSIPRED prediction analysis


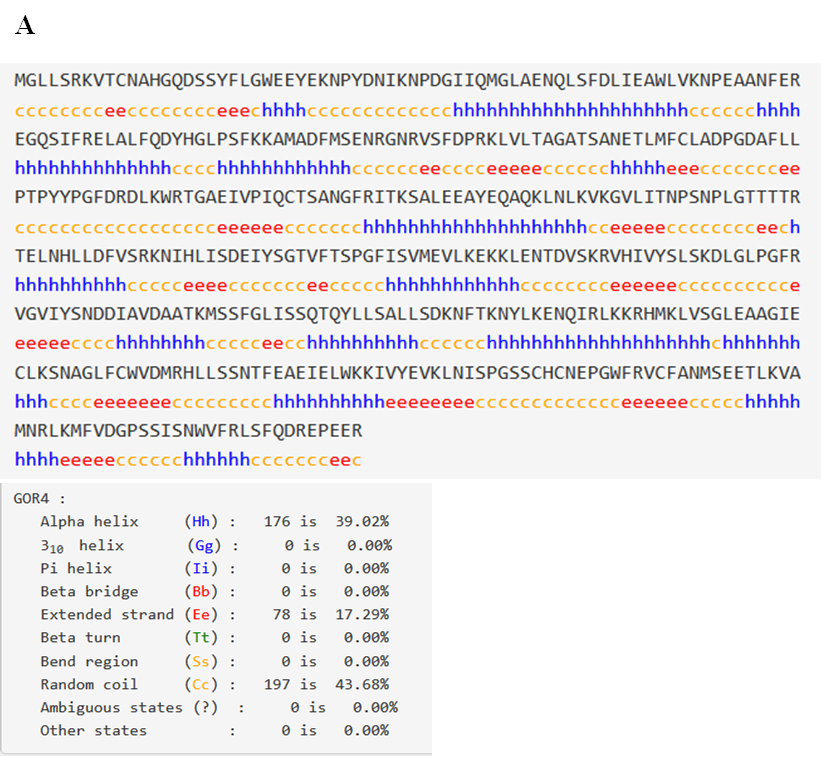


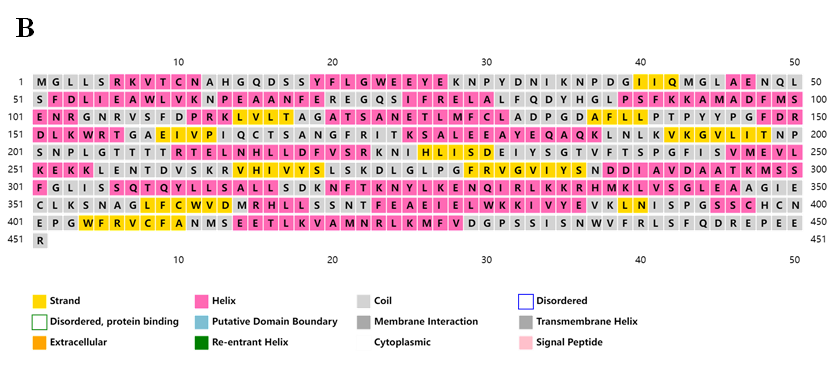


**Figure S4. Expression profiles of *BnTAR* genes in different tissues of rapeseed. The expression data of *BnTAR*s were collected from BnTIR.**


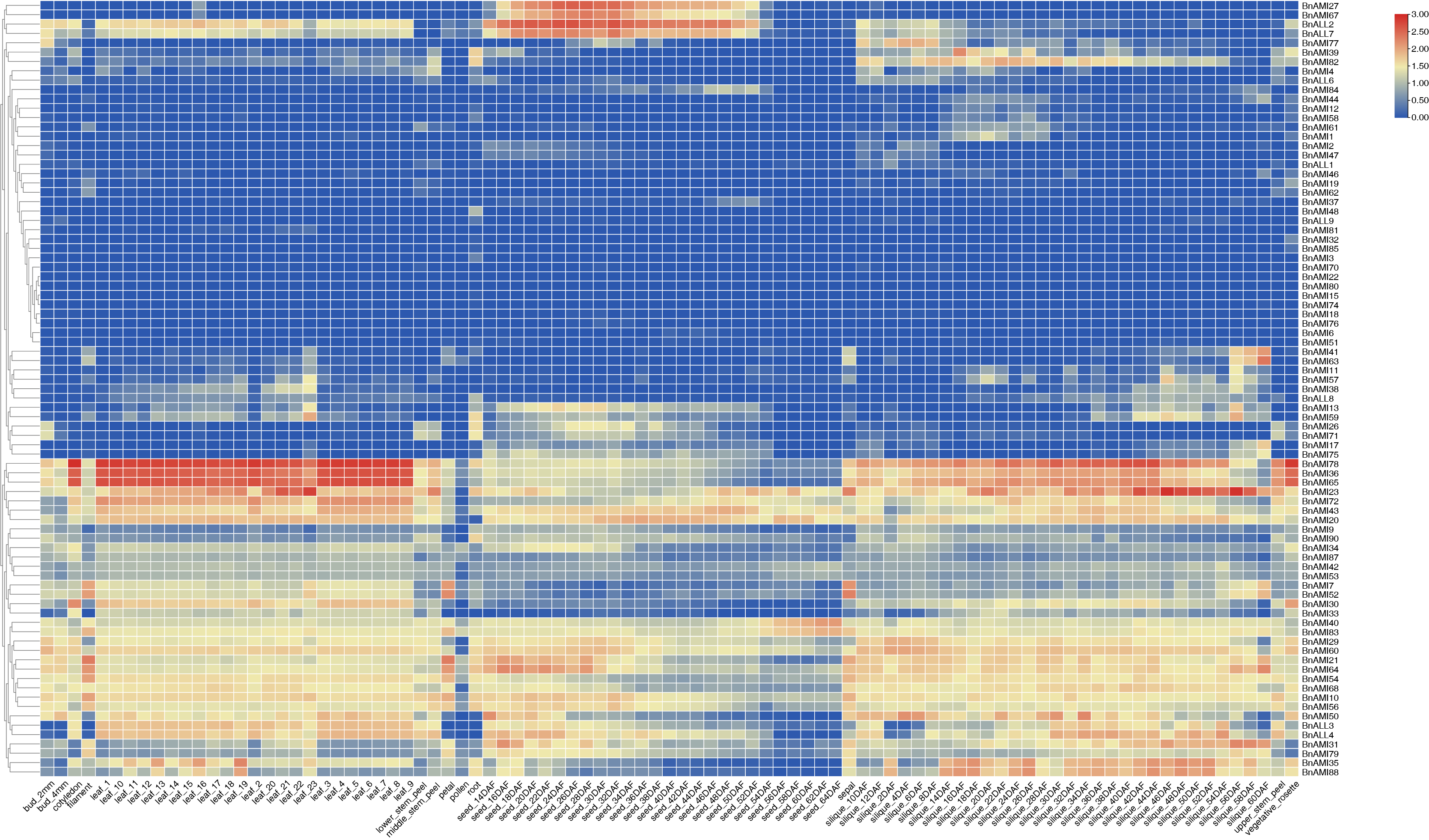


**Table S1. *TAR*s genes in *B. napus* genome with their sequence characteristics and subcellular location prediction**

| **Name** | **Gene ID** | **Family** | **Chr.** | **Gene position** | | **Gene Length(bp)** | **Protein Length(aa)** | **Mol. Wt.(Da)** | **pI** | **GRAVY** | **intro**  **number** | **Subcellular location** | |
| --- | --- | --- | --- | --- | --- | --- | --- | --- | --- | --- | --- | --- | --- |
|  |  |  |  | **Start** | **End** |  |  |  |  |  |  | **Plant-mPLoc** | **Multi Loc2** |
| *BnALL1* | BnaA01g14030D | Alliinase_C | A1 | 7126386 | 7130041 | 3656 | 445 | 53620.05 | 8.36 | -0.249 | 5 | Vacuole. | secretory pathway |
| *BnALL2* | BnaA02g14990D | Alliinase_C | A2 | 8623349 | 8626071 | 2723 | 381 | 62377.08 | 8.34 | -0.368 | 4 | Vacuole. | cytoplasmic |
| *BnALL3* | BnaA08g06520D | Alliinase_C | A8 | 6454243 | 6456248 | 2006 | 459 | 6687.58 | 8.26 | -0.231 | 4 | Chloroplast. Vacuole. | secretory pathway |
| *BnALL4* | BnaC08g07070D | Alliinase_C | C8 | 9779573 | 9781597 | 2025 | 458 | 53461.51 | 6.90 | -0.236 | 4 | Chloroplast. Vacuole. | secretory pathway |
| *BnALL5* | BnaAnng22040D | Alliinase_C | An-R | 24550738 | 24551470 | 733 | 132 | 44827.90 | 5.90 | -0.204 | 2 | Vacuole. | cytoplasmic |
| *BnALL6* | BnaC01g16530D | Alliinase_C | C1 | 11333347 | 11336238 | 2892 | 454 | 53615.25 | 8.52 | -0.218 | 4 | Chloroplast. Vacuole. | chloroplast |
| *BnALL7* | BnaC02g19980D | Alliinase_C | C2 | 16367662 | 16370463 | 2802 | 381 | 26640.92 | 5.28 | -0.354 | 4 | Vacuole. | cytoplasmic |
| *BnALL8* | BnaC06g07950D | Alliinase_C | C6 | 8625090 | 8626876 | 1787 | 475 | 26308.58 | 5.32 | -0.305 | 4 | Vacuole. | secretory pathway |
| *BnALL9* | BnaC06g43720D | Alliinase_C | C6-R | 3170690 | 3173231 | 2542 | 383 | 19410.51 | 9.15 | -0.412 | 4 | Vacuole. | cytoplasmic |
| *BnALL10* | BnaC05g18610D | Alliinase_C | C5 | 12306476 | 12312484 | 6009 | 412 | 43491.88 | 8.87 | -0.329 | 5 | Vacuole. | cytoplasmic |
| *BnALL11* | BnaA09g31200D | Alliinase_C | A9 | 23230993 | 23233285 | 2293 | 448 | 44186.19 | 5.59 | -0.34 | 4 | Vacuole. | cytoplasmic |
| *BnALL12* | BnaA09g31180D | Alliinase_C | A9 | 23196736 | 23199114 | 2379 | 381 | 47235.93 | 8.43 | -0.327 | 4 | Chloroplast. Vacuole. | cytoplasmic |
| *BnAMI1* | BnaA01g00270D | Aminotran_1_2 | A1 | 131736 | 133679 | 1944 | 451 | 52066.33 | 5.74 | -0.277 | 4 | Cytoplasm. | chloroplast |
| *BnAMI2* | BnaA01g03290D | Aminotran_1_2 | A1 | 1573590 | 1576588 | 2999 | 458 | 59293.19 | 6.20 | -0.176 | 9 | Chloroplast. | chloroplast |
| *BnAMI3* | BnaA01g08470D | Aminotran_1_2 | A1 | 4040670 | 4042890 | 2221 | 456 | 19318.55 | 4.78 | -0.121 | 4 | Chloroplast. | cytoplasmic |
| *BnAMI4* | BnaA01g13270D | Aminotran_1_2 | A1 | 6707424 | 6709383 | 1960 | 424 | 27183.38 | 6.11 | -0.052 | 6 | Chloroplast. | cytoplasmic |
| *BnAMI5* | BnaA01g13280D | Aminotran_1_2 | A1 | 6719596 | 6722066 | 2471 | 424 | 49934.66 | 6.04 | -0.132 | 6 | Chloroplast. | cytoplasmic |
| *BnAMI6* | BnaA01g15380D | Aminotran_1_2 | A1 | 7874005 | 7875611 | 1607 | 448 | 44636.45 | 5.28 | -0.306 | 2 | Chloroplast. | secretory pathway |
| *BnAMI7* | BnaA02g09960D | Aminotran_1_2 | A2 | 4984418 | 4986946 | 2529 | 418 | 41204.51 | 8.23 | 0.094 | 6 | Chloroplast. | chloroplast |
| *BnAMI8* | BnaA02g11830D | Aminotran_1_2 | A2 | 6203381 | 6209759 | 6379 | 1063 | 49235.87 | 6.24 | -0.032 | 11 | Chloroplast. Cytoplasm. | nuclear |
| *BnAMI9* | BnaA02g15650D | Aminotran_1_2 | A2 | 9110227 | 9114826 | 4600 | 556 | 46515.60 | 5.19 | -0.285 | 8 | Chloroplast. | chloroplast |
| *BnAMI10* | BnaA02g19790D | Aminotran_1_2 | A2 | 12262461 | 12265007 | 2547 | 394 | 50781.82 | 6.44 | -0.093 | 7 | Chloroplast. | secretory pathway |
| *BnAMI11* | BnaA03g22790D | Aminotran_1_2 | A3 | 10833279 | 10835504 | 2226 | 439 | 12870.63 | 6.33 | -0.114 | 6 | Chloroplast. | chloroplast |
| *BnAMI12* | BnaA03g23600D | Aminotran_1_2 | A3 | 11297894 | 11299707 | 1814 | 464 | 59141.55 | 6.12 | -0.329 | 3 | Cytoplasm. | nuclear |
| *BnAMI13* | BnaA03g38440D | Aminotran_1_2 | A3 | 19109547 | 19113476 | 3930 | 456 | 50050.45 | 5.93 | -0.142 | 9 | Chloroplast. | chloroplast |
| *BnAMI14* | BnaA03g38470D | Aminotran_1_2 | A3 | 19121255 | 19125056 | 3802 | 380 | 50322.75 | 5.81 | -0.102 | 6 | Chloroplast. | cytoplasmic |
| *BnAMI15* | BnaA03g40560D | Aminotran_1_2 | A3 | 20276244 | 20277938 | 1695 | 470 | 28748.13 | 6.11 | -0.303 | 3 | Cytoplasm. | cytoplasmic |
| *BnAMI16* | BnaA03g46060D | Aminotran_1_2 | A3 | 23544351 | 23546586 | 2236 | 412 | 19396.69 | 5.02 | -0.121 | 6 | Chloroplast. | nuclear |
| *BnAMI17* | BnaA03g47880D | Aminotran_1_2 | A3 | 24633222 | 24634916 | 1695 | 451 | 51426.35 | 6.89 | -0.353 | 2 | Chloroplast. | cytoplasmic |
| *BnAMI18* | BnaA03g49250D | Aminotran_1_2 | A3 | 25378962 | 25382653 | 3692 | 448 | 50215.59 | 8.49 | -0.088 | 6 | Chloroplast. | secretory pathway |
| *BnAMI19* | BnaA04g13310D | Aminotran_1_2 | A4 | 11248000 | 11249833 | 1834 | 474 | 7263.62 | 9.57 | -0.285 | 3 | Cytoplasm. | nuclear |
| *BnAMI20* | BnaA05g12790D | Aminotran_1_2 | A5 | 7437244 | 7439290 | 2047 | 420 | 19838.24 | 4.99 | 0.054 | 6 | Chloroplast. | secretory pathway |
| *BnAMI21* | BnaA06g11610D | Aminotran_1_2 | A6 | 6030589 | 6033899 | 3311 | 537 | 50504.21 | 5.28 | -0.223 | 12 | Chloroplast. | chloroplast |
| *BnAMI22* | BnaA06g15630D | Aminotran_1_2 | A6 | 8566879 | 8568552 | 1674 | 470 | 26574.54 | 6.13 | -0.309 | 3 | Cytoplasm. | cytoplasmic |
| *BnAMI23* | BnaA07g00460D | Aminotran_1_2 | A7 | 342702 | 345035 | 2334 | 453 | 13520.62 | 5.34 | -0.108 | 5 | Chloroplast. | cytoplasmic |
| *BnAMI24* | BnaA07g10020D | Aminotran_1_2 | A7 | 9589053 | 9591240 | 2188 | 403 | 44667.02 | 5.37 | -0.179 | 10 | Chloroplast. | cytoplasmic |
| *BnAMI25* | BnaA07g29720D | Aminotran_1_2 | A7 | 21213276 | 21215219 | 1944 | 412 | 20095.45 | 5.00 | -0.17 | 5 | Chloroplast. | chloroplast |
| *BnAMI26* | BnaA07g35690D | Aminotran_1_2 | A7 | 23916833 | 23918671 | 1839 | 394 | 51197.14 | 5.95 | -0.17 | 6 | Chloroplast. | nuclear |
| *BnAMI27* | BnaA07g38380D | Aminotran_1_2 | A7-R | 1602028 | 1603892 | 1865 | 394 | 40673.90 | 5.34 | -0.196 | 7 | Chloroplast. | chloroplast |
| *BnAMI28* | BnaA08g10670D | Aminotran_1_2 | A8 | 9902893 | 9904129 | 1237 | 303 | 8809.00 | 5.00 | -0.05 | 3 | Chloroplast. | cytoplasmic |
| *BnAMI29* | BnaA08g11470D | Aminotran_1_2 | A8 | 10435175 | 10438144 | 2970 | 461 | 50617.46 | 5.78 | -0.171 | 9 | Chloroplast. | chloroplast |
| *BnAMI30* | BnaA08g20540D | Aminotran_1_2 | A8 | 15470520 | 15473530 | 3011 | 478 | 52826.71 | 6.20 | -0.165 | 12 | Chloroplast. | cytoplasmic |
| *BnAMI31* | BnaA08g23190D | Aminotran_1_2 | A8 | 16607739 | 16611068 | 3330 | 542 | 49289.87 | 6.19 | -0.159 | 13 | Chloroplast. | chloroplast |
| *BnAMI32* | BnaA09g07210D | Aminotran_1_2 | A9 | 3580147 | 3581748 | 1602 | 439 | 14068.20 | 7.63 | -0.351 | 3 | Chloroplast. | cytoplasmic |
| *BnAMI33* | BnaA09g10030D | Aminotran_1_2 | A9 | 5083201 | 5086195 | 2995 | 446 | 32998.19 | 5.35 | -0.08 | 6 | Chloroplast. | cytoplasmic |
| *BnAMI34* | BnaA09g13010D | Aminotran_1_2 | A9 | 7069175 | 7071330 | 2156 | 543 | 33205.05 | 7.75 | -0.22 | 3 | Chloroplast. | cytoplasmic |
| *BnAMI35* | BnaA09g21860D | Aminotran_1_2 | A9 | 14487657 | 14489745 | 2089 | 488 | 53262.03 | 6.41 | -0.24 | 3 | Chloroplast. | cytoplasmic |
| *BnAMI36* | BnaA09g31220D | Aminotran_1_2 | A9 | 23246440 | 23249542 | 3103 | 478 | 53068.91 | 6.30 | -0.198 | 12 | Chloroplast. | cytoplasmic |
| *BnAMI37* | BnaA09g39260D | Aminotran_1_2 | A9 | 27849701 | 27851910 | 2210 | 473 | 43552.37 | 6.12 | -0.202 | 3 | Chloroplast. | nuclear |
| *BnAMI38* | BnaA09g41170D | Aminotran_1_2 | A9 | 28809464 | 28812712 | 3249 | 464 | 13605.32 | 5.12 | -0.064 | 7 | Chloroplast. | chloroplast |
| *BnAMI39* | BnaA09g43670D | Aminotran_1_2 | A9 | 30171222 | 30172880 | 1659 | 423 | 49801.64 | 5.98 | -0.122 | 4 | Chloroplast. | cytoplasmic |
| *BnAMI40* | BnaA09g45030D | Aminotran_1_2 | A9 | 30878916 | 30882094 | 3179 | 547 | 42652.30 | 5.80 | -0.248 | 13 | Chloroplast. | chloroplast |
| *BnAMI41* | BnaA10g00470D | Aminotran_1_2 | A10 | 244908 | 247262 | 2355 | 447 | 43683.20 | 6.04 | -0.132 | 3 | Chloroplast. | cytoplasmic |
| *BnAMI42* | BnaAnng08090D | Aminotran_1_2 | An-R | 8664247 | 8666092 | 1846 | 500 | 12980.97 | 6.75 | 0.035 | 3 | Chloroplast. | chloroplast |
| *BnAMI43* | BnaAnng22050D | Aminotran_1_2 | An-R | 24554712 | 24557615 | 2904 | 481 | 53505.47 | 6.30 | -0.197 | 10 | Chloroplast. | cytoplasmic |
| *BnAMI44* | BnaAnng30490D | Aminotran_1_2 | An-R | 34836790 | 34838496 | 1707 | 469 | 59573.04 | 6.25 | -0.32 | 3 | Cytoplasm. | cytoplasmic |
| *BnAMI45* | BnaAnng31450D | Aminotran_1_2 | An-R | 35945461 | 35947679 | 2219 | 423 | 22884.85 | 7.00 | -0.169 | 6 | Chloroplast. | cytoplasmic |
| *BnAMI46* | BnaC01g01270D | Aminotran_1_2 | C1 | 583555 | 585457 | 1903 | 469 | 7094.25 | 9.31 | -0.346 | 3 | Cytoplasm. | cytoplasmic |
| *BnAMI47* | BnaC01g04560D | Aminotran_1_2 | C1 | 2410054 | 2413118 | 3065 | 457 | 57179.88 | 5.74 | 0.161 | 9 | Chloroplast. | chloroplast |
| *BnAMI48* | BnaC01g10020D | Aminotran_1_2 | C1 | 5905188 | 5907019 | 1832 | 456 | 50641.27 | 5.29 | -0.158 | 4 | Chloroplast. | cytoplasmic |
| *BnAMI49* | BnaC01g10040D | Aminotran_1_2 | C1 | 5908920 | 5910880 | 1961 | 382 | 28992.69 | 6.04 | -0.113 | 6 | Chloroplast. | cytoplasmic |
| *BnAMI50* | BnaC01g15060D | Aminotran_1_2 | C1 | 10218671 | 10221070 | 2400 | 424 | 20376.85 | 8.26 | -0.133 | 6 | Chloroplast. | cytoplasmic |
| *BnAMI51* | BnaC01g18250D | Aminotran_1_2 | C1 | 12691453 | 12693059 | 1607 | 448 | 18411.17 | 9.12 | -0.331 | 2 | Chloroplast. | secretory pathway |
| *BnAMI52* | BnaC02g13860D | Aminotran_1_2 | C2 | 9176217 | 9178769 | 2553 | 418 | 13600.26 | 5.10 | 0.078 | 6 | Chloroplast. | chloroplast |
| *BnAMI53* | BnaC02g15560D | Aminotran_1_2 | C2 | 11176515 | 11179330 | 2816 | 500 | 32789.97 | 8.04 | 0.041 | 3 | Chloroplast. | chloroplast |
| *BnAMI54* | BnaC02g16260D | Aminotran_1_2 | C2 | 11883493 | 11891907 | 8415 | 1063 | 49991.53 | 5.59 | -0.027 | 11 | Chloroplast. Cytoplasm. | nuclear |
| *BnAMI55* | BnaC02g20870D | Aminotran_1_2 | C2 | 17498349 | 17500557 | 2209 | 410 | 46455.52 | 5.12 | -0.144 | 6 | Chloroplast. | chloroplast |
| *BnAMI56* | BnaC02g23160D | Aminotran_1_2 | C2 | 20182982 | 20185461 | 2480 | 394 | 43885.93 | 7.61 | -0.112 | 7 | Chloroplast. | secretory pathway |
| *BnAMI57* | BnaC03g26850D | Aminotran_1_2 | C3 | 15376695 | 15379187 | 2493 | 439 | 20213.29 | 5.48 | -0.149 | 6 | Chloroplast. | chloroplast |
| *BnAMI58* | BnaC03g28000D | Aminotran_1_2 | C3 | 16406448 | 16408261 | 1814 | 464 | 59185.63 | 6.25 | -0.32 | 3 | Cytoplasm. | nuclear |
| *BnAMI59* | BnaC03g45280D | Aminotran_1_2 | C3 | 30294324 | 30298394 | 4071 | 456 | 67439.19 | 5.53 | -0.136 | 9 | Chloroplast. | chloroplast |
| *BnAMI60* | BnaC03g66430D | Aminotran_1_2 | C3 | 56246285 | 56249334 | 3050 | 461 | 67405.24 | 5.67 | -0.171 | 9 | Chloroplast. | chloroplast |
| *BnAMI61* | BnaC03g76570D | Aminotran_1_2 | C3-R | 5174376 | 5175881 | 1506 | 403 | 60195.51 | 6.05 | -0.264 | 3 | Cytoplasm. | cytoplasmic |
| *BnAMI62* | BnaC04g35390D | Aminotran_1_2 | C4 | 36928648 | 36930475 | 1828 | 474 | 14276.41 | 8.34 | -0.239 | 3 | Cytoplasm. | nuclear |
| *BnAMI63* | BnaC05g00530D | Aminotran_1_2 | C5 | 303635 | 307165 | 3531 | 496 | 43910.90 | 6.77 | -0.205 | 3 | Chloroplast. | cytoplasmic |
| *BnAMI64* | BnaC05g13450D | Aminotran_1_2 | C5 | 7756022 | 7759396 | 3375 | 540 | 46879.75 | 5.43 | -0.241 | 12 | Chloroplast. | chloroplast |
| *BnAMI65* | BnaC05g18600D | Aminotran_1_2 | C5 | 12303489 | 12306412 | 2924 | 478 | 53068.91 | 6.30 | -0.198 | 12 | Chloroplast. | cytoplasmic |
| *BnAMI66* | BnaCnng71530D | Aminotran_1_2 | Cn-R | 71787537 | 71789143 | 1607 | 448 | 6655.73 | 7.96 | -0.311 | 2 | Chloroplast. | secretory pathway |
| *BnAMI67* | BnaC06g19110D | Aminotran_1_2 | C6 | 21473977 | 21475845 | 1869 | 394 | 6584.87 | 9.62 | -0.156 | 7 | Chloroplast. | nuclear |
| *BnAMI68* | BnaC06g20760D | Aminotran_1_2 | C6 | 22828400 | 22830423 | 2024 | 452 | 50117.41 | 5.63 | -0.111 | 1 | Chloroplast. | chloroplast |
| *BnAMI69* | BnaC06g32920D | Aminotran_1_2 | C6 | 32965299 | 32967560 | 2262 | 400 | 19718.07 | 4.87 | -0.134 | 6 | Chloroplast. | cytoplasmic |
| *BnAMI70* | BnaC06g38270D | Aminotran_1_2 | C6 | 35901092 | 35902485 | 1394 | 134 | 15142.61 | 9.30 | -0.144 | 1 | Chloroplast. | secretory pathway |
| *BnAMI71* | BnaC06g40630D | Aminotran_1_2 | C6 | 37178529 | 37180354 | 1826 | 393 | 50799.66 | 5.85 | -0.162 | 6 | Chloroplast. | nuclear |
| *BnAMI72* | BnaC06g43710D | Aminotran_1_2 | C6-R | 3159376 | 3162268 | 2893 | 481 | 44476.99 | 5.83 | -0.195 | 10 | Chloroplast. | cytoplasmic |
| *BnAMI73* | BnaC07g13140D | Aminotran_1_2 | C7 | 18633661 | 18638338 | 4678 | 537 | 6661.47 | 9.68 | -0.247 | 14 | Chloroplast. | cytoplasmic |
| *BnAMI74* | BnaC07g31520D | Aminotran_1_2 | C7 | 35578972 | 35580652 | 1681 | 466 | 26554.53 | 6.13 | -0.286 | 3 | Cytoplasm. | cytoplasmic |
| *BnAMI75* | BnaC07g40130D | Aminotran_1_2 | C7 | 40677281 | 40678730 | 1450 | 416 | 47217.52 | 6.50 | -0.347 | 1 | Chloroplast. | cytoplasmic |
| *BnAMI76* | BnaC07g41260D | Aminotran_1_2 | C7 | 41229103 | 41231503 | 2401 | 442 | 55464.33 | 8.03 | -0.052 | 6 | Chloroplast. | cytoplasmic |
| *BnAMI77* | BnaC07g41280D | Aminotran_1_2 | C7 | 41247649 | 41250708 | 3060 | 448 | 53409.14 | 6.29 | -0.113 | 6 | Chloroplast. | cytoplasmic |
| *BnAMI78* | BnaC08g06270D | Aminotran_1_2 | C8 | 8723809 | 8726063 | 2255 | 403 | 52987.64 | 6.31 | -0.158 | 10 | Chloroplast. | cytoplasmic |
| *BnAMI79* | BnaC08g17310D | Aminotran_1_2 | C8 | 20899144 | 20902571 | 3428 | 541 | 46933.64 | 5.72 | -0.175 | 13 | Chloroplast. | chloroplast |
| *BnAMI80* | BnaC08g21300D | Aminotran_1_2 | C8 | 23922853 | 23924530 | 1678 | 470 | 28764.13 | 6.11 | -0.317 | 3 | Cytoplasm. | cytoplasmic |
| *BnAMI81* | BnaC08g33730D | Aminotran_1_2 | C8 | 32101931 | 32104628 | 2698 | 444 | 8343.73 | 9.44 | -0.05 | 6 | Chloroplast. | chloroplast |
| *BnAMI82* | BnaC08g36300D | Aminotran_1_2 | C8 | 33631049 | 33632820 | 1772 | 423 | 20067.40 | 5.00 | -0.083 | 4 | Chloroplast. | cytoplasmic |
| *BnAMI83* | BnaC08g37860D | Aminotran_1_2 | C8 | 34436600 | 34439798 | 3199 | 549 | 47193.48 | 7.59 | -0.224 | 13 | Chloroplast. | chloroplast |
| *BnAMI84* | BnaC08g49010D | Aminotran_1_2 | C8-R | 3914886 | 3917742 | 2857 | 473 | 43602.15 | 5.80 | -0.198 | 3 | Chloroplast. | nuclear |
| *BnAMI85* | BnaC09g07070D | Aminotran_1_2 | C9 | 4435700 | 4437300 | 1601 | 439 | 45904.10 | 5.89 | -0.349 | 3 | Chloroplast. | cytoplasmic |
| *BnAMI86* | BnaC09g10110D | Aminotran_1_2 | C9 | 6713194 | 6716290 | 3097 | 446 | 51084.62 | 4.94 | -0.099 | 6 | Chloroplast. | cytoplasmic |
| *BnAMI87* | BnaC09g12920D | Aminotran_1_2 | C9 | 9496268 | 9498279 | 2012 | 523 | 47210.57 | 8.25 | -0.187 | 4 | Chloroplast. | cytoplasmic |
| *BnAMI88* | BnaC09g24050D | Aminotran_1_2 | C9 | 21919282 | 21921373 | 2092 | 491 | 43681.39 | 5.72 | -0.205 | 3 | Chloroplast. | cytoplasmic |
| *BnAMI89* | BnaCnng43090D | Aminotran_1_2 | Cn-R | 42225315 | 42227647 | 2333 | 402 | 49015.51 | 5.37 | -0.12 | 6 | Chloroplast. | nuclear |
| *BnAMI90* | BnaCnng64190D | Aminotran_1_2 | Cn-R | 63927318 | 63929370 | 2053 | 452 | 49987.25 | 5.63 | -0.079 | 1 | Chloroplast. | chloroplast |

**Table S2. Data of subcellular location prediction of** **Multi Loc2.** The predicted result of Plant-mPLoc was given directly without the detail. So only the data of Multi Loc2 were shown.

| Gene name | cytoplasmic | nuclear | chloroplast | mitochondrial | secretory pathway |
| --- | --- | --- | --- | --- | --- |
| *BnALL1* | 0.17 | 0.05 | 0.3 | 0.17 | 0.31 |
| *BnALL2* | 0.56 | 0.06 | 0.08 | 0.03 | 0.27 |
| *BnALL3* | 0.19 | 0.1 | 0.21 | 0.12 | 0.38 |
| *BnALL4* | 0.19 | 0.1 | 0.21 | 0.12 | 0.38 |
| *BnALL5* | 0.41 | 0.07 | 0.1 | 0.05 | 0.37 |
| *BnALL6* | 0.18 | 0.06 | 0.31 | 0.19 | 0.27 |
| *BnALL7* | 0.54 | 0.06 | 0.07 | 0.03 | 0.3 |
| *BnALL8* | 0.2 | 0.11 | 0.23 | 0.12 | 0.34 |
| *BnALL9* | 0.51 | 0.06 | 0.06 | 0.03 | 0.34 |
| *BnALL10* | 0.61 | 0.06 | 0.06 | 0.02 | 0.25 |
| *BnALL11* | 0.58 | 0.07 | 0.05 | 0.02 | 0.28 |
| *BnALL12* | 0.48 | 0.06 | 0.05 | 0.02 | 0.39 |
| *BnAMI1* | 0.73 | 0.05 | 0.09 | 0.02 | 0.11 |
| *BnAMI2* | 0.05 | 0.01 | 0.67 | 0.26 | 0.01 |
| *BnAMI3* | 0.65 | 0.06 | 0.07 | 0.02 | 0.21 |
| *BnAMI4* | 0.59 | 0.05 | 0.11 | 0.02 | 0.23 |
| *BnAMI5* | 0.56 | 0.06 | 0.11 | 0.02 | 0.26 |
| *BnAMI6* | 0.44 | 0.07 | 0.04 | 0.01 | 0.44 |
| *BnAMI7* | 0.24 | 0.14 | 0.26 | 0.1 | 0.25 |
| *BnAMI8* | 0.08 | 0.77 | 0.12 | 0.03 | 0.0 |
| *BnAMI9* | 0.03 | 0.45 | 0.51 | 0.01 | 0.01 |
| *BnAMI10* | 0.27 | 0.06 | 0.08 | 0.03 | 0.56 |
| *BnAMI11* | 0.13 | 0.08 | 0.71 | 0.07 | 0.01 |
| *BnAMI12* | 0.07 | 0.84 | 0.07 | 0.02 | 0.0 |
| *BnAMI13* | 0.06 | 0.01 | 0.63 | 0.29 | 0.01 |
| *BnAMI14* | 0.73 | 0.04 | 0.13 | 0.04 | 0.06 |
| *BnAMI15* | 0.46 | 0.22 | 0.23 | 0.08 | 0.0 |
| *BnAMI16* | 0.28 | 0.46 | 0.15 | 0.09 | 0.02 |
| *BnAMI17* | 0.7 | 0.05 | 0.08 | 0.02 | 0.15 |
| *BnAMI18* | 0.39 | 0.07 | 0.05 | 0.04 | 0.45 |
| *BnAMI19* | 0.24 | 0.4 | 0.14 | 0.21 | 0.01 |
| *BnAMI20* | 0.39 | 0.07 | 0.05 | 0.02 | 0.46 |
| *BnAMI21* | 0.07 | 0.03 | 0.72 | 0.17 | 0.01 |
| *BnAMI22* | 0.46 | 0.22 | 0.24 | 0.08 | 0.0 |
| *BnAMI23* | 0.59 | 0.17 | 0.19 | 0.05 | 0.01 |
| *BnAMI24* | 0.55 | 0.1 | 0.16 | 0.04 | 0.15 |
| *BnAMI25* | 0.24 | 0.15 | 0.52 | 0.06 | 0.01 |
| *BnAMI26* | 0.29 | 0.38 | 0.16 | 0.12 | 0.05 |
| *BnAMI27* | 0.05 | 0.02 | 0.49 | 0.44 | 0.01 |
| *BnAMI28* | 0.49 | 0.06 | 0.09 | 0.01 | 0.36 |
| *BnAMI29* | 0.06 | 0.01 | 0.55 | 0.37 | 0.01 |
| *BnAMI30* | 0.74 | 0.07 | 0.13 | 0.03 | 0.03 |
| *BnAMI31* | 0.02 | 0.12 | 0.69 | 0.17 | 0.0 |
| *BnAMI32* | 0.46 | 0.26 | 0.21 | 0.07 | 0.0 |
| *BnAMI33* | 0.58 | 0.18 | 0.19 | 0.05 | 0.01 |
| *BnAMI34* | 0.53 | 0.26 | 0.14 | 0.06 | 0.01 |
| *BnAMI35* | 0.59 | 0.2 | 0.12 | 0.08 | 0.01 |
| *BnAMI36* | 0.75 | 0.07 | 0.12 | 0.03 | 0.03 |
| *BnAMI37* | 0.07 | 0.86 | 0.06 | 0.01 | 0.0 |
| *BnAMI38* | 0.02 | 0.05 | 0.85 | 0.08 | 0.0 |
| *BnAMI39* | 0.55 | 0.07 | 0.08 | 0.01 | 0.29 |
| *BnAMI40* | 0.05 | 0.04 | 0.78 | 0.11 | 0.01 |
| *BnAMI41* | 0.61 | 0.21 | 0.13 | 0.05 | 0.01 |
| *BnAMI42* | 0.08 | 0.1 | 0.46 | 0.35 | 0.01 |
| *BnAMI43* | 0.75 | 0.07 | 0.12 | 0.03 | 0.03 |
| *BnAMI44* | 0.74 | 0.05 | 0.09 | 0.03 | 0.09 |
| *BnAMI45* | 0.61 | 0.05 | 0.1 | 0.02 | 0.22 |
| *BnAMI46* | 0.59 | 0.22 | 0.12 | 0.07 | 0.01 |
| *BnAMI47* | 0.05 | 0.01 | 0.62 | 0.31 | 0.01 |
| *BnAMI48* | 0.68 | 0.06 | 0.07 | 0.02 | 0.18 |
| *BnAMI49* | 0.57 | 0.08 | 0.06 | 0.05 | 0.24 |
| *BnAMI50* | 0.59 | 0.05 | 0.11 | 0.02 | 0.23 |
| *BnAMI51* | 0.44 | 0.07 | 0.04 | 0.01 | 0.44 |
| *BnAMI52* | 0.24 | 0.16 | 0.25 | 0.1 | 0.24 |
| *BnAMI53* | 0.07 | 0.1 | 0.47 | 0.34 | 0.01 |
| *BnAMI54* | 0.08 | 0.77 | 0.12 | 0.03 | 0.0 |
| *BnAMI55* | 0.35 | 0.17 | 0.41 | 0.06 | 0.01 |
| *BnAMI56* | 0.27 | 0.06 | 0.07 | 0.03 | 0.56 |
| *BnAMI57* | 0.03 | 0.04 | 0.87 | 0.05 | 0.0 |
| *BnAMI58* | 0.07 | 0.84 | 0.07 | 0.02 | 0.0 |
| *BnAMI59* | 0.06 | 0.01 | 0.64 | 0.29 | 0.01 |
| *BnAMI60* | 0.06 | 0.01 | 0.55 | 0.37 | 0.01 |
| *BnAMI61* | 0.73 | 0.05 | 0.09 | 0.02 | 0.11 |
| *BnAMI62* | 0.33 | 0.48 | 0.04 | 0.14 | 0.01 |
| *BnAMI63* | 0.61 | 0.21 | 0.11 | 0.07 | 0.01 |
| *BnAMI64* | 0.07 | 0.03 | 0.73 | 0.16 | 0.01 |
| *BnAMI65* | 0.75 | 0.07 | 0.12 | 0.03 | 0.03 |
| *BnAMI66* | 0.41 | 0.08 | 0.06 | 0.02 | 0.43 |
| *BnAMI67* | 0.24 | 0.29 | 0.24 | 0.19 | 0.04 |
| *BnAMI68* | 0.03 | 0.01 | 0.94 | 0.03 | 0.0 |
| *BnAMI69* | 0.46 | 0.19 | 0.29 | 0.05 | 0.01 |
| *BnAMI70* | 0.34 | 0.09 | 0.15 | 0.06 | 0.36 |
| *BnAMI71* | 0.29 | 0.47 | 0.12 | 0.08 | 0.04 |
| *BnAMI72* | 0.75 | 0.07 | 0.13 | 0.03 | 0.03 |
| *BnAMI73* | 0.61 | 0.09 | 0.21 | 0.04 | 0.06 |
| *BnAMI74* | 0.46 | 0.23 | 0.23 | 0.08 | 0.0 |
| *BnAMI75* | 0.7 | 0.05 | 0.08 | 0.02 | 0.14 |
| *BnAMI76* | 0.73 | 0.04 | 0.08 | 0.03 | 0.11 |
| *BnAMI77* | 0.57 | 0.06 | 0.1 | 0.02 | 0.26 |
| *BnAMI78* | 0.74 | 0.07 | 0.13 | 0.03 | 0.03 |
| *BnAMI79* | 0.02 | 0.09 | 0.7 | 0.19 | 0.0 |
| *BnAMI80* | 0.46 | 0.22 | 0.24 | 0.08 | 0.0 |
| *BnAMI81* | 0.02 | 0.04 | 0.88 | 0.05 | 0.0 |
| *BnAMI82* | 0.54 | 0.07 | 0.08 | 0.01 | 0.31 |
| *BnAMI83* | 0.06 | 0.04 | 0.75 | 0.13 | 0.01 |
| *BnAMI84* | 0.07 | 0.86 | 0.06 | 0.01 | 0.0 |
| *BnAMI85* | 0.46 | 0.26 | 0.21 | 0.07 | 0.0 |
| *BnAMI86* | 0.4 | 0.15 | 0.36 | 0.08 | 0.01 |
| *BnAMI87* | 0.5 | 0.24 | 0.18 | 0.07 | 0.01 |
| *BnAMI88* | 0.6 | 0.2 | 0.12 | 0.07 | 0.01 |
| *BnAMI89* | 0.3 | 0.54 | 0.1 | 0.05 | 0.02 |
| *BnAMI90* | 0.02 | 0.01 | 0.94 | 0.03 | 0.0 |
| *BnAMI91* | 0.44 | 0.07 | 0.04 | 0.01 | 0.44 |

**Table S3. Nonsynonymous (Ka) and synonymous (Ks) nucleotide substitution rates for *Arabidopsis thaliana* and *B. napus* *TAR*s protein-coding sequence.**

| ***A. thaliana* ID** | ***B. napus* gene** | ***B. napus* ID** | ***TAR* family** | **Ka** | **Ks** | **Ka/Ks** |
| --- | --- | --- | --- | --- | --- | --- |
| **ONE COPY LOCI** | | | | | | |
| AT1G34060 | *BnALL3* | BnaA08g06520D | Alliinase_C | 0.1080448 | 0.354725608 | 0.304586974 |
| **TWO-COPY LOCI** | | | | | | |
| AT1G01480 | *BnAMI63* | BnaC05g00530D | Aminotran_1_2 | 0.068046945 | 0.52795636 | 0.128887442 |
|  | *BnAMI41* | BnaA10g00470D | Aminotran_1_2 | 0.07915416 | 0.523171649 | 0.151296731 |
| AT1G34040 | *BnALL8* | BnaC06g07950D | Alliinase_C | 0.121150276 | 0.404677101 | 0.299375169 |
|  | *BnALL3* | BnaA08g06520D | Alliinase_C | 0.141530934 | 0.485951526 | 0.291244963 |
| AT2G22810 | *BnAMI19* | BnaA04g13310D | Aminotran_1_2 | 0.061674247 | 0.33995271 | 0.181420078 |
|  | *BnAMI62* | BnaC04g35390D | Aminotran_1_2 | 0.066058307 | 0.348829711 | 0.189371218 |
| AT2G24850 | *BnAMI38* | BnaA09g41170D | Aminotran_1_2 | 0.132629005 | 0.623437664 | 0.212738197 |
|  | *BnAMI11* | BnaA03g22790D | Aminotran_1_2 | 0.115943116 | 0.56951396 | 0.20358257 |
| AT2G24850 | *BnAMI57* | BnaC03g26850D | Aminotran_1_2 | 0.116282418 | 0.564381963 | 0.206034966 |
|  | *BnAMI81* | BnaC08g33730D | Aminotran_1_2 | 0.117012802 | 0.597785082 | 0.195743931 |
| AT4G08040 | *BnAMI58* | BnaC03g28000D | Aminotran_1_2 | 0.047715437 | 0.409026399 | 0.116656132 |
|  | *BnAMI12* | BnaA03g23600D | Aminotran_1_2 | 0.045727529 | 0.409026399 | 0.111796034 |
| AT5G49810 | *BnAMI8* | BnaA02g11830D | Aminotran_1_2 | 0.039002498 | 0.323553847 | 0.120544071 |
|  | *BnAMI54* | BnaC02g16260D | Aminotran_1_2 | 0.039651266 | 0.332854427 | 0.119124948 |
| AT5G51690 | *BnAMI53* | BnaC02g15560D | Aminotran_1_2 | 0.048133829 | 0.666522935 | 0.072216313 |
|  | *BnAMI42* | BnaAnng08090D | Aminotran_1_2 | 0.045714506 | 0.653636088 | 0.069938773 |
| AT5G65800 | *BnAMI85* | BnaC09g07070D | Aminotran_1_2 | 0.012326681 | 0.367027183 | 0.033585198 |
|  | *BnAMI32* | BnaA09g07210D | Aminotran_1_2 | 0.012326681 | 0.37818499 | 0.032594315 |
| **THREE-COPY LOCI** | | | | | | |
| AT1G23320 | *BnALL10* | BnaC05g18610D | Alliinase_C | 0.137440761 | 0.454561956 | 0.302358698 |
|  | *BnALL11* | BnaA09g31200D | Alliinase_C | 0.132470896 | 0.430956429 | 0.307388142 |
|  | *BnALL12* | BnaA09g31180D | Alliinase_C | 0.139450434 | 0.447977857 | 0.311288675 |
| AT1G62960 | *BnAMI87* | BnaC09g12920D | Aminotran_1_2 | 0.084130608 | 0.628055228 | 0.133954156 |
|  | *BnAMI34* | BnaA09g13010D | Aminotran_1_2 | 0.074754145 | 0.577823449 | 0.129371947 |
|  | *BnAMI6* | BnaA01g15380D | Aminotran_1_2 | 0.53400397 | NaN | NaN |
| AT1G77670 | *BnAMI70* | BnaC06g38270D | Aminotran_1_2 | 0.136617653 | 0.574315069 | 0.237879277 |
|  | *BnAMI90* | BnaCnng64190D | Aminotran_1_2 | 0.092229394 | 0.911920685 | 0.101137517 |
|  | *BnAMI68* | BnaC06g20760D | Aminotran_1_2 | 0.093938498 | 0.863442017 | 0.108795375 |
| AT2G13810 | *BnAMI14* | BnaA03g38470D | Aminotran_1_2 | 0.088509415 | 0.477191742 | 0.18547977 |
|  | *BnAMI59* | BnaC03g45280D | Aminotran_1_2 | 0.051667502 | 0.430800299 | 0.119933766 |
|  | *BnAMI13* | BnaA03g38440D | Aminotran_1_2 | 0.052714317 | 0.419425052 | 0.125682327 |
| AT3G61510 | *BnAMI37* | BnaA09g39260D | Aminotran_1_2 | 0.066155721 | 0.49433684 | 0.133827212 |
|  | *BnAMI41* | BnaA10g00470D | Aminotran_1_2 | 0.256197217 | NaN | NaN |
|  | *BnAMI84* | BnaC08g49010D | Aminotran_1_2 | 0.072123057 | 0.489299758 | 0.147400558 |
| AT4G11280 | *BnAMI88* | BnaC09g24050D | Aminotran_1_2 | 0.044010205 | 0.49647991 | 0.088644483 |
|  | *BnAMI41* | BnaA10g00470D | Aminotran_1_2 | 0.313383426 | NaN | NaN |
|  | *BnAMI35* | BnaA09g21860D | Aminotran_1_2 | 0.041895708 | 0.550460761 | 0.076110253 |
| AT4G24670 | *BnALL5* | BnaAnng22040D | Alliinase_C | 0.423571137 | NaN | NaN |
|  | *BnALL6* | BnaC01g16530D | Alliinase_C | 0.076664632 | 0.431776444 | 0.17755631 |
|  | *BnALL1* | BnaA01g14030D | Alliinase_C | 0.091361474 | 0.464520758 | 0.196678992 |
| AT4G28410 | *BnAMI18* | BnaA03g49250D | Aminotran_1_2 | 0.106521012 | 0.628467776 | 0.169493196 |
|  | *BnAMI77* | BnaC07g41280D | Aminotran_1_2 | 0.104066504 | 0.623024233 | 0.16703444 |
|  | *BnAMI76* | BnaC07g41260D | Aminotran_1_2 | 0.146097416 | 0.722761574 | 0.202137774 |
| AT5G36160 | *BnAMI20* | BnaA05g12790D | Aminotran_1_2 | 0.071813447 | 0.412520688 | 0.174084474 |
|  | *BnAMI39* | BnaA09g43670D | Aminotran_1_2 | 0.142521116 | 0.703779846 | 0.202508095 |
|  | *BnAMI82* | BnaC08g36300D | Aminotran_1_2 | 0.141568531 | 0.717303152 | 0.197362203 |
| **FOUR-COPY LOCI** | | | | | | |
| AT1G70560 | *BnALL5* | BnaAnng22040D | Alliinase_C | 0.06625698 | 0.550123857 | 0.120440113 |
|  | *BnALL2* | BnaA02g14990D | Alliinase_C | 0.050444103 | 0.42571794 | 0.118491842 |
|  | *BnALL7* | BnaC02g19980D | Alliinase_C | 0.047462871 | 0.455043679 | 0.104303989 |
|  | *BnALL9* | BnaC06g43720D | Alliinase_C | 0.047301137 | 0.498491419 | 0.094888567 |
| AT1G71920 | *BnAMI55* | BnaC02g20870D | Aminotran_1_2 | 0.031556913 | 0.422098875 | 0.074761898 |
|  | *BnAMI9* | BnaA02g15650D | Aminotran_1_2 | 0.03363345 | 0.437443699 | 0.076886351 |
|  | *BnAMI69* | BnaC06g32920D | Aminotran_1_2 | 0.046762538 | 0.591958207 | 0.078996351 |
|  | *BnAMI25* | BnaA07g29720D | Aminotran_1_2 | 0.056158124 | 0.560024651 | 0.10027795 |
| AT2G20610 | *BnAMI89* | BnaCnng43090D | Aminotran_1_2 | 0.063300314 | 0.470462137 | 0.134549221 |
|  | *BnAMI33* | BnaA09g10030D | Aminotran_1_2 | 0.071511123 | 0.449161859 | 0.159210141 |
|  | *BnAMI23* | BnaA07g00460D | Aminotran_1_2 | 0.057523391 | 0.461554961 | 0.124629558 |
|  | *BnAMI86* | BnaC09g10110D | Aminotran_1_2 | 0.070901465 | 0.454047685 | 0.156154226 |
| AT3G49700 | *BnAMI74* | BnaC07g31520D | Aminotran_1_2 | 0.013497106 | 0.271383364 | 0.049734465 |
|  | *BnAMI80* | BnaC08g21300D | Aminotran_1_2 | 0.018523748 | 0.307756169 | 0.060189688 |
|  | *BnAMI22* | BnaA06g15630D | Aminotran_1_2 | 0.017579833 | 0.303506648 | 0.0579224 |
|  | *BnAMI15* | BnaA03g40560D | Aminotran_1_2 | 0.010587057 | 0.311151396 | 0.034025421 |
| AT4G23600 | *BnAMI16* | BnaA03g46060D | Aminotran_1_2 | 0.163542642 | 0.773484073 | 0.21143634 |
|  | *BnAMI5* | BnaA01g13280D | Aminotran_1_2 | 0.117439791 | 0.497470663 | 0.236073803 |
|  | *BnAMI50* | BnaC01g15060D | Aminotran_1_2 | 0.113612784 | 0.533958129 | 0.212774707 |
|  | *BnAMI28* | BnaA08g10670D | Aminotran_1_2 | 0.176069338 | 1.172812296 | 0.150125761 |
| AT4G37770 | *BnAMI61* | BnaC03g76570D | Aminotran_1_2 | 0.04558318 | 0.422375343 | 0.107921025 |
|  | *BnAMI44* | BnaAnng30490D | Aminotran_1_2 | 0.044768164 | 0.411275017 | 0.108852135 |
|  | *BnAMI1* | BnaA01g00270D | Aminotran_1_2 | 0.047616735 | 0.428323662 | 0.111169985 |
|  | *BnAMI46* | BnaC01g01270D | Aminotran_1_2 | 0.043200087 | 0.434910497 | 0.099330982 |
| AT5G10330 | *BnAMI55* | BnaC02g20870D | Aminotran_1_2 | 0.031556913 | 0.434658038 | 0.072601702 |
|  | *BnAMI9* | BnaA02g15650D | Aminotran_1_2 | 0.03363345 | 0.450395991 | 0.074675286 |
|  | *BnAMI69* | BnaC06g32920D | Aminotran_1_2 | 0.046762538 | 0.599735334 | 0.077971958 |
|  | *BnAMI25* | BnaA07g29720D | Aminotran_1_2 | 0.056158124 | 0.567395718 | 0.098975234 |
| AT5G53970 | *BnAMI52* | BnaC02g13860D | Aminotran_1_2 | 0.032145832 | 0.333209859 | 0.096473231 |
|  | *BnAMI82* | BnaC08g36300D | Aminotran_1_2 | 0.399191584 | 2.453410012 | 0.162708875 |
|  | *BnAMI7* | BnaA02g09960D | Aminotran_1_2 | 0.028815721 | 0.36542709 | 0.078854913 |
|  | *BnAMI39* | BnaA09g43670D | Aminotran_1_2 | 0.40409433 | 2.604713001 | 0.155139676 |
| **FIVE-COPY LOCI** | | | | | | |
| AT4G23590 | *BnAMI5* | BnaA01g13280D | Aminotran_1_2 | 0.148576748 | 0.684203402 | 0.217152892 |
|  | *BnAMI16* | BnaA03g46060D | Aminotran_1_2 | 0.162144642 | 0.733418 | 0.221080805 |
|  | *BnAMI4* | BnaA01g13270D | Aminotran_1_2 | 0.162101191 | 0.721744336 | 0.224596416 |
|  | *BnAMI45* | BnaAnng31450D | Aminotran_1_2 | 0.16032074 | 0.726972633 | 0.220532015 |
|  | *BnAMI50* | BnaC01g15060D | Aminotran_1_2 | 0.147087234 | 0.706267741 | 0.208259878 |
| AT4G26200 | *BnAMI51* | BnaC01g18250D | Aminotran_1_2 | 0.019658755 | 0.444232025 | 0.04425335 |
|  | *BnAMI66* | BnaCnng71530D | Aminotran_1_2 | 0.021660378 | 0.455306503 | 0.04757318 |
|  | *BnAMI75* | BnaC07g40130D | Aminotran_1_2 | 0.039468566 | 0.415797654 | 0.094922531 |
|  | *BnAMI6* | BnaA01g15380D | Aminotran_1_2 | 0.022646945 | 0.41609156 | 0.054427793 |
|  | *BnAMI17* | BnaA03g47880D | Aminotran_1_2 | 0.03727784 | 0.430582333 | 0.086575405 |
| AT4G28420 | *BnAMI86* | BnaC09g10110D | Aminotran_1_2 | 0.171110324 | 1.069853058 | 0.159938155 |
|  | *BnAMI3* | BnaA01g08470D | Aminotran_1_2 | 0.08793787 | 0.39843417 | 0.220708655 |
|  | *BnAMI49* | BnaC01g10040D | Aminotran_1_2 | 0.095011712 | 0.460667104 | 0.206248094 |
|  | *BnAMI33* | BnaA09g10030D | Aminotran_1_2 | 0.170885546 | 1.040327322 | 0.164261327 |
|  | *BnAMI48* | BnaC01g10020D | Aminotran_1_2 | 0.088910798 | 0.425893564 | 0.208762953 |
| AT4G33680 | *BnAMI14* | BnaA03g38470D | Aminotran_1_2 | 0.319938711 | 2.770211294 | 0.11549253 |
|  | *BnAMI47* | BnaC01g04560D | Aminotran_1_2 | 0.02641721 | 0.407438755 | 0.064837254 |
|  | *BnAMI2* | BnaA01g03290D | Aminotran_1_2 | 0.022451652 | 0.401717318 | 0.055889182 |
|  | *BnAMI29* | BnaA08g11470D | Aminotran_1_2 | 0.022252519 | 0.387121896 | 0.057481944 |
|  | *BnAMI60* | BnaC03g66430D | Aminotran_1_2 | 0.022254313 | 0.352689824 | 0.063098825 |
| **SIX-COPY LOCI** | | | | | | |
| AT1G17290 | *BnAMI31* | BnaA08g23190D | Aminotran_1_2 | 0.061651066 | 0.391900758 | 0.157312955 |
|  | *BnAMI64* | BnaC05g13450D | Aminotran_1_2 | 0.057093922 | 0.402886427 | 0.1417122 |
|  | *BnAMI79* | BnaC08g17310D | Aminotran_1_2 | 0.055155658 | 0.436569248 | 0.126338852 |
|  | *BnAMI21* | BnaA06g11610D | Aminotran_1_2 | 0.059219023 | 0.407529656 | 0.145312179 |
|  | *BnAMI83* | BnaC08g37860D | Aminotran_1_2 | 0.04496976 | 0.447347632 | 0.100525312 |
|  | *BnAMI40* | BnaA09g45030D | Aminotran_1_2 | 0.048222729 | 0.473841363 | 0.101769775 |
| AT1G70580 | *BnAMI43* | BnaAnng22050D | Aminotran_1_2 | 0.020637193 | 0.409235013 | 0.050428708 |
|  | *BnAMI78* | BnaC08g06270D | Aminotran_1_2 | 0.043769088 | 0.983536073 | 0.044501762 |
|  | *BnAMI24* | BnaA07g10020D | Aminotran_1_2 | 0.039387863 | 0.721227779 | 0.054612238 |
|  | *BnAMI65* | BnaC05g18600D | Aminotran_1_2 | 0.033038841 | 0.777746333 | 0.042480227 |
|  | *BnAMI36* | BnaA09g31220D | Aminotran_1_2 | 0.033972461 | 0.739049306 | 0.045967787 |
|  | *BnAMI72* | BnaC06g43710D | Aminotran_1_2 | 0.016009146 | 0.398303938 | 0.04019329 |
| AT1G72330 | *BnAMI21* | BnaA06g11610D | Aminotran_1_2 | 0.084113174 | 0.730828535 | 0.115092897 |
|  | *BnAMI83* | BnaC08g37860D | Aminotran_1_2 | 0.079476637 | 0.79426185 | 0.100063521 |
|  | *BnAMI40* | BnaA09g45030D | Aminotran_1_2 | 0.081443585 | 0.85007468 | 0.095807565 |
|  | *BnAMI64* | BnaC05g13450D | Aminotran_1_2 | 0.07939009 | 0.707424961 | 0.112224044 |
|  | *BnAMI31* | BnaA08g23190D | Aminotran_1_2 | 0.078952326 | 0.700135127 | 0.112767268 |
|  | *BnAMI79* | BnaC08g17310D | Aminotran_1_2 | 0.073223606 | 0.747113031 | 0.09800874 |
| AT1G80360 | *BnAMI71* | BnaC06g40630D | Aminotran_1_2 | 0.062096338 | 0.356884429 | 0.173995651 |
|  | *BnAMI10* | BnaA02g19790D | Aminotran_1_2 | 0.06003781 | 0.364214348 | 0.164841968 |
|  | *BnAMI27* | BnaA07g38380D | Aminotran_1_2 | 0.086897421 | 0.329097392 | 0.264047734 |
|  | *BnAMI67* | BnaC06g19110D | Aminotran_1_2 | 0.081914606 | 0.340298278 | 0.240714136 |
|  | *BnAMI26* | BnaA07g35690D | Aminotran_1_2 | 0.063517788 | 0.385063774 | 0.164953942 |
|  | *BnAMI56* | BnaC02g23160D | Aminotran_1_2 | 0.059991609 | 0.365331242 | 0.164211548 |
| **EIGHT-COPY LOCI** | | | | | | |
| AT1G23310 | *BnAMI30* | BnaA08g20540D | Aminotran_1_2 | 0.023169655 | 0.258662973 | 0.089574687 |
|  | *BnAMI43* | BnaAnng22050D | Aminotran_1_2 | 0.045312658 | 0.634004247 | 0.07147059 |
|  | *BnAMI65* | BnaC05g18600D | Aminotran_1_2 | 0.0259927 | 0.258891461 | 0.100399991 |
|  | *BnAMI36* | BnaA09g31220D | Aminotran_1_2 | 0.02692329 | 0.255249361 | 0.105478384 |
|  | *BnAMI73* | BnaC07g13140D | Aminotran_1_2 | 0.041249966 | 0.229613941 | 0.179649224 |
|  | *BnAMI78* | BnaC08g06270D | Aminotran_1_2 | 0.023214726 | 0.302106241 | 0.076842921 |
|  | *BnAMI72* | BnaC06g43710D | Aminotran_1_2 | 0.04244876 | 0.632542273 | 0.067108179 |
|  | *BnAMI24* | BnaA07g10020D | Aminotran_1_2 | 0.023085087 | 0.23237721 | 0.099343163 |

**Table S4. the pyridoxal binding sites of the *BnALL10*, *BnAMI78*,2HOX and 3TCM:** The binding site consists of the sequence number and abbreviation of the amino acid residue in the protein

| **Name** | **Catalytic Residue** | **Binding Site1** | **Binding Site2** | **Binding Site3** | **Binding Site4** | **Binding Site5** | **Binding Site6** | **Binding Site7** | **Binding Site8** | **Binding Site9** | **Binding Site10** |
| --- | --- | --- | --- | --- | --- | --- | --- | --- | --- | --- | --- |
| *BnALL10* | 209K | 95G | 96S | 97S | 125Y | 164N | 186Y | 206T | 208S | 209K | 217R |
| *BnAMI78* | 213K | 60G | 61A | 62S | 87Y | 143N | 174Y | 210T | 212S | 213K | 222R |
| 2HOX | 289K | 169G | 170V | 171T | 203Y | 245N | 266Y | 286T | 288S | 289K | 297R |
| 3TCM | 317K | 165G | 166A | 167S | 192Y | 248N | 279Y | 314S | 316S | 317K | 326R |

**Table S5. the distance from pyridoxal binding sites to catalytic residue of the *BnALL10*, *BnAMI78*,2HOX and 3TCM:** Each distance is the distance from the side chain group of the amino acid residue at the binding site to the side chain group of the catalytic residue lysine

| **Catalytic Residue of BnALL10:209K** | | | | | | | | | | |
| --- | --- | --- | --- | --- | --- | --- | --- | --- | --- | --- |
| **Binding Site** | 95G | 96S | 97S | 125Y | 164N | 186Y | 206T | 208S | 209K | 217R |
| **Distance(nm)** | 12.4 | 8.8 | 9.4 | 7.4 | 6.1 | 2.7 | 7.2 | 5 | 0 | 13.1 |
| **Catalytic Residue of BnAMI78:213K** | | | | | | | | | | |
| **Binding Site** | 60G | 61A | 62S | 87Y | 143N | 174Y | 210T | 212S | 213K | 222R |
| **Distance(nm)** | 12.6 | 9.4 | 9.8 | 6.6 | 4.4 | 2.3 | 6.8 | 7.2 | 0 | 14.5 |
| **Catalytic Residue of 2HOX:289K** | | | | | | | | | | |
| **Binding Site** | 169G | 170V | 171T | 203Y | 245N | 266Y | 286T | 288S | 289K | 297R |
| **Distance(nm)** | 13.1 | 10.7 | 10.3 | 8.1 | 6 | 3.2 | 8.8 | 7.1 | 0 | 12.8 |
| **Catalytic Residue of 3TCM:317K** | | | | | | | | | | |
| **Binding Site** | 165G | 166A | 167S | 192Y | 248N | 279Y | 314S | 316S | 317K | 326R |
| **Distance(nm)** | 12.6 | 9.4 | 9.8 | 6.6 | 4.4 | 2.3 | 7.9 | 5.4 | 0 | 14.5 |

**Table S6. Primer pairs used in qRT-PCR**

| Gene name | Primer-F(5'-3') | Primer-R(5'-3') |
| --- | --- | --- |
| *BnAMI85* | AACGTGAAACTAAACATCTCGC | TCTTGATATCATCCGTCCACAG |
| *BnAMI68* | GGTCTTGTTATTGACCCTGAGA | CCTCGTAGGAATCGTAGAAAGG |
| *BnALL2* | CGTACGAAGAATACTGGAGGAA | CACGTCGCTGAAATAACTCATT |
| *BnAMI57* | CTTCCTACTAAGCTAAGACCGG | CCTGCGAGAGAATCTATTACGA |
| *BnAMI47* | CTTGTTTCAACGGTGCATCTAA | TCATACCCGAGAGAAGTGAATG |
| *BnAMI55* | GATTTCAGCTTGAACGTAGACC | TCCATCTGGATTGTTTGGAGAA |
| *BnAMI18* | AAAGAAGTTGAACGAAAGACCG | TTTGCTGCAGTTCTCGAATAAC |
| *BnAMI24* | AGAAGAGATGCCTGAGATCATG | GAAAGACAAGTGTACGACATCG |
| *BnAMI14* | GACTCCACAAACAATCTTGCTT | AGCCCATACCCTCTATATCCTT |
| *BnAMI69* | AAGTGAAACATCTCTGGTAGCA | GTCACTTTCAATGGGCTACATC |
| *BnAMI54* | TCTTCTTGTCGGACCTTTACAA | CTTCCTTGACTGAAAACCTTCG |
| *BnAMI9* | GAAGGAGAAGTCTTTGGAGTGA | GCTCTCGTCGATTAACAAAGAC |
| *BnAMI90* | TGAAGCTTAAGAGAGTGGTCTG | GAACCGAAGAAGAACAACAACA |
| *BnAMI27* | TAAAACCTATGGCATGATGGGT | ACCTTCCTCTAAAGCGTGTAAA |
| *BnAMI37* | TCTCAGACACAGAGCCTTTTAG | ATCAAGTGTCTCTTCGCTACTC |
| *BnAMI53* | CGGTATCATAGGATTAGGCTGG | GTTAAGCTGGTTACATCCGTTC |
| *BnAMI71* | GGTTTAAAACATTGTTGCGTGG | CCTTCCTCTAGTGCGTATACAG |
| *BnAMI70* | GGCGTTGTTTTCGTCTTATGTA | CGGCATTGGAAAAGATAATCCG |
| *BnALL4* | ACAAGCTAAGCGGTCATTCTTA | ATGATCGACACTGTAAGAAGCA |

**Table S7. Original data of qRT-PCR**

| Gene name |  | Stem | Root | Leaf | Flower | Early_seeds | Late_seeds |
| --- | --- | --- | --- | --- | --- | --- | --- |
| *BnALL2* |  | 2.169055 | -0.66394 | 0.463761 | 0.859691 | -0.5697 | 1.459021 |
| *BnALL4* |  | 1.28215 | -0.44091 | -0.29945 | -0.94779 | -1.11226 | -1.05071 |
| *BnAMI9* |  | 0.602528 | 0.160676 | 1.331245 | 0.333474 | 3.606718 | 1.523586 |
| *BnAMI14* |  | -1.33216 | -0.42346 | 1.763064 | 1.870483 | 1.772409 | 1.973943 |
| *BnAMI18* |  | 1.687896 | 0.437926 | -0.46853 | 1.010343 | -0.88088 | -1.16847 |
| *BnAMI24* |  | 1.73248 | 1.656284 | 1.504681 | 1.168706 | 0.890134 | 1.092982 |
| *BnAMI27* |  | -1.21359 | -0.08593 | 1.423592 | 0.284345 | 4.229844 | 2.075327 |
| *BnAMI37* |  | -0.63903 | -1.36982 | 1.140845 | 0.785 | 0.590479 | 0.740505 |
| *BnAMI47* |  | -0.86957 | -0.98788 | -1.00862 | -0.17908 | 0.367425 | 0.176457 |
| *BnAMI53* |  | 0.896468 | 1.088564 | -2.20891 | -2.32013 | -2.60541 | -2.43867 |
| *BnAMI54* |  | 1.779149 | 1.762706 | -0.32235 | 2.799805 | 2.541449 | 2.908547 |
| *BnAMI55* |  | 0.516174 | 0.202673 | 0.352807 | 0.556846 | 0.798674 | 0.363989 |
| *BnAMI57* |  | -0.98127 | -0.04087 | -1.19451 | -0.59582 | -0.75992 | -0.89645 |
| *BnAMI68* |  | 0.617642 | -0.20759 | 1.948838 | 1.851415 | 1.536781 | 1.240146 |
| *BnAMI69* |  | 0.987377 | 1.007508 | 0.92421 | 1.870483 | 1.038264 | 0.500581 |
| *BnAMI70* |  | -0.06602 | 0.116494 | -0.41408 | -0.27064 | -0.70117 | -1.09068 |
| *BnAMI71* |  | 1.393448 | 1.977274 | -0.06517 | -0.28493 | -0.04335 | -0.19433 |
| *BnAMI85* |  | 0.399327 | -0.13049 | -0.46719 | -1.09589 | -0.48837 | -0.52599 |
| *BnAMI90* |  | 1.956441 | 1.953948 | 1.423592 | 0.284345 | 4.229844 | 2.075327 |

**Table S8. Correspondence between physical and genetic distances of each chromosome in *B.napus***

| **Chr.** | **Genetic distance** | **Physical location** |
| --- | --- | --- |
| chrA01 | 7.732 | 586111 |
| chrA01 | 8.022 | 830140 |
| chrA01 | 8.188 | 695538 |
| chrA01 | 8.884 | 925718 |
| chrA01 | 9.964 | 973485 |
| chrA01 | 10.102 | 974259 |
| chrA01 | 10.789 | 987839 |
| chrA01 | 14.58 | 1145107 |
| chrA01 | 14.752 | 1230229 |
| chrA01 | 15.91 | 1508664 |
| chrA01 | 16.714 | 1472184 |
| chrA01 | 16.807 | 1559229 |
| chrA01 | 22.949 | 2883551 |
| chrA01 | 26.146 | 2814454 |
| chrA01 | 26.764 | 2990638 |
| chrA01 | 26.764 | 2943918 |
| chrA01 | 26.86 | 3000120 |
| chrA01 | 29.579 | 3788535 |
| chrA01 | 30.611 | 3311218 |
| chrA01 | 31.939 | 3391055 |
| chrA01 | 32.681 | 3533983 |
| chrA01 | 33.137 | 3748419 |
| chrA01 | 33.256 | 3794167 |
| chrA01 | 33.434 | 3829340 |
| chrA01 | 33.434 | 3822757 |
| chrA01 | 33.434 | 3817699 |
| chrA01 | 34.244 | 3751354 |
| chrA01 | 34.482 | 3828756 |
| chrA01 | 34.895 | 3894078 |
| chrA01 | 35.913 | 3956210 |
| chrA01 | 36.673 | 4227238 |
| chrA01 | 37.259 | 4340292 |
| chrA01 | 38.201 | 4515924 |
| chrA01 | 38.566 | 4517654 |
| chrA01 | 42.239 | 4829792 |
| chrA01 | 42.388 | 4892639 |
| chrA01 | 43.338 | 5043145 |
| chrA01 | 44.032 | 5214135 |
| chrA01 | 44.489 | 5394045 |
| chrA01 | 45.01 | 5473103 |
| chrA01 | 45.22 | 5651491 |
| chrA01 | 45.375 | 5561843 |
| chrA01 | 45.493 | 5685537 |
| chrA01 | 45.699 | 5698647 |
| chrA01 | 45.953 | 5739876 |
| chrA01 | 46.166 | 5956220 |
| chrA01 | 46.333 | 5834397 |
| chrA01 | 46.536 | 5927736 |
| chrA01 | 46.897 | 6016944 |
| chrA01 | 48.213 | 6120112 |
| chrA01 | 48.296 | 6472920 |
| chrA01 | 48.482 | 6325713 |
| chrA01 | 49.383 | 6774892 |
| chrA01 | 49.721 | 6849840 |
| chrA01 | 50.068 | 6966590 |
| chrA01 | 51.068 | 7014316 |
| chrA01 | 51.604 | 7048990 |
| chrA01 | 52.423 | 7087623 |
| chrA01 | 57.862 | 7913162 |
| chrA01 | 58.696 | 8432772 |
| chrA01 | 58.836 | 8053754 |
| chrA01 | 58.985 | 7983934 |
| chrA01 | 64.09 | 10725551 |
| chrA01 | 64.505 | 12892795 |
| chrA01 | 64.608 | 14963037 |
| chrA01 | 64.753 | 10746022 |
| chrA01 | 64.818 | 10904939 |
| chrA01 | 65.037 | 14133631 |
| chrA01 | 65.08 | 14134145 |
| chrA01 | 65.226 | 14134225 |
| chrA01 | 65.304 | 14041048 |
| chrA01 | 65.603 | 15454531 |
| chrA01 | 65.671 | 15527482 |
| chrA01 | 66.108 | 16421887 |
| chrA01 | 67.353 | 16946615 |
| chrA01 | 67.507 | 17545460 |
| chrA01 | 68.036 | 17482637 |
| chrA01 | 68.181 | 17695020 |
| chrA01 | 68.537 | 17693691 |
| chrA01 | 70.095 | 18103706 |
| chrA01 | 70.248 | 18058392 |
| chrA01 | 70.687 | 18057537 |
| chrA01 | 71.455 | 18231993 |
| chrA01 | 71.58 | 18237924 |
| chrA01 | 71.809 | 18510623 |
| chrA01 | 72.293 | 18222390 |
| chrA01 | 72.39 | 18221525 |
| chrA01 | 72.519 | 18426959 |
| chrA01 | 72.597 | 18223338 |
| chrA01 | 72.791 | 18312379 |
| chrA01 | 73.378 | 18222560 |
| chrA01 | 73.811 | 18221655 |
| chrA01 | 73.854 | 18222527 |
| chrA01 | 75.101 | 18649428 |
| chrA01 | 85.833 | 20622293 |
| chrA01 | 86.225 | 20658473 |
| chrA01 | 86.468 | 20676854 |
| chrA01 | 87.215 | 20892890 |
| chrA01 | 87.402 | 20910943 |
| chrA01 | 87.598 | 20833454 |
| chrA01 | 90.197 | 21006173 |
| chrA01 | 90.351 | 20944361 |
| chrA01 | 91.322 | 20969396 |
| chrA01 | 91.402 | 20942421 |
| chrA01 | 91.553 | 21039700 |
| chrA01 | 92.427 | 21236239 |
| chrA01 | 96.228 | 21522152 |
| chrA01 | 96.228 | 21521025 |
| chrA01 | 96.228 | 21515137 |
| chrA01 | 96.228 | 21511466 |
| chrA01 | 96.359 | 21468618 |
| chrA01 | 96.499 | 21466170 |
| chrA01 | 97.158 | 21708946 |
| chrA01 | 103.286 | 22656024 |
| chrA01 | 105.668 | 22733587 |
| chrA01 | 106.795 | 22379973 |
| chrA01 | 107.117 | 22575827 |
| chrA01 | 107.625 | 22380506 |
| chrA10 | 1.872 | 1556662 |
| chrA10 | 3.007 | 496890 |
| chrA10 | 3.211 | 497208 |
| chrA10 | 3.33 | 753140 |
| chrA10 | 3.487 | 467935 |
| chrA10 | 3.876 | 797977 |
| chrA10 | 4.299 | 821198 |
| chrA10 | 4.478 | 771130 |
| chrA10 | 5.752 | 6666673 |
| chrA10 | 6.304 | 924617 |
| chrA10 | 6.304 | 922093 |
| chrA10 | 6.542 | 1181976 |
| chrA10 | 6.897 | 1239055 |
| chrA10 | 7.683 | 1361192 |
| chrA10 | 7.908 | 1381506 |
| chrA10 | 9.044 | 1394759 |
| chrA10 | 10.671 | 1556940 |
| chrA10 | 10.955 | 1570259 |
| chrA10 | 11.104 | 1569846 |
| chrA10 | 11.364 | 4355308 |
| chrA10 | 11.407 | 4528050 |
| chrA10 | 11.548 | 2209080 |
| chrA10 | 11.548 | 2205779 |
| chrA10 | 11.959 | 4370370 |
| chrA10 | 12.012 | 3410726 |
| chrA10 | 12.163 | 7777496 |
| chrA10 | 12.234 | 5433960 |
| chrA10 | 12.481 | 6039114 |
| chrA10 | 12.638 | 2416799 |
| chrA10 | 13.15 | 8228929 |
| chrA10 | 13.382 | 8323703 |
| chrA10 | 13.919 | 8786714 |
| chrA10 | 14.102 | 9309696 |
| chrA10 | 14.254 | 9283405 |
| chrA10 | 14.465 | 9394078 |
| chrA10 | 14.684 | 9466108 |
| chrA10 | 15.141 | 9857660 |
| chrA10 | 15.292 | 9781474 |
| chrA10 | 15.433 | 10193878 |
| chrA10 | 15.959 | 10269655 |
| chrA10 | 16.073 | 10453145 |
| chrA10 | 16.156 | 10408013 |
| chrA10 | 16.51 | 10686495 |
| chrA10 | 16.56 | 10788870 |
| chrA10 | 16.838 | 10898442 |
| chrA10 | 17.31 | 11025866 |
| chrA10 | 17.774 | 11161855 |
| chrA10 | 18.786 | 11631962 |
| chrA10 | 19.199 | 11813082 |
| chrA10 | 19.644 | 11866078 |
| chrA10 | 22.234 | 12013420 |
| chrA10 | 22.447 | 12076521 |
| chrA10 | 22.696 | 12190371 |
| chrA10 | 23.155 | 12339619 |
| chrA10 | 23.346 | 12342569 |
| chrA10 | 23.608 | 12388279 |
| chrA10 | 23.95 | 12399153 |
| chrA10 | 24.209 | 12436785 |
| chrA10 | 24.89 | 12518833 |
| chrA10 | 25.158 | 12562239 |
| chrA10 | 28.544 | 12736401 |
| chrA10 | 28.771 | 12746998 |
| chrA10 | 29.474 | 12809792 |
| chrA10 | 29.707 | 12865752 |
| chrA10 | 30.074 | 12908565 |
| chrA10 | 30.76 | 13371778 |
| chrA10 | 31.713 | 13029716 |
| chrA10 | 32.93 | 13177259 |
| chrA10 | 33.519 | 13349570 |
| chrA10 | 33.893 | 13407775 |
| chrA10 | 34.181 | 13415801 |
| chrA10 | 34.966 | 13492437 |
| chrA10 | 35.733 | 13564470 |
| chrA10 | 37.392 | 13571043 |
| chrA10 | 38.25 | 13915902 |
| chrA10 | 39.405 | 13814477 |
| chrA10 | 40.245 | 13847692 |
| chrA10 | 43.012 | 14095811 |
| chrA10 | 43.749 | 14101730 |
| chrA10 | 45.1 | 14820985 |
| chrA10 | 47.187 | 14250328 |
| chrA10 | 47.793 | 14295791 |
| chrA10 | 49.603 | 14347540 |
| chrA10 | 50.103 | 14590015 |
| chrA10 | 50.514 | 14735386 |
| chrA10 | 51.2 | 14762090 |
| chrA10 | 51.542 | 14764744 |
| chrA10 | 52.906 | 15071972 |
| chrA10 | 53.933 | 15191391 |
| chrA10 | 54.258 | 15196681 |
| chrA10 | 54.966 | 15226007 |
| chrA10 | 54.966 | 15223379 |
| chrA10 | 56.475 | 15618151 |
| chrA10 | 57.434 | 15814975 |
| chrA10 | 57.609 | 15807427 |
| chrA10 | 58.458 | 15927704 |
| chrA10 | 62.861 | 16163197 |
| chrA10 | 63.152 | 16167528 |
| chrA10 | 63.507 | 16179307 |
| chrA10 | 64.244 | 16300632 |
| chrA10 | 64.83 | 16421861 |
| chrA10 | 65.218 | 16485715 |
| chrA10 | 65.498 | 16535674 |
| chrA10 | 65.771 | 16957929 |
| chrA10 | 66.045 | 17364040 |
| chrA10 | 66.231 | 17299351 |
| chrA10 | 66.447 | 17379781 |
| chrA02 | 14.662 | 258935 |
| chrA02 | 15.946 | 263236 |
| chrA02 | 16.112 | 349232 |
| chrA02 | 16.411 | 362797 |
| chrA02 | 16.555 | 393885 |
| chrA02 | 19.244 | 571429 |
| chrA02 | 20.174 | 575464 |
| chrA02 | 21.17 | 592213 |
| chrA02 | 21.579 | 605722 |
| chrA02 | 21.745 | 605761 |
| chrA02 | 22.909 | 715891 |
| chrA02 | 23.091 | 695258 |
| chrA02 | 23.386 | 651323 |
| chrA02 | 24.108 | 764055 |
| chrA02 | 24.287 | 786143 |
| chrA02 | 24.61 | 787577 |
| chrA02 | 25.793 | 849056 |
| chrA02 | 25.951 | 839226 |
| chrA02 | 26.5 | 965823 |
| chrA02 | 26.647 | 917116 |
| chrA02 | 28.914 | 1007266 |
| chrA02 | 32.273 | 1340342 |
| chrA02 | 32.622 | 1397553 |
| chrA02 | 33.844 | 1479886 |
| chrA02 | 33.986 | 1480317 |
| chrA02 | 34.284 | 1585327 |
| chrA02 | 34.444 | 1722448 |
| chrA02 | 35.039 | 1575719 |
| chrA02 | 39.725 | 1895619 |
| chrA02 | 42.705 | 2155959 |
| chrA02 | 43.317 | 2326044 |
| chrA02 | 44.068 | 2557301 |
| chrA02 | 44.688 | 2783793 |
| chrA02 | 45.139 | 3017576 |
| chrA02 | 45.139 | 3002716 |
| chrA02 | 45.485 | 3042625 |
| chrA02 | 46.186 | 3129377 |
| chrA02 | 46.186 | 3121259 |
| chrA02 | 52.72 | 20652843 |
| chrA02 | 52.72 | 4707978 |
| chrA02 | 52.958 | 4651803 |
| chrA02 | 53.298 | 4810435 |
| chrA02 | 54.538 | 4916246 |
| chrA02 | 54.907 | 4867664 |
| chrA02 | 55.815 | 5233185 |
| chrA02 | 56.138 | 4952402 |
| chrA02 | 56.489 | 5385009 |
| chrA02 | 56.612 | 5523108 |
| chrA02 | 57.051 | 5588399 |
| chrA02 | 57.309 | 5516541 |
| chrA02 | 58.77 | 5845639 |
| chrA02 | 58.77 | 5845572 |
| chrA02 | 59.243 | 6144790 |
| chrA02 | 59.49 | 6088989 |
| chrA02 | 59.766 | 6163063 |
| chrA02 | 59.853 | 6241401 |
| chrA02 | 60.128 | 6320447 |
| chrA02 | 60.389 | 6233151 |
| chrA02 | 60.53 | 6483784 |
| chrA02 | 60.731 | 6286849 |
| chrA02 | 60.991 | 6511465 |
| chrA02 | 61.126 | 6525252 |
| chrA02 | 61.269 | 6531335 |
| chrA02 | 62.281 | 6698315 |
| chrA02 | 62.611 | 7113848 |
| chrA02 | 62.945 | 7453498 |
| chrA02 | 63.833 | 8466731 |
| chrA02 | 64.057 | 7974470 |
| chrA02 | 64.237 | 8616203 |
| chrA02 | 64.397 | 7660190 |
| chrA02 | 64.47 | 8016184 |
| chrA02 | 64.696 | 9486416 |
| chrA02 | 65.067 | 9572811 |
| chrA02 | 65.344 | 7642607 |
| chrA02 | 65.679 | 9656681 |
| chrA02 | 65.764 | 9795046 |
| chrA02 | 67.226 | 6319890 |
| chrA02 | 76.948 | 19738670 |
| chrA02 | 77.641 | 20276725 |
| chrA02 | 77.916 | 20469259 |
| chrA02 | 78.02 | 20457747 |
| chrA02 | 78.3 | 20469483 |
| chrA02 | 78.773 | 20535947 |
| chrA02 | 79.125 | 20792994 |
| chrA02 | 79.385 | 20766917 |
| chrA02 | 79.969 | 20736911 |
| chrA02 | 83.741 | 21870390 |
| chrA02 | 84.905 | 21917951 |
| chrA02 | 85.064 | 21917490 |
| chrA02 | 85.24 | 21917889 |
| chrA02 | 85.39 | 22014506 |
| chrA02 | 85.633 | 21884005 |
| chrA02 | 88.244 | 22053784 |
| chrA02 | 89.446 | 22090336 |
| chrA02 | 95.362 | 22794357 |
| chrA02 | 95.362 | 3588900 |
| chrA02 | 95.673 | 3586484 |
| chrA02 | 96.075 | 22809070 |
| chrA02 | 96.217 | 22935785 |
| chrA02 | 96.217 | 22940898 |
| chrA02 | 96.217 | 22947031 |
| chrA02 | 96.284 | 22809570 |
| chrA02 | 96.36 | 22899483 |
| chrA02 | 96.404 | 22797918 |
| chrA02 | 96.537 | 22915311 |
| chrA02 | 96.644 | 22665232 |
| chrA02 | 96.712 | 22675994 |
| chrA02 | 97.285 | 22654444 |
| chrA02 | 97.626 | 23002881 |
| chrA02 | 97.892 | 22968983 |
| chrA02 | 105.117 | 23778711 |
| chrA02 | 105.824 | 23874281 |
| chrA02 | 108.335 | 24210540 |
| chrA02 | 108.572 | 24303175 |
| chrA02 | 108.698 | 24243857 |
| chrA02 | 109.431 | 24342829 |
| chrA02 | 109.634 | 24456151 |
| chrA02 | 109.814 | 24446290 |
| chrA02 | 109.961 | 24487935 |
| chrA02 | 110.227 | 24541079 |
| chrA02 | 110.326 | 24526446 |
| chrA02 | 110.66 | 24507500 |
| chrA02 | 110.874 | 24791537 |
| chrA03 | 6.587 | 1215973 |
| chrA03 | 13.859 | 1484621 |
| chrA03 | 14.284 | 1478354 |
| chrA03 | 14.64 | 1586644 |
| chrA03 | 15.425 | 1672709 |
| chrA03 | 15.861 | 1690009 |
| chrA03 | 16.682 | 1777276 |
| chrA03 | 17.071 | 1791402 |
| chrA03 | 17.396 | 1963359 |
| chrA03 | 17.724 | 1916234 |
| chrA03 | 17.853 | 2006985 |
| chrA03 | 18.293 | 2019534 |
| chrA03 | 18.912 | 2036063 |
| chrA03 | 19.181 | 2086264 |
| chrA03 | 19.566 | 2149196 |
| chrA03 | 20.366 | 2159110 |
| chrA03 | 20.939 | 2190835 |
| chrA03 | 21.444 | 2235893 |
| chrA03 | 21.545 | 2218163 |
| chrA03 | 22.194 | 2282944 |
| chrA03 | 22.599 | 2424926 |
| chrA03 | 24.989 | 2537900 |
| chrA03 | 26.936 | 2702453 |
| chrA03 | 27.431 | 2696331 |
| chrA03 | 27.624 | 2719127 |
| chrA03 | 28.311 | 2812412 |
| chrA03 | 28.438 | 2789426 |
| chrA03 | 28.644 | 2836271 |
| chrA03 | 29.239 | 2962344 |
| chrA03 | 29.46 | 3073692 |
| chrA03 | 30.761 | 3824337 |
| chrA03 | 31.795 | 3356534 |
| chrA03 | 32.237 | 3364315 |
| chrA03 | 32.398 | 3436965 |
| chrA03 | 32.772 | 3556473 |
| chrA03 | 33.713 | 3714723 |
| chrA03 | 33.951 | 3714032 |
| chrA03 | 35.863 | 3782263 |
| chrA03 | 35.928 | 4096248 |
| chrA03 | 36.135 | 4023393 |
| chrA03 | 36.207 | 4233535 |
| chrA03 | 36.454 | 4284123 |
| chrA03 | 36.704 | 4110992 |
| chrA03 | 36.794 | 4439338 |
| chrA03 | 37.498 | 4651849 |
| chrA03 | 38.185 | 4985626 |
| chrA03 | 38.363 | 5046935 |
| chrA03 | 38.568 | 5046106 |
| chrA03 | 38.834 | 5068593 |
| chrA03 | 39.197 | 5163471 |
| chrA03 | 39.401 | 5175785 |
| chrA03 | 39.63 | 5236429 |
| chrA03 | 39.799 | 5227278 |
| chrA03 | 39.942 | 5201420 |
| chrA03 | 40.569 | 5431570 |
| chrA03 | 40.773 | 5333727 |
| chrA03 | 41.143 | 5386872 |
| chrA03 | 41.45 | 5510915 |
| chrA03 | 41.619 | 5528075 |
| chrA03 | 42.196 | 5638157 |
| chrA03 | 43.202 | 5777364 |
| chrA03 | 43.273 | 5754786 |
| chrA03 | 43.592 | 5823465 |
| chrA03 | 43.647 | 5875265 |
| chrA03 | 43.787 | 5956038 |
| chrA03 | 43.936 | 5876797 |
| chrA03 | 44.141 | 5993165 |
| chrA03 | 44.338 | 6062836 |
| chrA03 | 44.458 | 6080064 |
| chrA03 | 44.591 | 6296174 |
| chrA03 | 44.899 | 6265627 |
| chrA03 | 44.97 | 6435940 |
| chrA03 | 45.184 | 6457040 |
| chrA03 | 45.717 | 6789347 |
| chrA03 | 46.16 | 6869952 |
| chrA03 | 46.513 | 6757118 |
| chrA03 | 46.569 | 6950898 |
| chrA03 | 46.859 | 6814427 |
| chrA03 | 47.01 | 6932172 |
| chrA03 | 48.8 | 7081534 |
| chrA03 | 48.918 | 7063616 |
| chrA03 | 49.146 | 7033728 |
| chrA03 | 49.146 | 7023737 |
| chrA03 | 49.286 | 7047644 |
| chrA03 | 49.651 | 7098892 |
| chrA03 | 49.874 | 7201742 |
| chrA03 | 50.042 | 7200427 |
| chrA03 | 50.256 | 7273848 |
| chrA03 | 51.137 | 7909858 |
| chrA03 | 51.216 | 7328219 |
| chrA03 | 51.373 | 7664739 |
| chrA03 | 51.493 | 7783612 |
| chrA03 | 51.547 | 7788111 |
| chrA03 | 51.604 | 7444636 |
| chrA03 | 51.714 | 7710265 |
| chrA03 | 51.788 | 7400218 |
| chrA03 | 52.253 | 7692608 |
| chrA03 | 52.54 | 7944869 |
| chrA03 | 53.08 | 7963109 |
| chrA03 | 53.2 | 7962874 |
| chrA03 | 53.501 | 8023164 |
| chrA03 | 53.734 | 7968879 |
| chrA03 | 53.817 | 8024229 |
| chrA03 | 54.357 | 8279723 |
| chrA03 | 54.425 | 8462342 |
| chrA03 | 54.425 | 8467631 |
| chrA03 | 54.483 | 8712736 |
| chrA03 | 54.549 | 8140044 |
| chrA03 | 54.589 | 8425523 |
| chrA03 | 54.637 | 8245343 |
| chrA03 | 55.007 | 8892319 |
| chrA03 | 55.1 | 8903632 |
| chrA03 | 55.189 | 8995659 |
| chrA03 | 55.455 | 9057045 |
| chrA03 | 55.57 | 9085480 |
| chrA03 | 55.709 | 9067153 |
| chrA03 | 55.858 | 9243511 |
| chrA03 | 55.921 | 9197276 |
| chrA03 | 56.003 | 9260462 |
| chrA03 | 56.076 | 9284162 |
| chrA03 | 56.636 | 9330600 |
| chrA03 | 56.844 | 9918396 |
| chrA03 | 56.953 | 9910281 |
| chrA03 | 57.115 | 9973153 |
| chrA03 | 57.227 | 9797270 |
| chrA03 | 58.307 | 10068033 |
| chrA03 | 59.413 | 5681409 |
| chrA03 | 60.874 | 10172331 |
| chrA03 | 60.934 | 10280320 |
| chrA03 | 61.384 | 10434244 |
| chrA03 | 61.523 | 10381422 |
| chrA03 | 61.597 | 10501093 |
| chrA03 | 61.8 | 10809916 |
| chrA03 | 61.955 | 10833570 |
| chrA03 | 62.048 | 10791858 |
| chrA03 | 62.542 | 10925647 |
| chrA03 | 62.542 | 10924453 |
| chrA03 | 62.621 | 10905403 |
| chrA03 | 63.259 | 11149380 |
| chrA03 | 67.015 | 10706010 |
| chrA03 | 68.501 | 11630708 |
| chrA03 | 68.947 | 11765574 |
| chrA03 | 69.065 | 11786486 |
| chrA03 | 69.463 | 12148838 |
| chrA03 | 69.76 | 12210809 |
| chrA03 | 70.07 | 11999944 |
| chrA03 | 70.367 | 12445554 |
| chrA03 | 70.555 | 12371468 |
| chrA03 | 71.117 | 12593641 |
| chrA03 | 71.32 | 12664832 |
| chrA03 | 71.392 | 12722208 |
| chrA03 | 71.473 | 13121599 |
| chrA03 | 71.575 | 13117948 |
| chrA03 | 71.684 | 12656584 |
| chrA03 | 72.119 | 12222493 |
| chrA03 | 75.725 | 13972324 |
| chrA03 | 76.171 | 13995840 |
| chrA03 | 76.476 | 14024243 |
| chrA03 | 76.846 | 14142617 |
| chrA03 | 77.032 | 14131899 |
| chrA03 | 78.763 | 14250131 |
| chrA03 | 78.984 | 14202800 |
| chrA03 | 79.71 | 14325672 |
| chrA03 | 79.814 | 14325231 |
| chrA03 | 85.262 | 15412096 |
| chrA03 | 86.405 | 15565539 |
| chrA03 | 86.71 | 15506516 |
| chrA03 | 86.936 | 15507301 |
| chrA03 | 87.826 | 16074769 |
| chrA03 | 91.19 | 17121343 |
| chrA03 | 91.558 | 17402280 |
| chrA03 | 92.162 | 17782891 |
| chrA03 | 92.805 | 17529460 |
| chrA03 | 93.435 | 17732742 |
| chrA03 | 93.537 | 17662826 |
| chrA03 | 93.756 | 17789122 |
| chrA03 | 93.848 | 17850993 |
| chrA03 | 93.953 | 17840227 |
| chrA03 | 94.26 | 18113031 |
| chrA03 | 94.491 | 18289286 |
| chrA03 | 94.689 | 18139645 |
| chrA03 | 94.946 | 18437304 |
| chrA03 | 95.062 | 18418202 |
| chrA03 | 96.483 | 18806510 |
| chrA03 | 96.9 | 18785931 |
| chrA03 | 97.193 | 18834474 |
| chrA03 | 97.63 | 18836977 |
| chrA03 | 98.083 | 18967249 |
| chrA03 | 98.655 | 18994501 |
| chrA03 | 100.025 | 19014117 |
| chrA03 | 100.103 | 19014982 |
| chrA03 | 100.666 | 19041673 |
| chrA03 | 100.971 | 19083242 |
| chrA03 | 104.747 | 19597727 |
| chrA03 | 107.984 | 19750183 |
| chrA03 | 110.552 | 19974471 |
| chrA03 | 110.841 | 20059138 |
| chrA03 | 110.919 | 20061200 |
| chrA03 | 110.919 | 20061335 |
| chrA03 | 112.981 | 20145216 |
| chrA03 | 113.495 | 20189768 |
| chrA03 | 113.633 | 20182545 |
| chrA03 | 114.049 | 20287178 |
| chrA03 | 114.432 | 20318703 |
| chrA03 | 114.533 | 20289630 |
| chrA03 | 114.836 | 20410825 |
| chrA03 | 115.81 | 20565344 |
| chrA03 | 116.059 | 20618163 |
| chrA03 | 116.321 | 20652978 |
| chrA03 | 116.417 | 20715669 |
| chrA03 | 116.735 | 20796892 |
| chrA03 | 117.141 | 20854640 |
| chrA03 | 117.414 | 20830245 |
| chrA03 | 119.442 | 20922583 |
| chrA03 | 120.15 | 21115417 |
| chrA03 | 120.299 | 21074843 |
| chrA03 | 120.621 | 21115151 |
| chrA03 | 120.806 | 21249589 |
| chrA03 | 121.226 | 21365713 |
| chrA03 | 121.368 | 21411950 |
| chrA03 | 122.212 | 21934769 |
| chrA03 | 122.349 | 21936097 |
| chrA03 | 122.426 | 21937670 |
| chrA03 | 122.508 | 21934872 |
| chrA03 | 122.736 | 21867188 |
| chrA03 | 122.88 | 21837183 |
| chrA03 | 122.88 | 21837152 |
| chrA03 | 123.574 | 22482157 |
| chrA03 | 123.844 | 22063770 |
| chrA03 | 123.955 | 22299684 |
| chrA03 | 124.148 | 22103477 |
| chrA03 | 124.534 | 22635965 |
| chrA03 | 124.966 | 22908975 |
| chrA03 | 125.08 | 22887021 |
| chrA03 | 125.224 | 22879306 |
| chrA03 | 125.572 | 23039245 |
| chrA03 | 125.857 | 23002548 |
| chrA03 | 126.004 | 23157611 |
| chrA03 | 126.2 | 23156585 |
| chrA03 | 126.566 | 23292136 |
| chrA03 | 126.831 | 23359136 |
| chrA03 | 126.98 | 23433030 |
| chrA03 | 127.186 | 23344353 |
| chrA03 | 127.656 | 23509255 |
| chrA03 | 127.89 | 23717660 |
| chrA03 | 128.095 | 23669084 |
| chrA03 | 128.917 | 23771128 |
| chrA03 | 129.138 | 23839829 |
| chrA03 | 129.311 | 23971977 |
| chrA03 | 129.707 | 23973871 |
| chrA03 | 129.892 | 24158852 |
| chrA03 | 130.082 | 23990769 |
| chrA03 | 130.324 | 24602436 |
| chrA03 | 130.324 | 24601695 |
| chrA03 | 130.404 | 24308542 |
| chrA03 | 130.559 | 24311441 |
| chrA03 | 130.741 | 24207765 |
| chrA03 | 131.283 | 24825312 |
| chrA03 | 131.48 | 25125313 |
| chrA03 | 131.884 | 25271708 |
| chrA03 | 133.312 | 25485247 |
| chrA03 | 133.882 | 25684385 |
| chrA03 | 134.08 | 25698786 |
| chrA03 | 134.428 | 25966015 |
| chrA03 | 134.902 | 26674324 |
| chrA03 | 135.39 | 29074115 |
| chrA03 | 135.625 | 27318707 |
| chrA03 | 135.803 | 26949114 |
| chrA03 | 136.148 | 27494411 |
| chrA03 | 136.463 | 27485166 |
| chrA03 | 136.463 | 27480566 |
| chrA03 | 143.757 | 25168917 |
| chrA03 | 144.007 | 25225994 |
| chrA03 | 144.203 | 25128692 |
| chrA03 | 144.358 | 25395292 |
| chrA03 | 145.211 | 25517455 |
| chrA03 | 145.843 | 26174868 |
| chrA03 | 145.973 | 25612851 |
| chrA03 | 146.104 | 25629887 |
| chrA03 | 146.368 | 26157942 |
| chrA03 | 146.613 | 26158164 |
| chrA03 | 147.06 | 26641833 |
| chrA03 | 147.838 | 28972450 |
| chrA03 | 152.407 | 27022421 |
| chrA03 | 168.869 | 25395141 |
| chrA04 | 4.427 | 274063 |
| chrA04 | 4.726 | 528069 |
| chrA04 | 5.496 | 656241 |
| chrA04 | 7.141 | 1216574 |
| chrA04 | 7.283 | 1089052 |
| chrA04 | 7.522 | 808626 |
| chrA04 | 7.654 | 812101 |
| chrA04 | 7.875 | 911527 |
| chrA04 | 7.949 | 1117692 |
| chrA04 | 8.117 | 839876 |
| chrA04 | 8.242 | 1104975 |
| chrA04 | 8.339 | 1221770 |
| chrA04 | 9.359 | 1342593 |
| chrA04 | 11.078 | 8805725 |
| chrA04 | 11.578 | 1519942 |
| chrA04 | 11.872 | 1569824 |
| chrA04 | 12.248 | 2579766 |
| chrA04 | 12.401 | 4871884 |
| chrA04 | 12.487 | 2375666 |
| chrA04 | 12.583 | 2436470 |
| chrA04 | 12.666 | 3756610 |
| chrA04 | 12.87 | 3754552 |
| chrA04 | 12.995 | 7406914 |
| chrA04 | 13.027 | 8817019 |
| chrA04 | 13.286 | 2614752 |
| chrA04 | 13.405 | 1562018 |
| chrA04 | 13.514 | 4219158 |
| chrA04 | 13.56 | 4529669 |
| chrA04 | 13.626 | 8959743 |
| chrA04 | 13.775 | 7190885 |
| chrA04 | 14.036 | 7600461 |
| chrA04 | 14.153 | 8477896 |
| chrA04 | 14.811 | 10076029 |
| chrA04 | 15.043 | 10189137 |
| chrA04 | 15.126 | 10207742 |
| chrA04 | 15.396 | 10415881 |
| chrA04 | 15.63 | 10503359 |
| chrA04 | 15.845 | 11248077 |
| chrA04 | 15.953 | 11276700 |
| chrA04 | 16.158 | 11301538 |
| chrA04 | 17.561 | 11594716 |
| chrA04 | 17.793 | 11806909 |
| chrA04 | 17.987 | 11747667 |
| chrA04 | 18.236 | 12028094 |
| chrA04 | 18.439 | 12041741 |
| chrA04 | 18.965 | 12172533 |
| chrA04 | 21.055 | 12518886 |
| chrA04 | 21.331 | 12744228 |
| chrA04 | 21.423 | 12822950 |
| chrA04 | 21.568 | 12699255 |
| chrA04 | 21.641 | 12746579 |
| chrA04 | 21.706 | 12876532 |
| chrA04 | 21.848 | 12886079 |
| chrA04 | 22.626 | 12971640 |
| chrA04 | 23.977 | 13015284 |
| chrA04 | 27.262 | 13224235 |
| chrA04 | 29.101 | 13252757 |
| chrA04 | 30.278 | 13395962 |
| chrA04 | 30.544 | 13402608 |
| chrA04 | 31.804 | 13577856 |
| chrA04 | 32.106 | 13629268 |
| chrA04 | 32.341 | 13584211 |
| chrA04 | 33.239 | 13747877 |
| chrA04 | 34.915 | 13850762 |
| chrA04 | 35.083 | 13826022 |
| chrA04 | 35.525 | 13865743 |
| chrA04 | 35.64 | 13996640 |
| chrA04 | 41.192 | 14690651 |
| chrA04 | 41.483 | 14767384 |
| chrA04 | 42.43 | 14930797 |
| chrA04 | 42.602 | 14880989 |
| chrA04 | 42.943 | 15003568 |
| chrA04 | 43.113 | 15141614 |
| chrA04 | 43.342 | 15190572 |
| chrA04 | 43.756 | 15333153 |
| chrA04 | 43.968 | 15347561 |
| chrA04 | 44.573 | 15393188 |
| chrA04 | 45.134 | 15437068 |
| chrA04 | 45.216 | 15442086 |
| chrA04 | 45.332 | 15418078 |
| chrA04 | 45.515 | 15484476 |
| chrA04 | 45.679 | 15467495 |
| chrA04 | 52.476 | 16368264 |
| chrA04 | 52.577 | 16314606 |
| chrA04 | 52.898 | 16333507 |
| chrA04 | 53.768 | 16484358 |
| chrA04 | 53.923 | 16581892 |
| chrA04 | 54.542 | 16513378 |
| chrA04 | 54.727 | 16620493 |
| chrA04 | 54.853 | 16630467 |
| chrA04 | 54.988 | 16618742 |
| chrA04 | 55.106 | 16652972 |
| chrA04 | 55.179 | 16733484 |
| chrA04 | 55.466 | 16649387 |
| chrA04 | 55.9 | 17100321 |
| chrA04 | 56.105 | 17214955 |
| chrA04 | 56.287 | 17182533 |
| chrA04 | 56.411 | 17002811 |
| chrA04 | 56.544 | 17364075 |
| chrA04 | 56.912 | 17024731 |
| chrA04 | 57.052 | 17148241 |
| chrA04 | 57.244 | 17441108 |
| chrA04 | 57.443 | 17388362 |
| chrA04 | 58.561 | 17577005 |
| chrA04 | 58.777 | 17689640 |
| chrA04 | 59.241 | 17513781 |
| chrA04 | 59.501 | 17648615 |
| chrA04 | 60.036 | 17733461 |
| chrA04 | 60.804 | 17847969 |
| chrA04 | 60.933 | 18049039 |
| chrA04 | 61.279 | 17870549 |
| chrA04 | 62.127 | 18447722 |
| chrA04 | 62.431 | 18177426 |
| chrA04 | 62.642 | 18444919 |
| chrA04 | 63.218 | 18176783 |
| chrA04 | 63.417 | 18174269 |
| chrA04 | 64.774 | 18967949 |
| chrA04 | 65.114 | 19050710 |
| chrA05 | 11.383 | 775961 |
| chrA05 | 12.232 | 78960 |
| chrA05 | 12.354 | 91342 |
| chrA05 | 12.695 | 216922 |
| chrA05 | 13.214 | 704986 |
| chrA05 | 13.493 | 794360 |
| chrA05 | 13.667 | 509276 |
| chrA05 | 14.237 | 809294 |
| chrA05 | 14.659 | 867923 |
| chrA05 | 19.477 | 1058664 |
| chrA05 | 19.575 | 1158576 |
| chrA05 | 19.752 | 980212 |
| chrA05 | 20.016 | 1234204 |
| chrA05 | 20.174 | 1102186 |
| chrA05 | 20.643 | 1271893 |
| chrA05 | 20.975 | 1397345 |
| chrA05 | 21.108 | 1370395 |
| chrA05 | 21.336 | 1339238 |
| chrA05 | 21.819 | 1414174 |
| chrA05 | 22.201 | 1426744 |
| chrA05 | 22.778 | 1464917 |
| chrA05 | 23.135 | 1467456 |
| chrA05 | 23.37 | 1615817 |
| chrA05 | 23.37 | 1615841 |
| chrA05 | 23.575 | 1619642 |
| chrA05 | 24.424 | 1815056 |
| chrA05 | 24.731 | 1834157 |
| chrA05 | 25.224 | 1894996 |
| chrA05 | 25.418 | 1887184 |
| chrA05 | 25.681 | 1823595 |
| chrA05 | 29 | 2006022 |
| chrA05 | 29.733 | 2024653 |
| chrA05 | 30.501 | 2199230 |
| chrA05 | 33.095 | 2385384 |
| chrA05 | 33.259 | 2396176 |
| chrA05 | 33.408 | 2474838 |
| chrA05 | 33.777 | 2473317 |
| chrA05 | 33.889 | 2523735 |
| chrA05 | 36.596 | 2720813 |
| chrA05 | 36.687 | 2702444 |
| chrA05 | 36.974 | 2757079 |
| chrA05 | 37.017 | 2710787 |
| chrA05 | 37.64 | 2888867 |
| chrA05 | 38.023 | 2798858 |
| chrA05 | 38.094 | 2749236 |
| chrA05 | 40.04 | 2981875 |
| chrA05 | 40.64 | 3109985 |
| chrA05 | 40.974 | 3207064 |
| chrA05 | 41.751 | 3275213 |
| chrA05 | 42.096 | 3334697 |
| chrA05 | 42.303 | 3292081 |
| chrA05 | 44.181 | 3475734 |
| chrA05 | 44.433 | 3606756 |
| chrA05 | 44.567 | 3550572 |
| chrA05 | 45.26 | 3670642 |
| chrA05 | 45.572 | 3709392 |
| chrA05 | 46.439 | 3821353 |
| chrA05 | 47.029 | 3887723 |
| chrA05 | 47.196 | 3910981 |
| chrA05 | 47.305 | 3854515 |
| chrA05 | 47.476 | 3987318 |
| chrA05 | 47.588 | 3934184 |
| chrA05 | 47.65 | 3959877 |
| chrA05 | 47.83 | 4079406 |
| chrA05 | 47.988 | 4106650 |
| chrA05 | 48.358 | 4387403 |
| chrA05 | 48.55 | 4287564 |
| chrA05 | 48.679 | 4478483 |
| chrA05 | 49.007 | 4494832 |
| chrA05 | 50.168 | 4796601 |
| chrA05 | 51.484 | 4815695 |
| chrA05 | 51.595 | 4820543 |
| chrA05 | 52.621 | 4920136 |
| chrA05 | 52.954 | 5067801 |
| chrA05 | 53.348 | 5103117 |
| chrA05 | 53.653 | 5118504 |
| chrA05 | 53.785 | 5036053 |
| chrA05 | 54.297 | 5183107 |
| chrA05 | 55.064 | 5223317 |
| chrA05 | 55.557 | 5254405 |
| chrA05 | 56.203 | 5424733 |
| chrA05 | 56.634 | 5418518 |
| chrA05 | 56.864 | 5460601 |
| chrA05 | 58.001 | 5766923 |
| chrA05 | 58.381 | 5848126 |
| chrA05 | 59.917 | 6316666 |
| chrA05 | 61.259 | 7095735 |
| chrA05 | 63.502 | 15268294 |
| chrA05 | 63.664 | 15258685 |
| chrA05 | 63.723 | 15258102 |
| chrA05 | 63.876 | 15433740 |
| chrA05 | 63.948 | 15587612 |
| chrA05 | 64.063 | 14469455 |
| chrA05 | 64.214 | 15524071 |
| chrA05 | 64.313 | 15422387 |
| chrA05 | 64.431 | 14207064 |
| chrA05 | 64.602 | 14220566 |
| chrA05 | 64.882 | 16396737 |
| chrA05 | 65.041 | 16444626 |
| chrA05 | 65.15 | 16682917 |
| chrA05 | 65.312 | 16423724 |
| chrA05 | 65.626 | 16425904 |
| chrA05 | 65.795 | 16397444 |
| chrA05 | 65.912 | 16400078 |
| chrA05 | 71.082 | 17323699 |
| chrA05 | 71.52 | 17484140 |
| chrA05 | 72.152 | 17453689 |
| chrA05 | 73.333 | 17729649 |
| chrA05 | 74.194 | 17889839 |
| chrA06 | 5.882 | 808369 |
| chrA06 | 6.535 | 293214 |
| chrA06 | 6.63 | 834670 |
| chrA06 | 7.171 | 860305 |
| chrA06 | 7.388 | 648918 |
| chrA06 | 7.933 | 2243102 |
| chrA06 | 8.117 | 1480632 |
| chrA06 | 8.209 | 1663635 |
| chrA06 | 8.484 | 1398435 |
| chrA06 | 8.653 | 1350542 |
| chrA06 | 8.796 | 1447111 |
| chrA06 | 8.902 | 1475576 |
| chrA06 | 9.256 | 1780676 |
| chrA06 | 9.432 | 1749778 |
| chrA06 | 10.548 | 1837831 |
| chrA06 | 10.774 | 2256266 |
| chrA06 | 11.801 | 2299475 |
| chrA06 | 12.607 | 2420057 |
| chrA06 | 12.95 | 2519419 |
| chrA06 | 13.09 | 2501065 |
| chrA06 | 15.132 | 2594590 |
| chrA06 | 16.083 | 2609985 |
| chrA06 | 16.431 | 2781204 |
| chrA06 | 16.675 | 2772100 |
| chrA06 | 17.829 | 2832051 |
| chrA06 | 18.094 | 2838509 |
| chrA06 | 18.267 | 2876169 |
| chrA06 | 18.478 | 2900839 |
| chrA06 | 18.593 | 2881641 |
| chrA06 | 19.995 | 2910606 |
| chrA06 | 20.563 | 2980527 |
| chrA06 | 20.944 | 2987271 |
| chrA06 | 22.539 | 3059985 |
| chrA06 | 24.156 | 3156855 |
| chrA06 | 25.512 | 3171120 |
| chrA06 | 26.311 | 3198486 |
| chrA06 | 26.606 | 3216421 |
| chrA06 | 27.357 | 3255822 |
| chrA06 | 29.79 | 3450047 |
| chrA06 | 29.79 | 3424224 |
| chrA06 | 30.18 | 3403373 |
| chrA06 | 30.353 | 3456341 |
| chrA06 | 31.352 | 3504688 |
| chrA06 | 32.343 | 3569753 |
| chrA06 | 32.472 | 3584768 |
| chrA06 | 33.471 | 3719822 |
| chrA06 | 33.606 | 3759077 |
| chrA06 | 34.176 | 3775034 |
| chrA06 | 36.778 | 3790094 |
| chrA06 | 37.154 | 3816738 |
| chrA06 | 37.401 | 3904587 |
| chrA06 | 37.743 | 21200155 |
| chrA06 | 38.067 | 4265995 |
| chrA06 | 38.574 | 4144365 |
| chrA06 | 38.79 | 4351499 |
| chrA06 | 39.624 | 4414959 |
| chrA06 | 41.697 | 4647011 |
| chrA06 | 41.887 | 4690694 |
| chrA06 | 42.085 | 4748748 |
| chrA06 | 42.49 | 4755715 |
| chrA06 | 42.931 | 4899608 |
| chrA06 | 43.248 | 4851811 |
| chrA06 | 44.24 | 5044632 |
| chrA06 | 46.021 | 5119767 |
| chrA06 | 46.903 | 5254348 |
| chrA06 | 48.305 | 10493839 |
| chrA06 | 48.895 | 5334855 |
| chrA06 | 49.94 | 15357809 |
| chrA06 | 54.746 | 5878786 |
| chrA06 | 54.919 | 5916929 |
| chrA06 | 55.073 | 5889729 |
| chrA06 | 55.862 | 6111683 |
| chrA06 | 56.244 | 6131678 |
| chrA06 | 57.154 | 6455868 |
| chrA06 | 57.796 | 6428753 |
| chrA06 | 57.899 | 6474939 |
| chrA06 | 58.207 | 6374046 |
| chrA06 | 58.571 | 6807482 |
| chrA06 | 59.018 | 9433331 |
| chrA06 | 59.361 | 9450120 |
| chrA06 | 59.599 | 7023943 |
| chrA06 | 59.815 | 9449623 |
| chrA06 | 59.98 | 7398951 |
| chrA06 | 60.05 | 9448176 |
| chrA06 | 60.075 | 9450440 |
| chrA06 | 60.201 | 8477242 |
| chrA06 | 60.201 | 8471917 |
| chrA06 | 60.251 | 9448389 |
| chrA06 | 60.393 | 8465944 |
| chrA06 | 60.551 | 7658193 |
| chrA06 | 60.882 | 10492114 |
| chrA06 | 60.916 | 15547416 |
| chrA06 | 60.993 | 8388687 |
| chrA06 | 61.561 | 16524095 |
| chrA06 | 62.272 | 16890416 |
| chrA06 | 62.461 | 17284641 |
| chrA06 | 62.741 | 17285232 |
| chrA06 | 63.314 | 17351635 |
| chrA06 | 63.516 | 17353339 |
| chrA06 | 63.667 | 17449152 |
| chrA06 | 64.118 | 18123498 |
| chrA06 | 64.322 | 18115180 |
| chrA06 | 68.329 | 18424846 |
| chrA06 | 68.845 | 18438902 |
| chrA06 | 69.23 | 18609964 |
| chrA06 | 69.404 | 18636473 |
| chrA06 | 69.653 | 18500340 |
| chrA06 | 70.36 | 18727699 |
| chrA06 | 71.199 | 18590256 |
| chrA06 | 72.237 | 19172653 |
| chrA06 | 72.491 | 19437797 |
| chrA06 | 73.409 | 19504401 |
| chrA06 | 73.562 | 19517797 |
| chrA06 | 73.981 | 19537304 |
| chrA06 | 74.241 | 19624985 |
| chrA06 | 75.276 | 19763994 |
| chrA06 | 75.626 | 19800472 |
| chrA06 | 75.818 | 19802477 |
| chrA06 | 76.692 | 19998643 |
| chrA06 | 76.84 | 20045767 |
| chrA06 | 77.209 | 20089160 |
| chrA06 | 77.342 | 20111244 |
| chrA06 | 78.156 | 20372627 |
| chrA06 | 78.607 | 20421993 |
| chrA06 | 79.174 | 20526414 |
| chrA06 | 79.333 | 20526710 |
| chrA06 | 80.26 | 20588092 |
| chrA06 | 80.597 | 20587693 |
| chrA06 | 80.787 | 20609632 |
| chrA06 | 80.851 | 20679725 |
| chrA06 | 82.8 | 20953562 |
| chrA06 | 83.037 | 20843289 |
| chrA06 | 83.146 | 20842824 |
| chrA06 | 83.549 | 20986276 |
| chrA06 | 84.282 | 21140367 |
| chrA06 | 85.507 | 21268671 |
| chrA06 | 86.131 | 21416129 |
| chrA06 | 86.288 | 21577548 |
| chrA06 | 86.57 | 21646945 |
| chrA06 | 89.47 | 21689121 |
| chrA06 | 90.051 | 21807229 |
| chrA06 | 90.255 | 21823420 |
| chrA06 | 90.334 | 21943630 |
| chrA06 | 90.418 | 21872073 |
| chrA06 | 90.703 | 21809560 |
| chrA06 | 91.084 | 22092576 |
| chrA06 | 91.899 | 22298095 |
| chrA06 | 92.154 | 22307079 |
| chrA06 | 92.531 | 22315285 |
| chrA06 | 92.531 | 22315191 |
| chrA06 | 93.225 | 22353580 |
| chrA06 | 93.414 | 22482161 |
| chrA06 | 94.024 | 22658704 |
| chrA06 | 94.105 | 22602452 |
| chrA06 | 95.143 | 22750602 |
| chrA06 | 95.615 | 22760599 |
| chrA06 | 97.718 | 22863731 |
| chrA06 | 97.846 | 22930229 |
| chrA06 | 98.097 | 23041686 |
| chrA06 | 98.367 | 23138149 |
| chrA06 | 98.596 | 22839501 |
| chrA06 | 98.772 | 23155675 |
| chrA06 | 100.729 | 23238523 |
| chrA06 | 101.446 | 23275614 |
| chrA06 | 103.841 | 23320848 |
| chrA06 | 104.015 | 23338465 |
| chrA06 | 104.479 | 23371762 |
| chrA06 | 106.28 | 23695767 |
| chrA06 | 106.453 | 23605110 |
| chrA06 | 106.678 | 23633611 |
| chrA06 | 110.33 | 22493242 |
| chrA06 | 127.845 | 24317023 |
| chrA07 | 3.348 | 7751992 |
| chrA07 | 17.299 | 7782028 |
| chrA07 | 18.413 | 592104 |
| chrA07 | 18.533 | 598809 |
| chrA07 | 18.655 | 1127418 |
| chrA07 | 18.796 | 623625 |
| chrA07 | 19.137 | 887541 |
| chrA07 | 19.277 | 1110100 |
| chrA07 | 20.527 | 1324452 |
| chrA07 | 20.666 | 1323848 |
| chrA07 | 20.854 | 860202 |
| chrA07 | 21.787 | 1897356 |
| chrA07 | 22.513 | 2643958 |
| chrA07 | 22.597 | 1787583 |
| chrA07 | 22.787 | 4669685 |
| chrA07 | 22.864 | 5401304 |
| chrA07 | 23.01 | 3125823 |
| chrA07 | 23.112 | 5034512 |
| chrA07 | 23.112 | 1769019 |
| chrA07 | 23.158 | 4180217 |
| chrA07 | 23.158 | 9913342 |
| chrA07 | 23.216 | 4264686 |
| chrA07 | 23.33 | 6093157 |
| chrA07 | 23.468 | 5864772 |
| chrA07 | 23.593 | 5754261 |
| chrA07 | 24.735 | 7152400 |
| chrA07 | 24.844 | 7018335 |
| chrA07 | 25.001 | 7588963 |
| chrA07 | 25.153 | 7018634 |
| chrA07 | 25.281 | 7297422 |
| chrA07 | 25.397 | 7670437 |
| chrA07 | 25.66 | 7233191 |
| chrA07 | 26.171 | 7918173 |
| chrA07 | 26.171 | 7904548 |
| chrA07 | 26.594 | 7918050 |
| chrA07 | 26.594 | 7904425 |
| chrA07 | 27.128 | 8102004 |
| chrA07 | 27.256 | 8125661 |
| chrA07 | 27.477 | 8169480 |
| chrA07 | 27.551 | 8173898 |
| chrA07 | 27.622 | 8137352 |
| chrA07 | 27.69 | 8132960 |
| chrA07 | 28.043 | 8305796 |
| chrA07 | 28.454 | 8137216 |
| chrA07 | 28.821 | 8699986 |
| chrA07 | 28.878 | 9667219 |
| chrA07 | 31.946 | 9190861 |
| chrA07 | 33.119 | 9505899 |
| chrA07 | 33.255 | 9583415 |
| chrA07 | 33.255 | 22606853 |
| chrA07 | 34.956 | 9698330 |
| chrA07 | 35.279 | 9888458 |
| chrA07 | 35.534 | 9795531 |
| chrA07 | 36.153 | 10202033 |
| chrA07 | 38.253 | 10443749 |
| chrA07 | 39.124 | 10550952 |
| chrA07 | 39.222 | 10557440 |
| chrA07 | 39.348 | 10551899 |
| chrA07 | 39.615 | 10559313 |
| chrA07 | 41.873 | 10849261 |
| chrA07 | 46.076 | 11076535 |
| chrA07 | 46.289 | 11117114 |
| chrA07 | 47.135 | 11131531 |
| chrA07 | 48 | 11221881 |
| chrA07 | 50.021 | 11280234 |
| chrA07 | 50.953 | 11369948 |
| chrA07 | 51.469 | 11321643 |
| chrA07 | 51.664 | 11360785 |
| chrA07 | 52.249 | 11374841 |
| chrA07 | 52.916 | 11651366 |
| chrA07 | 53.115 | 11569343 |
| chrA07 | 53.175 | 11563604 |
| chrA07 | 53.24 | 11667701 |
| chrA07 | 53.452 | 11506300 |
| chrA07 | 53.694 | 11559931 |
| chrA07 | 54.306 | 11748598 |
| chrA07 | 54.481 | 11734445 |
| chrA07 | 55.982 | 11816993 |
| chrA07 | 56.179 | 11833047 |
| chrA07 | 56.34 | 11889819 |
| chrA07 | 56.592 | 11888810 |
| chrA07 | 56.884 | 11935160 |
| chrA07 | 57.816 | 12190825 |
| chrA07 | 58.091 | 12111092 |
| chrA07 | 58.471 | 12175647 |
| chrA07 | 59.338 | 12252071 |
| chrA07 | 59.56 | 12686360 |
| chrA07 | 59.637 | 12540405 |
| chrA07 | 59.715 | 12334025 |
| chrA07 | 59.882 | 12629830 |
| chrA07 | 60.258 | 12813027 |
| chrA07 | 60.327 | 12812021 |
| chrA07 | 60.427 | 12809010 |
| chrA07 | 60.511 | 12924384 |
| chrA07 | 61.076 | 12831508 |
| chrA07 | 61.213 | 12574859 |
| chrA07 | 61.376 | 12907664 |
| chrA07 | 61.653 | 12951421 |
| chrA07 | 63.831 | 13179963 |
| chrA07 | 64.707 | 13399907 |
| chrA07 | 64.707 | 13399379 |
| chrA07 | 64.934 | 13434480 |
| chrA07 | 65.158 | 13428594 |
| chrA07 | 66.56 | 13518578 |
| chrA07 | 67.302 | 13652618 |
| chrA07 | 67.381 | 13694510 |
| chrA07 | 70.21 | 14028305 |
| chrA07 | 70.91 | 14444638 |
| chrA07 | 71.211 | 14216411 |
| chrA07 | 71.402 | 14170130 |
| chrA07 | 71.681 | 14483796 |
| chrA07 | 72.129 | 14656494 |
| chrA07 | 72.531 | 14621648 |
| chrA07 | 72.586 | 14621208 |
| chrA07 | 73.16 | 14739440 |
| chrA07 | 73.228 | 14713265 |
| chrA07 | 73.518 | 14837318 |
| chrA07 | 73.657 | 14816904 |
| chrA07 | 73.89 | 14799465 |
| chrA07 | 74.599 | 14937647 |
| chrA07 | 74.771 | 14938303 |
| chrA07 | 74.904 | 14876938 |
| chrA07 | 75.247 | 14971021 |
| chrA07 | 75.247 | 14967589 |
| chrA07 | 76.112 | 15081894 |
| chrA07 | 76.272 | 15114434 |
| chrA07 | 76.469 | 15100064 |
| chrA07 | 76.95 | 15218700 |
| chrA07 | 77.06 | 15209227 |
| chrA07 | 77.294 | 15205857 |
| chrA07 | 77.409 | 15251749 |
| chrA07 | 77.831 | 15272253 |
| chrA07 | 78.048 | 15270717 |
| chrA07 | 78.25 | 15272140 |
| chrA07 | 81.261 | 15397996 |
| chrA07 | 82.133 | 15490115 |
| chrA07 | 82.689 | 15493102 |
| chrA07 | 83.51 | 15551085 |
| chrA07 | 96.674 | 17272587 |
| chrA07 | 96.797 | 17238421 |
| chrA07 | 97.068 | 17699661 |
| chrA07 | 97.262 | 17078568 |
| chrA07 | 97.52 | 17673830 |
| chrA07 | 98.028 | 17734639 |
| chrA07 | 98.73 | 17881059 |
| chrA07 | 98.73 | 20717071 |
| chrA07 | 99.391 | 18082919 |
| chrA07 | 101.602 | 18835488 |
| chrA07 | 104.888 | 20769186 |
| chrA07 | 104.888 | 17813040 |
| chrA07 | 105.348 | 20803071 |
| chrA07 | 105.348 | 20798584 |
| chrA07 | 108.032 | 22239663 |
| chrA07 | 114.267 | 18054172 |
| chrA07 | 115.363 | 18089869 |
| chrA07 | 115.363 | 20352713 |
| chrA07 | 116.709 | 18869779 |
| chrA07 | 117.599 | 19299435 |
| chrA07 | 118.515 | 19667944 |
| chrA07 | 118.757 | 19857512 |
| chrA07 | 124.062 | 19941001 |
| chrA08 | 0 | 2012815 |
| chrA08 | 16.196 | 2022082 |
| chrA08 | 17.59 | 1852742 |
| chrA08 | 17.81 | 2088677 |
| chrA08 | 17.865 | 2344487 |
| chrA08 | 18.008 | 2221350 |
| chrA08 | 18.044 | 2188898 |
| chrA08 | 18.21 | 2685047 |
| chrA08 | 19.612 | 8367741 |
| chrA08 | 19.83 | 8710704 |
| chrA08 | 22.574 | 9165729 |
| chrA08 | 22.737 | 9198696 |
| chrA08 | 22.831 | 9168990 |
| chrA08 | 22.97 | 9295219 |
| chrA08 | 24.919 | 10374004 |
| chrA08 | 28.244 | 10839241 |
| chrA08 | 29.027 | 10967594 |
| chrA08 | 29.265 | 11040847 |
| chrA08 | 30.675 | 11061397 |
| chrA08 | 30.712 | 11075459 |
| chrA08 | 31.061 | 11285266 |
| chrA08 | 31.368 | 11632645 |
| chrA08 | 31.396 | 11367676 |
| chrA08 | 31.598 | 11580354 |
| chrA08 | 31.744 | 11767526 |
| chrA08 | 32.014 | 11696209 |
| chrA08 | 32.314 | 11862519 |
| chrA08 | 32.412 | 11858854 |
| chrA08 | 32.559 | 11905699 |
| chrA08 | 32.736 | 11962254 |
| chrA08 | 32.902 | 12216058 |
| chrA08 | 33.525 | 12351977 |
| chrA08 | 34.779 | 12564306 |
| chrA08 | 36.131 | 12815846 |
| chrA08 | 36.492 | 12830803 |
| chrA08 | 36.972 | 13125246 |
| chrA08 | 37.088 | 13151813 |
| chrA08 | 37.429 | 13419313 |
| chrA08 | 38.028 | 13461041 |
| chrA08 | 38.226 | 13488709 |
| chrA08 | 39.053 | 13511158 |
| chrA08 | 39.813 | 13598254 |
| chrA08 | 40.976 | 13632443 |
| chrA08 | 41.412 | 13651297 |
| chrA08 | 42.178 | 13673345 |
| chrA08 | 44.013 | 13762189 |
| chrA08 | 44.056 | 13741451 |
| chrA08 | 44.435 | 13861297 |
| chrA08 | 44.618 | 13812934 |
| chrA08 | 44.857 | 13807371 |
| chrA08 | 53.602 | 13492746 |
| chrA08 | 70.256 | 18358428 |
| chrA08 | 70.394 | 18320267 |
| chrA08 | 70.74 | 18442028 |
| chrA08 | 78.467 | 17422529 |
| chrA08 | 80.25 | 17667561 |
| chrA08 | 80.438 | 17699454 |
| chrA08 | 80.65 | 17777666 |
| chrA08 | 80.718 | 17871485 |
| chrA08 | 81.071 | 17788598 |
| chrA08 | 82.971 | 18008696 |
| chrA08 | 96.191 | 17707014 |
| chrA08 | 96.392 | 17638284 |
| chrA08 | 96.766 | 17788479 |
| chrA08 | 98.881 | 17957808 |
| chrA09 | 0 | 2461046 |
| chrA09 | 12.186 | 121666 |
| chrA09 | 13.72 | 371013 |
| chrA09 | 14.376 | 733179 |
| chrA09 | 25.853 | 686922 |
| chrA09 | 26.013 | 686146 |
| chrA09 | 26.442 | 519474 |
| chrA09 | 32.476 | 1999269 |
| chrA09 | 33.787 | 2208430 |
| chrA09 | 37.102 | 2872690 |
| chrA09 | 39.394 | 3264059 |
| chrA09 | 41.357 | 3834966 |
| chrA09 | 45.493 | 4344816 |
| chrA09 | 45.757 | 3999641 |
| chrA09 | 49.268 | 4756463 |
| chrA09 | 49.853 | 4757761 |
| chrA09 | 50.849 | 4897431 |
| chrA09 | 51.729 | 5037575 |
| chrA09 | 51.926 | 10215109 |
| chrA09 | 51.926 | 5141648 |
| chrA09 | 52.088 | 5484403 |
| chrA09 | 52.159 | 5238616 |
| chrA09 | 52.487 | 5546078 |
| chrA09 | 52.731 | 5553856 |
| chrA09 | 52.803 | 5655934 |
| chrA09 | 53.475 | 5789453 |
| chrA09 | 53.598 | 6402433 |
| chrA09 | 53.812 | 5883474 |
| chrA09 | 53.894 | 6357973 |
| chrA09 | 53.983 | 6354477 |
| chrA09 | 54.046 | 6369513 |
| chrA09 | 54.25 | 5878083 |
| chrA09 | 56.109 | 8020232 |
| chrA09 | 56.99 | 8280227 |
| chrA09 | 61.045 | 9293844 |
| chrA09 | 61.595 | 9519356 |
| chrA09 | 63.463 | 9616807 |
| chrA09 | 67.777 | 10434935 |
| chrA09 | 68.515 | 10920016 |
| chrA09 | 69.984 | 11160035 |
| chrA09 | 72.602 | 13428172 |
| chrA09 | 72.602 | 13422147 |
| chrA09 | 72.602 | 13420871 |
| chrA09 | 72.771 | 13350352 |
| chrA09 | 73.345 | 13578472 |
| chrA09 | 73.765 | 13622857 |
| chrA09 | 75.469 | 13953313 |
| chrA09 | 76.28 | 14241922 |
| chrA09 | 76.537 | 16914545 |
| chrA09 | 76.845 | 16068176 |
| chrA09 | 77.584 | 17627884 |
| chrA09 | 93.248 | 23469100 |
| chrA09 | 93.328 | 23363482 |
| chrA09 | 94.89 | 23731316 |
| chrA09 | 95.866 | 23840500 |
| chrA09 | 96.096 | 23840508 |
| chrA09 | 96.563 | 24009563 |
| chrA09 | 96.771 | 24040711 |
| chrA09 | 99.084 | 24151621 |
| chrA09 | 99.195 | 24130704 |
| chrA09 | 99.309 | 24138701 |
| chrA09 | 99.578 | 23084590 |
| chrA09 | 99.73 | 24197901 |
| chrA09 | 100.988 | 24429999 |
| chrA09 | 101.392 | 24513228 |
| chrA09 | 101.392 | 24519807 |
| chrA09 | 101.565 | 24473358 |
| chrA09 | 103.219 | 24621813 |
| chrA09 | 103.454 | 24629575 |
| chrA09 | 105.442 | 24788218 |
| chrA09 | 105.963 | 24933648 |
| chrA09 | 114.686 | 25526901 |
| chrA09 | 114.686 | 25525493 |
| chrA09 | 115.748 | 25575773 |
| chrA09 | 116.001 | 25977255 |
| chrA09 | 116.266 | 26005785 |
| chrA09 | 117.418 | 26047535 |
| chrA09 | 117.817 | 26063652 |
| chrA09 | 118.286 | 26082531 |
| chrA09 | 118.886 | 26154398 |
| chrA09 | 119.533 | 26463759 |
| chrA09 | 120.461 | 26506646 |
| chrA09 | 120.58 | 26480632 |
| chrA09 | 121.546 | 26642683 |
| chrA09 | 121.863 | 22514989 |
| chrA09 | 122.313 | 26730004 |
| chrA09 | 122.934 | 26813894 |
| chrA09 | 124.136 | 27063786 |
| chrA09 | 124.787 | 27171689 |
| chrA09 | 125.282 | 27210242 |
| chrA09 | 126.13 | 27243273 |
| chrA09 | 126.9 | 27281999 |
| chrA09 | 127.803 | 27314004 |
| chrA09 | 128.448 | 27828014 |
| chrA09 | 129.112 | 27916750 |
| chrA09 | 129.112 | 27913125 |
| chrA09 | 131.282 | 28002453 |
| chrA09 | 131.443 | 28061065 |
| chrA09 | 131.637 | 28062787 |
| chrA09 | 131.985 | 28180373 |
| chrA09 | 132.414 | 28259457 |
| chrA09 | 132.566 | 28236550 |
| chrA09 | 132.805 | 30897285 |
| chrA09 | 132.969 | 28297988 |
| chrA09 | 133.133 | 28310895 |
| chrA09 | 135.46 | 28494060 |
| chrA09 | 135.607 | 28582762 |
| chrA09 | 135.81 | 28579013 |
| chrA09 | 136.246 | 28622919 |
| chrA09 | 136.824 | 28653978 |
| chrA09 | 136.95 | 28683934 |
| chrA09 | 136.95 | 28683332 |
| chrA09 | 136.95 | 28685707 |
| chrA09 | 136.95 | 28685116 |
| chrA09 | 136.95 | 28684525 |
| chrA09 | 137.444 | 28709327 |
| chrA09 | 137.852 | 29244147 |
| chrA09 | 137.955 | 29184374 |
| chrA09 | 138.534 | 29417644 |
| chrA09 | 139.015 | 29661244 |
| chrA09 | 139.583 | 29715163 |
| chrA09 | 139.782 | 29786870 |
| chrA09 | 141.25 | 29887581 |
| chrA09 | 141.883 | 29995672 |
| chrA09 | 142.069 | 29995412 |
| chrA09 | 142.301 | 30032242 |
| chrA09 | 143.3 | 30521790 |
| chrA09 | 143.403 | 30146254 |
| chrA09 | 143.546 | 30451376 |
| chrA09 | 145.08 | 30615168 |
| chrA09 | 146.185 | 30697704 |
| chrA09 | 146.441 | 30688730 |
| chrA09 | 146.672 | 30909757 |
| chrA09 | 147.107 | 30730863 |
| chrA09 | 147.515 | 30824839 |
| chrA09 | 149.117 | 31060810 |
| chrA09 | 149.462 | 31093736 |
| chrA09 | 149.772 | 31297291 |
| chrA09 | 149.881 | 31193121 |
| chrA09 | 150.323 | 31671459 |
| chrA09 | 154.015 | 32408505 |
| chrA09 | 155.108 | 32536990 |
| chrA09 | 155.328 | 32761880 |
| chrA09 | 155.47 | 32793459 |
| chrA09 | 156.129 | 32829675 |
| chrA09 | 160.215 | 33753723 |
| chrA09 | 160.304 | 33833004 |
| chrA09 | 160.855 | 33757815 |
| chrA09 | 161.12 | 33839298 |
| chrA09 | 165.7 | 31707866 |
| chrC01 | 10.962 | 136020 |
| chrC01 | 11.156 | 17713 |
| chrC01 | 11.635 | 149441 |
| chrC01 | 11.853 | 315548 |
| chrC01 | 12.069 | 290172 |
| chrC01 | 12.411 | 351097 |
| chrC01 | 12.894 | 383343 |
| chrC01 | 14.374 | 391694 |
| chrC01 | 14.777 | 633287 |
| chrC01 | 14.9 | 473498 |
| chrC01 | 15.051 | 660130 |
| chrC01 | 15.518 | 708913 |
| chrC01 | 16.464 | 937731 |
| chrC01 | 16.925 | 1160124 |
| chrC01 | 17.086 | 937805 |
| chrC01 | 17.457 | 1176756 |
| chrC01 | 17.744 | 1129451 |
| chrC01 | 17.861 | 1179255 |
| chrC01 | 19.128 | 1239322 |
| chrC01 | 20.886 | 1349184 |
| chrC01 | 20.958 | 27188012 |
| chrC01 | 21.102 | 1449141 |
| chrC01 | 21.48 | 1588431 |
| chrC01 | 21.792 | 1766487 |
| chrC01 | 22.544 | 1846031 |
| chrC01 | 23.556 | 1942594 |
| chrC01 | 23.556 | 1932688 |
| chrC01 | 23.663 | 1943598 |
| chrC01 | 24.11 | 1926146 |
| chrC01 | 24.306 | 2035342 |
| chrC01 | 24.496 | 2047283 |
| chrC01 | 27.921 | 2159783 |
| chrC01 | 28.238 | 2238075 |
| chrC01 | 29.449 | 2411930 |
| chrC01 | 33.717 | 2576639 |
| chrC01 | 33.88 | 34701353 |
| chrC01 | 34.277 | 2979732 |
| chrC01 | 34.896 | 2937114 |
| chrC01 | 35.653 | 3023228 |
| chrC01 | 35.979 | 3058251 |
| chrC01 | 36.344 | 3075829 |
| chrC01 | 37.253 | 3044683 |
| chrC01 | 37.253 | 3041881 |
| chrC01 | 37.253 | 2133923 |
| chrC01 | 37.253 | 3048630 |
| chrC01 | 37.253 | 3046636 |
| chrC01 | 37.78 | 3176266 |
| chrC01 | 38.231 | 3145430 |
| chrC01 | 38.912 | 3214602 |
| chrC01 | 39.623 | 3253415 |
| chrC01 | 39.756 | 3222216 |
| chrC01 | 40.245 | 3267480 |
| chrC01 | 40.516 | 3291715 |
| chrC01 | 40.688 | 3261816 |
| chrC01 | 42.094 | 20020219 |
| chrC01 | 43.378 | 3432893 |
| chrC01 | 43.609 | 3330925 |
| chrC01 | 43.804 | 3358563 |
| chrC01 | 44.456 | 3484733 |
| chrC01 | 44.681 | 3484956 |
| chrC01 | 46.28 | 3501010 |
| chrC01 | 47.077 | 3935497 |
| chrC01 | 48.199 | 4245969 |
| chrC01 | 50.48 | 4608528 |
| chrC01 | 51.095 | 4955112 |
| chrC01 | 52.021 | 5057105 |
| chrC01 | 54.511 | 5807189 |
| chrC01 | 56.708 | 5958484 |
| chrC01 | 56.979 | 5977834 |
| chrC01 | 60.153 | 7833371 |
| chrC01 | 61.592 | 9006433 |
| chrC01 | 61.732 | 9405801 |
| chrC01 | 68.654 | 14008437 |
| chrC01 | 69.131 | 14512476 |
| chrC01 | 69.693 | 14204451 |
| chrC01 | 69.785 | 14885928 |
| chrC01 | 70.689 | 16093984 |
| chrC01 | 71.347 | 16117010 |
| chrC01 | 71.476 | 16011590 |
| chrC01 | 71.849 | 24058834 |
| chrC01 | 71.849 | 11546857 |
| chrC01 | 71.849 | 6168640 |
| chrC01 | 72.18 | 16876027 |
| chrC01 | 72.275 | 20907131 |
| chrC01 | 72.293 | 19272968 |
| chrC01 | 72.293 | 19271765 |
| chrC01 | 72.6 | 16877535 |
| chrC01 | 72.743 | 19456989 |
| chrC01 | 72.98 | 18961307 |
| chrC01 | 73.038 | 20567258 |
| chrC01 | 73.157 | 25802674 |
| chrC01 | 73.182 | 24162722 |
| chrC01 | 73.351 | 24434914 |
| chrC01 | 73.447 | 24058364 |
| chrC01 | 73.447 | 6168170 |
| chrC01 | 73.447 | 11546387 |
| chrC01 | 73.54 | 17997021 |
| chrC01 | 73.54 | 17631513 |
| chrC01 | 73.54 | 24868435 |
| chrC01 | 73.54 | 24065288 |
| chrC01 | 73.54 | 26337051 |
| chrC01 | 73.896 | 28346163 |
| chrC01 | 74.453 | 29419899 |
| chrC01 | 75.059 | 26681899 |
| chrC01 | 75.938 | 30767309 |
| chrC01 | 76.159 | 31949069 |
| chrC01 | 76.22 | 30869025 |
| chrC01 | 77.114 | 31341373 |
| chrC01 | 77.243 | 31175210 |
| chrC01 | 77.373 | 32442896 |
| chrC01 | 77.747 | 31119419 |
| chrC01 | 77.888 | 31145793 |
| chrC01 | 77.981 | 31089721 |
| chrC01 | 78.936 | 33233288 |
| chrC01 | 79.123 | 32986298 |
| chrC01 | 79.123 | 32985634 |
| chrC01 | 79.945 | 32457868 |
| chrC01 | 82.048 | 34146576 |
| chrC01 | 84.085 | 34418401 |
| chrC01 | 84.743 | 33126176 |
| chrC01 | 85.347 | 34865520 |
| chrC01 | 85.463 | 34720826 |
| chrC01 | 85.783 | 34501582 |
| chrC01 | 86.886 | 35454900 |
| chrC01 | 86.974 | 35053472 |
| chrC01 | 87.491 | 29123779 |
| chrC01 | 88.532 | 31173317 |
| chrC01 | 89.046 | 21324794 |
| chrC01 | 89.046 | 27719667 |
| chrC01 | 90.208 | 35924765 |
| chrC01 | 90.767 | 36212917 |
| chrC01 | 91.539 | 36352675 |
| chrC01 | 91.765 | 36380635 |
| chrC01 | 92.246 | 36483817 |
| chrC01 | 92.426 | 36405920 |
| chrC01 | 93.884 | 37023267 |
| chrC01 | 94.03 | 36987848 |
| chrC01 | 94.582 | 37057609 |
| chrC01 | 94.928 | 37061335 |
| chrC01 | 95.348 | 37065237 |
| chrC02 | 16.255 | 1710308 |
| chrC02 | 19.625 | 1398619 |
| chrC02 | 20.848 | 1168670 |
| chrC02 | 23.678 | 956033 |
| chrC02 | 24.042 | 941968 |
| chrC02 | 24.289 | 834711 |
| chrC02 | 27.233 | 1050870 |
| chrC02 | 29.334 | 524610 |
| chrC02 | 30.971 | 321436 |
| chrC02 | 37.322 | 1889613 |
| chrC02 | 37.702 | 1888634 |
| chrC02 | 40.824 | 2231119 |
| chrC02 | 41.678 | 2289780 |
| chrC02 | 45.359 | 2353791 |
| chrC02 | 51.987 | 2680691 |
| chrC02 | 57.554 | 3213412 |
| chrC02 | 58.466 | 3301804 |
| chrC02 | 59.158 | 3433440 |
| chrC02 | 60.686 | 3466639 |
| chrC02 | 60.686 | 3452489 |
| chrC02 | 61.044 | 3468188 |
| chrC02 | 61.808 | 3545515 |
| chrC02 | 61.958 | 3529405 |
| chrC02 | 63.422 | 3694270 |
| chrC02 | 63.752 | 3637317 |
| chrC02 | 65.264 | 3734035 |
| chrC02 | 66.472 | 4330203 |
| chrC02 | 67.296 | 4466827 |
| chrC02 | 68 | 5044999 |
| chrC02 | 68.96 | 5070177 |
| chrC02 | 70.982 | 5205573 |
| chrC02 | 71.71 | 5172921 |
| chrC02 | 74.801 | 5655165 |
| chrC02 | 75.05 | 5844658 |
| chrC02 | 75.283 | 5904629 |
| chrC02 | 79.545 | 10546717 |
| chrC02 | 80.289 | 10565895 |
| chrC02 | 81.759 | 12315614 |
| chrC02 | 81.759 | 12317484 |
| chrC02 | 81.759 | 12343221 |
| chrC02 | 82.524 | 17328346 |
| chrC02 | 83.019 | 19174503 |
| chrC02 | 84.618 | 16526418 |
| chrC02 | 84.727 | 19516829 |
| chrC02 | 87.059 | 26442805 |
| chrC02 | 87.627 | 20113617 |
| chrC02 | 88.225 | 25784707 |
| chrC02 | 88.225 | 28841529 |
| chrC02 | 90.03 | 35859371 |
| chrC02 | 90.325 | 35936544 |
| chrC02 | 90.325 | 35996628 |
| chrC02 | 90.666 | 36329706 |
| chrC02 | 93.125 | 37242005 |
| chrC02 | 93.125 | 37242386 |
| chrC02 | 93.125 | 37242259 |
| chrC02 | 93.125 | 37242132 |
| chrC02 | 94.354 | 37975587 |
| chrC02 | 94.479 | 37966983 |
| chrC02 | 95.477 | 39027367 |
| chrC02 | 95.603 | 39004007 |
| chrC02 | 100.147 | 40004041 |
| chrC02 | 101.097 | 40354850 |
| chrC02 | 102.176 | 40618553 |
| chrC02 | 105.389 | 41640004 |
| chrC02 | 106.051 | 41763341 |
| chrC02 | 113.609 | 42864339 |
| chrC02 | 115.19 | 42953569 |
| chrC02 | 117.423 | 43542592 |
| chrC02 | 119.797 | 43788040 |
| chrC02 | 121.722 | 44036877 |
| chrC02 | 121.997 | 44061731 |
| chrC02 | 122.194 | 44012952 |
| chrC02 | 122.918 | 44139464 |
| chrC02 | 123.247 | 44041705 |
| chrC02 | 123.699 | 44366336 |
| chrC02 | 124.112 | 44140921 |
| chrC02 | 124.112 | 25011856 |
| chrC02 | 125.653 | 44435327 |
| chrC02 | 130.855 | 44920482 |
| chrC02 | 131.172 | 45062402 |
| chrC02 | 132.754 | 45156042 |
| chrC02 | 133.383 | 45254270 |
| chrC02 | 133.779 | 45273432 |
| chrC02 | 134.129 | 45208012 |
| chrC02 | 136.129 | 45481993 |
| chrC02 | 137.212 | 45588398 |
| chrC02 | 138.375 | 45788197 |
| chrC02 | 139.766 | 12237509 |
| chrC03 | 11.628 | 79686 |
| chrC03 | 13.022 | 384494 |
| chrC03 | 14.719 | 520231 |
| chrC03 | 15.391 | 633757 |
| chrC03 | 16.755 | 745713 |
| chrC03 | 18.074 | 793806 |
| chrC03 | 19.944 | 915452 |
| chrC03 | 20.547 | 915986 |
| chrC03 | 22.141 | 1188042 |
| chrC03 | 22.439 | 1592946 |
| chrC03 | 22.628 | 1462134 |
| chrC03 | 24.445 | 1429147 |
| chrC03 | 24.605 | 1428546 |
| chrC03 | 25.709 | 1688449 |
| chrC03 | 32.064 | 2630227 |
| chrC03 | 35.686 | 2894880 |
| chrC03 | 41.016 | 3386159 |
| chrC03 | 43.176 | 4060771 |
| chrC03 | 44.972 | 4316287 |
| chrC03 | 45.669 | 4427828 |
| chrC03 | 46.43 | 4747594 |
| chrC03 | 47.322 | 5014249 |
| chrC03 | 47.596 | 5083368 |
| chrC03 | 47.596 | 5083308 |
| chrC03 | 47.702 | 5090173 |
| chrC03 | 48.232 | 5106768 |
| chrC03 | 49.354 | 5610941 |
| chrC03 | 50.192 | 5736022 |
| chrC03 | 50.467 | 5770315 |
| chrC03 | 50.551 | 5901012 |
| chrC03 | 50.906 | 5981138 |
| chrC03 | 51.758 | 6166223 |
| chrC03 | 52.008 | 6151122 |
| chrC03 | 53.096 | 6205488 |
| chrC03 | 53.226 | 6256926 |
| chrC03 | 53.791 | 6366585 |
| chrC03 | 53.971 | 6352340 |
| chrC03 | 54.293 | 6353489 |
| chrC03 | 55.657 | 6557527 |
| chrC03 | 57.654 | 6744282 |
| chrC03 | 58.114 | 6792030 |
| chrC03 | 58.372 | 6816025 |
| chrC03 | 58.86 | 6837075 |
| chrC03 | 60.38 | 6924425 |
| chrC03 | 63.4 | 7704425 |
| chrC03 | 66.311 | 8279339 |
| chrC03 | 66.568 | 7964476 |
| chrC03 | 66.615 | 7808207 |
| chrC03 | 66.684 | 8037743 |
| chrC03 | 66.868 | 8301496 |
| chrC03 | 67.053 | 8009880 |
| chrC03 | 67.235 | 8408926 |
| chrC03 | 67.572 | 8554482 |
| chrC03 | 67.984 | 8563528 |
| chrC03 | 69.877 | 9031466 |
| chrC03 | 70.316 | 9150063 |
| chrC03 | 71.918 | 9753450 |
| chrC03 | 72.777 | 10196831 |
| chrC03 | 72.849 | 10366535 |
| chrC03 | 72.849 | 31775825 |
| chrC03 | 73.11 | 10516422 |
| chrC03 | 73.229 | 10765547 |
| chrC03 | 73.534 | 10943949 |
| chrC03 | 73.848 | 11039969 |
| chrC03 | 74.341 | 11470318 |
| chrC03 | 74.466 | 11470236 |
| chrC03 | 74.634 | 11587606 |
| chrC03 | 74.738 | 11576800 |
| chrC03 | 75.398 | 11865680 |
| chrC03 | 75.746 | 12057100 |
| chrC03 | 78.162 | 13022159 |
| chrC03 | 78.423 | 13076884 |
| chrC03 | 78.579 | 13095261 |
| chrC03 | 79.421 | 13854802 |
| chrC03 | 79.514 | 13854699 |
| chrC03 | 79.692 | 13594814 |
| chrC03 | 80.109 | 14302633 |
| chrC03 | 80.109 | 14290455 |
| chrC03 | 80.168 | 14101071 |
| chrC03 | 81.182 | 14789318 |
| chrC03 | 84.26 | 15145611 |
| chrC03 | 84.65 | 15136056 |
| chrC03 | 85.244 | 15388261 |
| chrC03 | 85.832 | 15959827 |
| chrC03 | 86.245 | 15983302 |
| chrC03 | 87.48 | 16291143 |
| chrC03 | 87.718 | 16369510 |
| chrC03 | 88.373 | 16530245 |
| chrC03 | 89.704 | 16759347 |
| chrC03 | 91.513 | 16783821 |
| chrC03 | 91.642 | 16783722 |
| chrC03 | 94.189 | 17416147 |
| chrC03 | 94.428 | 17416201 |
| chrC03 | 94.614 | 17382502 |
| chrC03 | 94.614 | 35252424 |
| chrC03 | 95.367 | 18075400 |
| chrC03 | 95.575 | 18181893 |
| chrC03 | 100.367 | 19626774 |
| chrC03 | 100.585 | 19752749 |
| chrC03 | 101.41 | 19920638 |
| chrC03 | 101.653 | 19920462 |
| chrC03 | 101.968 | 20104846 |
| chrC03 | 102.371 | 20430179 |
| chrC03 | 105.29 | 20727067 |
| chrC03 | 106.136 | 20735322 |
| chrC03 | 106.782 | 20809474 |
| chrC03 | 107.45 | 20856093 |
| chrC03 | 111.941 | 21252044 |
| chrC03 | 112.223 | 21271957 |
| chrC03 | 112.48 | 28788072 |
| chrC03 | 113.283 | 21576958 |
| chrC03 | 113.283 | 46810847 |
| chrC03 | 114.438 | 21660003 |
| chrC03 | 114.438 | 22435311 |
| chrC03 | 114.931 | 21699129 |
| chrC03 | 115.603 | 21720267 |
| chrC03 | 115.932 | 21828759 |
| chrC03 | 116.22 | 21742715 |
| chrC03 | 117.493 | 21921252 |
| chrC03 | 117.68 | 21917967 |
| chrC03 | 118.035 | 22065623 |
| chrC03 | 122.154 | 23167004 |
| chrC03 | 122.424 | 23195081 |
| chrC03 | 122.569 | 23201270 |
| chrC03 | 124.168 | 23356791 |
| chrC03 | 124.561 | 23421740 |
| chrC03 | 125.235 | 23453910 |
| chrC03 | 126.581 | 24045454 |
| chrC03 | 126.842 | 24603562 |
| chrC03 | 127.028 | 24394600 |
| chrC03 | 127.981 | 24897397 |
| chrC03 | 128.279 | 25966141 |
| chrC03 | 128.428 | 26268129 |
| chrC03 | 128.583 | 25998316 |
| chrC03 | 129.22 | 27368326 |
| chrC03 | 129.468 | 26991220 |
| chrC03 | 130.03 | 27641539 |
| chrC03 | 130.097 | 28139071 |
| chrC03 | 130.248 | 28917182 |
| chrC03 | 130.516 | 28561186 |
| chrC03 | 130.788 | 29919328 |
| chrC03 | 130.919 | 28997047 |
| chrC03 | 131.209 | 29082988 |
| chrC03 | 131.209 | 29077992 |
| chrC03 | 134.627 | 30174939 |
| chrC03 | 135.436 | 30385401 |
| chrC03 | 136.504 | 31617273 |
| chrC03 | 138.105 | 32548505 |
| chrC03 | 138.283 | 32498585 |
| chrC03 | 139.114 | 57662161 |
| chrC03 | 142.21 | 34607669 |
| chrC03 | 142.654 | 34629668 |
| chrC03 | 143.14 | 34660961 |
| chrC03 | 145.579 | 34834525 |
| chrC03 | 146.701 | 34921707 |
| chrC03 | 146.912 | 34956166 |
| chrC03 | 146.912 | 34956886 |
| chrC03 | 147.349 | 34982792 |
| chrC03 | 147.883 | 35057380 |
| chrC03 | 148.215 | 35064146 |
| chrC03 | 149.879 | 26643966 |
| chrC03 | 149.879 | 27023189 |
| chrC03 | 150.002 | 40962137 |
| chrC03 | 150.002 | 40964842 |
| chrC03 | 150.002 | 40967548 |
| chrC03 | 150.002 | 38350284 |
| chrC03 | 150.002 | 39575851 |
| chrC03 | 150.002 | 41688465 |
| chrC03 | 150.002 | 41432004 |
| chrC03 | 150.002 | 39688183 |
| chrC03 | 150.439 | 46272413 |
| chrC03 | 150.532 | 46238447 |
| chrC03 | 150.775 | 46451164 |
| chrC03 | 150.775 | 46455925 |
| chrC03 | 156.977 | 47870268 |
| chrC03 | 156.977 | 47865306 |
| chrC03 | 156.977 | 9638445 |
| chrC03 | 157.403 | 48212826 |
| chrC03 | 157.81 | 48165790 |
| chrC03 | 158.712 | 48738601 |
| chrC03 | 160.014 | 13082638 |
| chrC03 | 160.249 | 48400476 |
| chrC03 | 160.249 | 48356483 |
| chrC03 | 160.536 | 48417741 |
| chrC03 | 160.714 | 48845720 |
| chrC03 | 161.604 | 49124421 |
| chrC03 | 161.908 | 49183976 |
| chrC03 | 161.996 | 49139081 |
| chrC03 | 164.25 | 49244733 |
| chrC03 | 165.712 | 49306090 |
| chrC03 | 166.256 | 49313499 |
| chrC03 | 169.82 | 49528253 |
| chrC03 | 170.461 | 49615786 |
| chrC03 | 171.307 | 50637773 |
| chrC03 | 171.307 | 26942811 |
| chrC03 | 171.307 | 50640185 |
| chrC03 | 171.474 | 50239315 |
| chrC03 | 171.74 | 50228680 |
| chrC03 | 172.172 | 50797722 |
| chrC03 | 173.642 | 51428044 |
| chrC03 | 174.758 | 52352774 |
| chrC03 | 176.046 | 52628194 |
| chrC03 | 176.323 | 52632208 |
| chrC03 | 177.388 | 53438359 |
| chrC03 | 178.297 | 53440872 |
| chrC03 | 178.885 | 53459675 |
| chrC03 | 179.796 | 53832971 |
| chrC03 | 182.284 | 55416353 |
| chrC03 | 182.525 | 55449004 |
| chrC03 | 182.945 | 55713230 |
| chrC03 | 183.542 | 55837809 |
| chrC03 | 187.961 | 56375332 |
| chrC03 | 188.183 | 56642199 |
| chrC03 | 188.633 | 56649514 |
| chrC03 | 203.154 | 58524673 |
| chrC03 | 204.98 | 58685925 |
| chrC03 | 205.494 | 59242219 |
| chrC03 | 206.569 | 19344980 |
| chrC03 | 207.994 | 59606851 |
| chrC03 | 210.263 | 60269071 |
| chrC03 | 211.106 | 60556851 |
| chrC04 | 3.04 | 987180 |
| chrC04 | 9.085 | 2669484 |
| chrC04 | 27.262 | 39654 |
| chrC04 | 29.594 | 378537 |
| chrC04 | 32.13 | 1016253 |
| chrC04 | 32.419 | 1008327 |
| chrC04 | 32.78 | 1146332 |
| chrC04 | 35.954 | 1687266 |
| chrC04 | 39.668 | 2114648 |
| chrC04 | 44.134 | 2412830 |
| chrC04 | 44.847 | 2568988 |
| chrC04 | 45.083 | 42754616 |
| chrC04 | 46.213 | 42847326 |
| chrC04 | 46.213 | 10290369 |
| chrC04 | 46.795 | 44387135 |
| chrC04 | 48.987 | 3775770 |
| chrC04 | 49.141 | 3730388 |
| chrC04 | 49.725 | 3849317 |
| chrC04 | 49.924 | 4097464 |
| chrC04 | 50.95 | 44458531 |
| chrC04 | 51.369 | 4146137 |
| chrC04 | 51.656 | 44459192 |
| chrC04 | 51.656 | 7280570 |
| chrC04 | 52.037 | 4422993 |
| chrC04 | 52.037 | 45711717 |
| chrC04 | 52.065 | 44735890 |
| chrC04 | 52.173 | 4437607 |
| chrC04 | 53.334 | 4548300 |
| chrC04 | 56.042 | 5505778 |
| chrC04 | 56.22 | 5444202 |
| chrC04 | 56.504 | 5700964 |
| chrC04 | 57.287 | 5714743 |
| chrC04 | 57.799 | 5915633 |
| chrC04 | 58.227 | 5915413 |
| chrC04 | 59.695 | 5995503 |
| chrC04 | 60.465 | 44902677 |
| chrC04 | 62.46 | 6511970 |
| chrC04 | 64.19 | 7269345 |
| chrC04 | 64.429 | 7402792 |
| chrC04 | 65.058 | 7866955 |
| chrC04 | 69.262 | 8321985 |
| chrC04 | 77.05 | 9155967 |
| chrC04 | 77.642 | 9140950 |
| chrC04 | 78.736 | 11093468 |
| chrC04 | 79.198 | 21513003 |
| chrC04 | 79.634 | 11252192 |
| chrC04 | 79.666 | 11389497 |
| chrC04 | 79.848 | 17204471 |
| chrC04 | 79.96 | 18885107 |
| chrC04 | 81.215 | 18931768 |
| chrC04 | 81.3 | 20412523 |
| chrC04 | 81.419 | 16454154 |
| chrC04 | 82.517 | 23202238 |
| chrC04 | 82.733 | 24597748 |
| chrC04 | 83.268 | 25675064 |
| chrC04 | 83.638 | 25869831 |
| chrC04 | 83.978 | 26191922 |
| chrC04 | 83.978 | 31666701 |
| chrC04 | 83.978 | 22386082 |
| chrC04 | 86.31 | 28353425 |
| chrC04 | 87.288 | 28593432 |
| chrC04 | 87.832 | 29087244 |
| chrC04 | 88.349 | 29725852 |
| chrC04 | 89.982 | 30231837 |
| chrC04 | 90.426 | 29899208 |
| chrC04 | 91.964 | 31237280 |
| chrC04 | 95.215 | 32912811 |
| chrC04 | 95.441 | 32746468 |
| chrC04 | 95.543 | 32793646 |
| chrC04 | 95.943 | 33320812 |
| chrC04 | 97.255 | 33502264 |
| chrC04 | 98.284 | 33924212 |
| chrC04 | 98.748 | 34524357 |
| chrC04 | 98.951 | 34318859 |
| chrC04 | 98.951 | 4724040 |
| chrC04 | 99.311 | 34224532 |
| chrC04 | 99.703 | 34602069 |
| chrC04 | 100.152 | 38831542 |
| chrC04 | 100.615 | 38834055 |
| chrC04 | 106.389 | 36174462 |
| chrC04 | 108.833 | 39041945 |
| chrC04 | 121.927 | 44749447 |
| chrC04 | 122.118 | 44512595 |
| chrC04 | 124.625 | 44901668 |
| chrC04 | 124.625 | 5984550 |
| chrC04 | 127.881 | 45278052 |
| chrC04 | 130.046 | 45550115 |
| chrC04 | 130.166 | 45737046 |
| chrC04 | 131.489 | 45789987 |
| chrC04 | 134.83 | 46171876 |
| chrC04 | 135.049 | 46169197 |
| chrC04 | 138.151 | 46413960 |
| chrC04 | 139.768 | 46736913 |
| chrC04 | 139.768 | 1662096 |
| chrC04 | 141.749 | 47079855 |
| chrC04 | 142.465 | 47081924 |
| chrC04 | 142.812 | 47096371 |
| chrC04 | 144.173 | 47356669 |
| chrC04 | 144.446 | 47642548 |
| chrC04 | 144.699 | 47557796 |
| chrC04 | 145.251 | 47556494 |
| chrC04 | 145.846 | 47881508 |
| chrC04 | 147.268 | 48114379 |
| chrC04 | 147.392 | 48252199 |
| chrC04 | 147.861 | 48265347 |
| chrC04 | 148.295 | 48843988 |
| chrC04 | 148.295 | 478882 |
| chrC05 | 10.997 | 663395 |
| chrC05 | 11.23 | 410195 |
| chrC05 | 14.909 | 1042737 |
| chrC05 | 24.789 | 1541945 |
| chrC05 | 26.379 | 1974023 |
| chrC05 | 31.341 | 2295935 |
| chrC05 | 32.829 | 2481580 |
| chrC05 | 34.624 | 2915080 |
| chrC05 | 36.559 | 3036589 |
| chrC05 | 37.003 | 3089083 |
| chrC05 | 37.339 | 3079271 |
| chrC05 | 38.083 | 3134032 |
| chrC05 | 38.472 | 3138066 |
| chrC05 | 42.556 | 3663234 |
| chrC05 | 43.38 | 4042772 |
| chrC05 | 43.699 | 4112154 |
| chrC05 | 49.293 | 4370752 |
| chrC05 | 50.41 | 4668174 |
| chrC05 | 50.66 | 4889001 |
| chrC05 | 51.107 | 5123353 |
| chrC05 | 52.606 | 5444308 |
| chrC05 | 54.766 | 5890237 |
| chrC05 | 55.428 | 6102359 |
| chrC05 | 55.9 | 6103057 |
| chrC05 | 56.085 | 6144087 |
| chrC05 | 63.341 | 7059296 |
| chrC05 | 63.817 | 7041835 |
| chrC05 | 66.615 | 8098463 |
| chrC05 | 68.301 | 9283183 |
| chrC05 | 70.928 | 11182837 |
| chrC05 | 71.327 | 11066385 |
| chrC05 | 71.816 | 11618545 |
| chrC05 | 72.392 | 12153209 |
| chrC05 | 72.621 | 12569081 |
| chrC05 | 72.717 | 12356387 |
| chrC05 | 72.802 | 11637904 |
| chrC05 | 73.008 | 13299376 |
| chrC05 | 73.354 | 14790425 |
| chrC05 | 74.095 | 21774827 |
| chrC05 | 74.487 | 24247482 |
| chrC05 | 74.68 | 22519407 |
| chrC05 | 76.436 | 29639133 |
| chrC05 | 78.712 | 31885294 |
| chrC05 | 85.67 | 35621673 |
| chrC05 | 90.345 | 36474795 |
| chrC05 | 91.309 | 37061631 |
| chrC05 | 91.56 | 36995534 |
| chrC05 | 92.81 | 38213420 |
| chrC05 | 93.085 | 38002799 |
| chrC05 | 93.739 | 40627194 |
| chrC05 | 93.739 | 19831464 |
| chrC05 | 93.739 | 10839368 |
| chrC05 | 96.992 | 39465937 |
| chrC05 | 101.925 | 39828531 |
| chrC05 | 102.977 | 39828361 |
| chrC05 | 104.488 | 40095783 |
| chrC05 | 105.606 | 40110645 |
| chrC05 | 105.759 | 40111558 |
| chrC05 | 105.895 | 40064547 |
| chrC05 | 106.003 | 40106725 |
| chrC05 | 106.859 | 28198367 |
| chrC05 | 107.034 | 39517303 |
| chrC05 | 107.332 | 40211059 |
| chrC05 | 107.332 | 40232286 |
| chrC05 | 107.582 | 39517547 |
| chrC05 | 107.677 | 40252159 |
| chrC05 | 107.973 | 39499952 |
| chrC05 | 108.078 | 40267317 |
| chrC05 | 108.998 | 39703911 |
| chrC05 | 109.209 | 40279351 |
| chrC05 | 109.226 | 39623515 |
| chrC05 | 110.348 | 40356044 |
| chrC05 | 110.565 | 39846482 |
| chrC05 | 110.959 | 40421131 |
| chrC05 | 113.324 | 40203272 |
| chrC05 | 114.025 | 40270779 |
| chrC05 | 114.543 | 40280616 |
| chrC05 | 116.778 | 42767130 |
| chrC05 | 117.048 | 40519398 |
| chrC05 | 117.445 | 40556603 |
| chrC05 | 117.587 | 40600452 |
| chrC05 | 117.796 | 40729771 |
| chrC05 | 118.234 | 40995727 |
| chrC05 | 118.514 | 40789063 |
| chrC05 | 119.03 | 41019906 |
| chrC05 | 119.336 | 40834960 |
| chrC05 | 119.684 | 41020963 |
| chrC05 | 120.32 | 41120129 |
| chrC05 | 120.493 | 41147712 |
| chrC05 | 120.676 | 41126225 |
| chrC05 | 121.37 | 41341002 |
| chrC05 | 121.408 | 41128778 |
| chrC05 | 121.67 | 41201864 |
| chrC05 | 122.122 | 41605155 |
| chrC05 | 122.169 | 41748176 |
| chrC05 | 122.401 | 41549806 |
| chrC05 | 122.507 | 42586952 |
| chrC05 | 122.853 | 41648301 |
| chrC05 | 122.853 | 41644404 |
| chrC05 | 122.911 | 42608808 |
| chrC05 | 123.155 | 41922498 |
| chrC05 | 123.275 | 41750625 |
| chrC05 | 124.224 | 41751906 |
| chrC05 | 124.36 | 42034508 |
| chrC05 | 124.459 | 41568365 |
| chrC05 | 126.574 | 41676682 |
| chrC05 | 128.185 | 43072310 |
| chrC05 | 129.875 | 42914285 |
| chrC06 | 0.363 | 779231 |
| chrC06 | 0.715 | 2526747 |
| chrC06 | 1.07 | 2651944 |
| chrC06 | 2.12 | 2691479 |
| chrC06 | 3.181 | 2867934 |
| chrC06 | 7.503 | 3538492 |
| chrC06 | 10.093 | 3594287 |
| chrC06 | 13.039 | 3757343 |
| chrC06 | 13.651 | 3803284 |
| chrC06 | 15.779 | 12845007 |
| chrC06 | 20.48 | 5909599 |
| chrC06 | 22.712 | 5956879 |
| chrC06 | 26.568 | 7267262 |
| chrC06 | 27.317 | 9303073 |
| chrC06 | 27.391 | 22587697 |
| chrC06 | 27.391 | 8489011 |
| chrC06 | 27.585 | 9030029 |
| chrC06 | 27.685 | 29186433 |
| chrC06 | 28.442 | 11594844 |
| chrC06 | 28.524 | 11322251 |
| chrC06 | 30.236 | 12388187 |
| chrC06 | 30.72 | 12628344 |
| chrC06 | 31.138 | 13124501 |
| chrC06 | 31.627 | 13088794 |
| chrC06 | 31.627 | 4367066 |
| chrC06 | 34.059 | 15667718 |
| chrC06 | 40.047 | 17527907 |
| chrC06 | 40.453 | 17917417 |
| chrC06 | 42.753 | 18727237 |
| chrC06 | 55.624 | 20461981 |
| chrC06 | 56.009 | 20447532 |
| chrC06 | 59.192 | 21808033 |
| chrC06 | 59.922 | 21784608 |
| chrC06 | 60.421 | 21936544 |
| chrC06 | 61.286 | 32300990 |
| chrC06 | 61.74 | 29489396 |
| chrC06 | 62.345 | 32322672 |
| chrC06 | 62.942 | 24561889 |
| chrC06 | 63.356 | 31183235 |
| chrC06 | 63.5 | 30594719 |
| chrC06 | 63.656 | 29296939 |
| chrC06 | 63.74 | 34225439 |
| chrC06 | 63.74 | 26085812 |
| chrC06 | 63.868 | 26260417 |
| chrC06 | 63.918 | 29269840 |
| chrC06 | 63.994 | 26531086 |
| chrC06 | 64.044 | 28381763 |
| chrC06 | 64.346 | 32291517 |
| chrC06 | 64.396 | 27808921 |
| chrC06 | 64.498 | 30163669 |
| chrC06 | 64.581 | 31005851 |
| chrC06 | 64.593 | 30778455 |
| chrC06 | 64.616 | 27537217 |
| chrC06 | 64.645 | 32807211 |
| chrC06 | 64.691 | 30594706 |
| chrC06 | 64.726 | 31221279 |
| chrC06 | 64.743 | 31340690 |
| chrC06 | 64.759 | 27661953 |
| chrC06 | 64.785 | 27573561 |
| chrC06 | 64.832 | 27542144 |
| chrC06 | 64.89 | 32252169 |
| chrC06 | 64.953 | 32252051 |
| chrC06 | 65.026 | 29530428 |
| chrC06 | 65.082 | 32884173 |
| chrC06 | 65.125 | 33255277 |
| chrC06 | 65.171 | 33242057 |
| chrC06 | 65.187 | 33247657 |
| chrC06 | 65.215 | 33224613 |
| chrC06 | 65.244 | 32985412 |
| chrC06 | 65.285 | 33220806 |
| chrC06 | 65.479 | 33540304 |
| chrC06 | 65.519 | 33582709 |
| chrC06 | 65.578 | 33601823 |
| chrC06 | 65.745 | 30124797 |
| chrC06 | 65.901 | 33566836 |
| chrC06 | 66.195 | 33802614 |
| chrC06 | 66.239 | 33962786 |
| chrC06 | 66.292 | 34011622 |
| chrC06 | 66.334 | 34131345 |
| chrC06 | 66.463 | 34051692 |
| chrC06 | 66.637 | 34080557 |
| chrC06 | 66.821 | 34145532 |
| chrC06 | 66.902 | 34145834 |
| chrC06 | 67.099 | 34271927 |
| chrC06 | 67.22 | 34227735 |
| chrC06 | 67.307 | 34225192 |
| chrC06 | 67.459 | 34488599 |
| chrC06 | 67.567 | 34331807 |
| chrC06 | 67.699 | 34488820 |
| chrC06 | 68.931 | 34620879 |
| chrC06 | 69.092 | 34647828 |
| chrC06 | 69.354 | 34620733 |
| chrC06 | 69.626 | 34617914 |
| chrC06 | 71.273 | 35121123 |
| chrC06 | 71.542 | 35128967 |
| chrC06 | 71.767 | 35275761 |
| chrC06 | 71.767 | 35271793 |
| chrC06 | 71.898 | 35069486 |
| chrC06 | 71.97 | 35281130 |
| chrC06 | 72.363 | 35673300 |
| chrC06 | 72.542 | 35425606 |
| chrC06 | 72.654 | 35323423 |
| chrC06 | 72.741 | 35423017 |
| chrC06 | 72.87 | 35520279 |
| chrC06 | 73.058 | 35686326 |
| chrC06 | 73.148 | 35686873 |
| chrC06 | 73.713 | 35895720 |
| chrC06 | 74.028 | 35732961 |
| chrC06 | 74.133 | 35755536 |
| chrC06 | 74.363 | 35872417 |
| chrC06 | 74.405 | 35750619 |
| chrC06 | 74.58 | 35936930 |
| chrC06 | 74.676 | 35707038 |
| chrC06 | 74.836 | 36174062 |
| chrC06 | 74.947 | 36233436 |
| chrC06 | 75.102 | 36238471 |
| chrC06 | 75.198 | 36036016 |
| chrC06 | 75.511 | 36178500 |
| chrC06 | 95.392 | 36317912 |
| chrC06 | 102.388 | 35009971 |
| chrC07 | 0.358 | 20749192 |
| chrC07 | 2.729 | 22343368 |
| chrC07 | 12.901 | 26700733 |
| chrC07 | 13.317 | 26892277 |
| chrC07 | 13.742 | 26905746 |
| chrC07 | 18.311 | 27776865 |
| chrC07 | 20.406 | 27983458 |
| chrC07 | 21.101 | 28169337 |
| chrC07 | 21.89 | 28348697 |
| chrC07 | 22.986 | 28446712 |
| chrC07 | 24.71 | 28502236 |
| chrC07 | 24.975 | 28665046 |
| chrC07 | 27.626 | 34324931 |
| chrC07 | 27.795 | 30054396 |
| chrC07 | 29.65 | 30813034 |
| chrC07 | 29.65 | 17035341 |
| chrC07 | 29.905 | 31134036 |
| chrC07 | 29.905 | 31133878 |
| chrC07 | 30.325 | 31112325 |
| chrC07 | 31.825 | 32074957 |
| chrC07 | 32.448 | 32184623 |
| chrC07 | 33.775 | 32309304 |
| chrC07 | 34.465 | 32477633 |
| chrC07 | 35.222 | 32521704 |
| chrC07 | 35.639 | 32607821 |
| chrC07 | 36.199 | 32627238 |
| chrC07 | 36.324 | 32685609 |
| chrC07 | 38.016 | 33143985 |
| chrC07 | 38.62 | 33111757 |
| chrC07 | 38.888 | 33169166 |
| chrC07 | 39.649 | 33445193 |
| chrC07 | 41.806 | 33958238 |
| chrC07 | 43.607 | 34553685 |
| chrC07 | 43.931 | 34730780 |
| chrC07 | 45.015 | 34913570 |
| chrC07 | 45.341 | 34945155 |
| chrC07 | 45.703 | 35006391 |
| chrC07 | 46.406 | 35018733 |
| chrC07 | 48.127 | 35244476 |
| chrC07 | 48.502 | 35330026 |
| chrC07 | 48.83 | 35449226 |
| chrC07 | 50.257 | 35562643 |
| chrC07 | 50.632 | 35844290 |
| chrC07 | 50.632 | 35842911 |
| chrC07 | 51.729 | 35902688 |
| chrC07 | 52.583 | 36134224 |
| chrC07 | 54.027 | 36246810 |
| chrC07 | 56.365 | 36781083 |
| chrC07 | 56.677 | 36740037 |
| chrC07 | 58.326 | 37620552 |
| chrC07 | 59.354 | 37647605 |
| chrC07 | 62.063 | 38583699 |
| chrC07 | 62.201 | 38400022 |
| chrC07 | 65.193 | 39846039 |
| chrC07 | 65.308 | 39869272 |
| chrC07 | 66.355 | 40295823 |
| chrC07 | 72.276 | 40824750 |
| chrC07 | 75.993 | 41563500 |
| chrC07 | 76.115 | 41612433 |
| chrC07 | 80.154 | 41851570 |
| chrC07 | 80.154 | 41864794 |
| chrC07 | 81.793 | 42283634 |
| chrC07 | 81.969 | 42269058 |
| chrC07 | 82.64 | 42597483 |
| chrC07 | 84.406 | 42607273 |
| chrC07 | 85.512 | 42946828 |
| chrC07 | 88.696 | 43563074 |
| chrC07 | 88.948 | 43620589 |
| chrC07 | 90.507 | 43751379 |
| chrC07 | 91.811 | 44315252 |
| chrC07 | 92.13 | 44476209 |
| chrC08 | 13.588 | 13488725 |
| chrC08 | 14.129 | 12527809 |
| chrC08 | 14.307 | 16269359 |
| chrC08 | 14.567 | 14856679 |
| chrC08 | 14.643 | 9089957 |
| chrC08 | 14.875 | 4583596 |
| chrC08 | 15.097 | 5669907 |
| chrC08 | 15.367 | 3517730 |
| chrC08 | 15.367 | 3605223 |
| chrC08 | 15.989 | 1618829 |
| chrC08 | 16.218 | 1390576 |
| chrC08 | 16.514 | 1364372 |
| chrC08 | 32.246 | 19676265 |
| chrC08 | 32.246 | 36218464 |
| chrC08 | 32.599 | 19853930 |
| chrC08 | 32.69 | 19875191 |
| chrC08 | 34.387 | 20070946 |
| chrC08 | 40.97 | 20654120 |
| chrC08 | 41.69 | 20727121 |
| chrC08 | 41.813 | 20726993 |
| chrC08 | 56.563 | 24629540 |
| chrC08 | 56.807 | 24936913 |
| chrC08 | 60.76 | 25380953 |
| chrC08 | 61.645 | 25496683 |
| chrC08 | 61.84 | 25496879 |
| chrC08 | 62.522 | 26076899 |
| chrC08 | 65.37 | 26723477 |
| chrC08 | 66.651 | 26985890 |
| chrC08 | 67.214 | 27057061 |
| chrC08 | 67.214 | 27061738 |
| chrC08 | 70.345 | 27320499 |
| chrC08 | 71.344 | 27578903 |
| chrC08 | 71.507 | 27492196 |
| chrC08 | 72.154 | 27758728 |
| chrC08 | 72.512 | 28110709 |
| chrC08 | 72.717 | 27792698 |
| chrC08 | 74.944 | 28604451 |
| chrC08 | 76.345 | 28964251 |
| chrC08 | 76.636 | 29226881 |
| chrC08 | 76.636 | 30257151 |
| chrC08 | 76.83 | 29099118 |
| chrC08 | 77.225 | 29297999 |
| chrC08 | 90.932 | 33400961 |
| chrC08 | 91.026 | 33371548 |
| chrC08 | 91.818 | 33565981 |
| chrC08 | 92.606 | 33466316 |
| chrC08 | 95.451 | 34281900 |
| chrC08 | 95.855 | 34657191 |
| chrC08 | 96.29 | 34272109 |
| chrC08 | 97.918 | 37177268 |
| chrC08 | 98.463 | 34991950 |
| chrC08 | 98.746 | 36991239 |
| chrC08 | 99.127 | 34979041 |
| chrC08 | 99.481 | 36743549 |
| chrC08 | 99.834 | 35765664 |
| chrC08 | 100.719 | 37630452 |
| chrC08 | 100.76 | 37891725 |
| chrC08 | 100.914 | 36034597 |
| chrC08 | 101.242 | 37001111 |
| chrC08 | 101.335 | 37667324 |
| chrC08 | 101.55 | 36223666 |
| chrC08 | 101.68 | 37983810 |
| chrC08 | 101.78 | 36994812 |
| chrC08 | 101.894 | 37592319 |
| chrC08 | 101.924 | 36482495 |
| chrC08 | 101.995 | 36471752 |
| chrC08 | 102.034 | 36471904 |
| chrC08 | 102.123 | 36510677 |
| chrC08 | 102.257 | 37497097 |
| chrC08 | 102.331 | 37692266 |
| chrC08 | 102.5 | 36515187 |
| chrC08 | 102.52 | 37343999 |
| chrC08 | 102.52 | 37342870 |
| chrC08 | 102.677 | 37788442 |
| chrC08 | 102.695 | 36478371 |
| chrC08 | 103.035 | 36063579 |
| chrC08 | 106.416 | 37373265 |
| chrC08 | 107.702 | 37348431 |
| chrC08 | 108.776 | 38305421 |
| chrC08 | 115.582 | 37481913 |
| chrC08 | 116.191 | 37480930 |
| chrC09 | 13.29 | 1679915 |
| chrC09 | 16.004 | 68200 |
| chrC09 | 16.847 | 980094 |
| chrC09 | 17.085 | 414762 |
| chrC09 | 17.387 | 728470 |
| chrC09 | 17.631 | 733560 |
| chrC09 | 17.729 | 816308 |
| chrC09 | 17.845 | 813295 |
| chrC09 | 17.902 | 813571 |
| chrC09 | 17.965 | 814049 |
| chrC09 | 18.124 | 866181 |
| chrC09 | 18.202 | 984797 |
| chrC09 | 18.333 | 938236 |
| chrC09 | 18.472 | 994574 |
| chrC09 | 19.208 | 1145357 |
| chrC09 | 19.355 | 1338793 |
| chrC09 | 19.565 | 1314542 |
| chrC09 | 20.112 | 1488233 |
| chrC09 | 20.355 | 1753062 |
| chrC09 | 20.717 | 1584198 |
| chrC09 | 21.079 | 1807377 |
| chrC09 | 21.685 | 33318123 |
| chrC09 | 21.685 | 2598326 |
| chrC09 | 21.889 | 2815426 |
| chrC09 | 22.104 | 2773920 |
| chrC09 | 22.478 | 3381746 |
| chrC09 | 22.558 | 3410675 |
| chrC09 | 22.692 | 3416417 |
| chrC09 | 23.495 | 3592360 |
| chrC09 | 25.1 | 4040332 |
| chrC09 | 25.406 | 4658640 |
| chrC09 | 25.492 | 38626893 |
| chrC09 | 25.492 | 34109172 |
| chrC09 | 25.492 | 4477359 |
| chrC09 | 25.518 | 4485011 |
| chrC09 | 25.674 | 5187223 |
| chrC09 | 27.427 | 6278396 |
| chrC09 | 27.603 | 5815413 |
| chrC09 | 27.647 | 5898118 |
| chrC09 | 27.803 | 6245416 |
| chrC09 | 27.906 | 6588497 |
| chrC09 | 28.115 | 6553510 |
| chrC09 | 28.232 | 6554181 |
| chrC09 | 29.237 | 5815587 |
| chrC09 | 29.318 | 6102705 |
| chrC09 | 30.204 | 6480906 |
| chrC09 | 43.12 | 13034801 |
| chrC09 | 59.471 | 21242296 |
| chrC09 | 60.502 | 21402677 |
| chrC09 | 62.474 | 35349715 |
| chrC09 | 64.177 | 36027447 |
| chrC09 | 66.436 | 37204606 |
| chrC09 | 67.155 | 37492276 |
| chrC09 | 69.176 | 38670545 |
| chrC09 | 69.176 | 38667626 |
| chrC09 | 72.019 | 39481501 |
| chrC09 | 77.438 | 6613386 |
| chrC09 | 77.438 | 40496478 |
| chrC09 | 77.564 | 40411966 |
| chrC09 | 81.837 | 41122001 |
| chrC09 | 82.653 | 41176086 |
| chrC09 | 83.011 | 41179993 |
| chrC09 | 83.699 | 41269295 |
| chrC09 | 84.28 | 41281291 |
| chrC09 | 84.604 | 41449880 |
| chrC09 | 92.555 | 43647318 |
| chrC09 | 96.998 | 43727749 |
| chrC09 | 104.605 | 45219605 |
